# The association of ADHD and ADHD medication with adherence to pharmacotherapy for type 2 diabetes: a population-based cohort study from seven countries

**DOI:** 10.1016/j.eclinm.2026.104182

**Published:** 2026-08-31

**Authors:** Yiling Zhou, Honghui Yao, Tian Xie, Malcolm B. Gillies, Yanli Zhang-James, Isabell Brikell, Anders Engeland, Le Gao, Birgitte Dige Semark, Theresa Wimberley, Aske Astrup, Zihan Dong, Dina Sarsembayeva, Melissa Vos, Andrew S.C. Yuen, Søren Dalsgaard, Stephen V. Faraone, Kari Klungsøyr, Henrik Larsson, Kenneth K.C. Man, Ian C.K. Wong, Helga Zoega, Harold Snieder, Zheng Chang, Catharina A. Hartman

**Affiliations:** a Department of Epidemiology, University of Groningen, University Medical Center Groningen, Groningen, Netherlands; b Department of Psychiatry, National Clinical Research Center for Mental Disorders, The Second Xiangya Hospital of Central South University, Changsha, 410011, Hunan, China; c Department of Medical Epidemiology and Biostatistics, Karolinska Institutet, Stockholm, Sweden; d Department of Psychiatry, Interdisciplinary Center of Psychopathology and Emotion Regulation, University of Groningen, University Medical Center Groningen, Groningen, Netherlands; e Guangzhou National Laboratory, Guangzhou International Bio Island, Guangzhou, 510005, Guangdong Province, China; f School of Population Health, Faculty of Medicine and Health, University of New South Wales, Sydney, NSW, Australia; g Department of Psychiatry and Behavioral Sciences, Norton College of Medicine, SUNY Upstate Medical University, Syracuse, NY, USA; h Department of Global Public Health and Primary Care, University of Bergen, Bergen, Norway; i Department of Biomedicine, Aarhus University, Aarhus, Denmark; j Department of Chronic Diseases, Norwegian Institute of Public Health, Bergen, Norway; k Department of Pharmacy Administration, School of Pharmacy, Xi'an Jiaotong University, Xi’an, Shaanxi, China; l Centre for Safe Medication Practice and Research, Department of Pharmacology and Pharmacy, Li Ka Shing Faculty of Medicine, The University of Hong Kong, Hong Kong Special Administrative Region, China; m National Centre for Register-based Research, Aarhus University, Aarhus, Denmark; n Centre for Integrated Register-based Research, Aarhus University, Aarhus, Denmark; o Research Department of Practice and Policy, School of Pharmacy, University College London, London, UK; p Centre for Medicines Optimisation Research and Education, University College London Hospitals NHS Foundation Trust, London, UK; q Child and Adolescent Mental Health Center, Copenhagen University Hospital – Mental Health Services CPH, Copenhagen, Denmark; r Department of Clinical Medicine, University of Copenhagen, Copenhagen, Denmark; s Department of Health Promotion, Norwegian Institute of Public Health, Bergen, Norway; t School of Medical Sciences, Örebro University, Örebro, Sweden; u Medical Sciences Division, School of Pharmacy, Faculty of Medicine, Macau University of Science and Technology, Macao Special Administrative Region, China; v Aston Pharmacy School, Aston University, Birmingham, UK; w Centre of Public Health Sciences, Faculty of Medicine, University of Iceland, Reykjavik, Iceland

**Keywords:** ADHD, ADHD medication, Antidiabetic medication adherence, Type 2 diabetes, Multinational cohort study

## Abstract

**Background:**

Attention-deficit/hyperactivity disorder (ADHD) may compromise adherence to pharmacotherapy in adults with type 2 diabetes, but the relationship is unknown. We investigated the association of ADHD and ADHD medication with antidiabetic medication adherence in multinational cohort data.

**Methods:**

Using a common analytical protocol and population-based databases from seven countries, we identified adults initiating non-insulin antidiabetic medication between 2010 and 2020. ADHD was identified by diagnosis or ADHD medication use. We used the proportion of days covered (PDC) to measure antidiabetic medication adherence and defined the primary outcome, poor adherence, as PDC <80% over 1-, 2-, and 5-year follow-up periods. Time-to-first-discontinuation was the secondary outcome. Country-specific estimates were obtained using logistic and Cox regression and pooled using random-effects meta-analyses.

**Findings:**

Among 3,643,197 individuals included, 1,871,870 (51.4%) were female and 88,339 (2.4%) had ADHD (76,973 [87.1%] received ADHD medication). Overall, ADHD was not associated with poor adherence to antidiabetic medication (1-, 2-, and 5-year pooled odds ratios [OR]: 1.01 [95% CI 0.90–1.14], 1.03 [0.91–1.17], and 1.12 [0.95–1.31]); country-specific estimates ranged from 0.79 to 1.32, 0.80 to 1.35, and 0.80 to 1.47 at 1, 2, and 5 years, respectively. Among males, ADHD was associated with poorer adherence (1.09 [95% CI 1.00–1.20], 1.12 [1.00–1.24], and 1.23 [1.11–1.37] at 1, 2, and 5 years). Similar results were found for discontinuation. Among individuals with ADHD, ADHD medication use was consistently associated with better adherence over 1-, 2-, and 5-year follow-up periods (e.g., 5-year pooled OR 0.45 [0.30–0.67]); associations were directionally consistent across countries.

**Interpretation:**

Among adults with type 2 diabetes, ADHD may not necessarily compromise antidiabetic medication adherence when appropriately managed in routine care. ADHD pharmacological treatment may support adherence among adults with comorbid ADHD and type 2 diabetes, although its causal effect requires further evaluation.

**Funding:**

EU Horizon 2020 Research and Innovation Programme.

**Research in context**

**Evidence before this study**

We conducted a systematic search of PubMed up to January 1, 2026, for studies on the association between attention-deficit/hyperactivity disorder (ADHD) and adherence to pharmacotherapy for type 2 diabetes, using the following search terms: ((“Attention Deficit Disorder with Hyperactivity”[Mesh] OR “ADHD”[tiab] OR “attention deficit” [tiab] OR “attention-deficit”[tiab]) AND (“Diabetes Mellitus, Type 2”[Mesh] OR “Hypoglycemic Agents”[Mesh] OR “Metformin”[Mesh] OR “type 2 diabetes”[tiab] OR “T2DM”[tiab] OR “antidiabetic∗”[tiab] OR “anti-diabetic∗”[tiab] OR “glucose-lowering medications”[tiab] OR “glucose-lowering agents”[tiab] OR “diabetes medication∗”[tiab] OR “SGLT2i”[tiab] OR “SGLT-2 inhibitor∗”[tiab] OR “gliflozin∗”[tiab] OR “GLP-1 receptor agonist∗”[tiab] OR “GLP1RA”[tiab]) AND (“Medication Adherence”[Mesh] OR “Patient Compliance”[Mesh] OR “adherence”[tiab] OR “nonadherence”[tiab] OR “non-adherence”[tiab] OR “persistence”[tiab] OR “nonpersistence”[tiab] OR “non-persistence”[tiab] OR “discontinuation”[tiab] OR “medication possession ratio”[tiab] OR “MPR”[tiab] OR “proportion of days covered”[tiab] OR “PDC”[tiab])). The search yielded five articles, only one of which addressed adherence to pharmacotherapy for type 2 diabetes. However, that study included only three individuals with ADHD and examined only insulin discontinuation. Thus, the association between ADHD and antidiabetic medication adherence, and the potential role of ADHD medication, remained unaddressed.

**Added value of this study**

This large multinational cohort study provides real-world evidence on the relationship between ADHD, ADHD medication use, and diabetes medication adherence among adults with type 2 diabetes across seven healthcare settings. Although ADHD symptoms could plausibly interfere with long-term medication-taking, clinically recorded ADHD was not associated with poorer antidiabetic medication adherence overall. However, males with ADHD showed poorer adherence than males without, suggesting a subgroup that may require closer monitoring. Among individuals with ADHD, ADHD medication use was consistently associated with better antidiabetic medication adherence across countries, age and sex subgroups, and follow-up periods. By examining both ADHD status and ADHD medication use, this study suggests that diabetes medication adherence in adults with comorbid ADHD and type 2 diabetes is not determined by ADHD status alone, but may depend on whether ADHD is appropriately managed.

**Implications of all the available evidence**

These findings support a more integrated approach to diabetes and ADHD care. In diabetes practice, ADHD diagnosis alone should not be assumed to indicate poor medication adherence or to require fundamentally different diabetes management strategies. However, adherence should be monitored carefully, particularly among males with ADHD and those with emerging adherence difficulties. When adherence difficulties are identified, coordinated care between diabetes clinicians, primary care providers, and mental health specialists may support both ADHD and diabetes self-management. In ADHD practice, effective ADHD management may be relevant not only for psychiatric symptoms and functioning, but also for the self-management of chronic physical conditions requiring sustained treatment routines. Future studies are needed to clarify whether the observed association between ADHD medication use and better antidiabetic medication adherence is causal, and whether improved antidiabetic medication adherence translates into better glycaemic control and long-term diabetes outcomes.

## Introduction

Type 2 diabetes is a leading cause of mortality and disability worldwide, affecting over 500 million individuals.^1^ Effective management relies on consistent adherence to pharmacological treatment and lifestyle modifications,^2^^,^^3^ yet adherence to antidiabetic medications is often suboptimal,^4^ leading to poor glycaemic control and an increased risk of long-term complications and mortality.^2^

Attention-deficit/hyperactivity disorder (ADHD) is a childhood-onset neurodevelopmental disorder that often persists into adulthood.^5^ It is associated with higher incidence of type 2 diabetes.^5^ Further, ADHD may lead to a worse prognosis as individuals with ADHD tend to develop type 2 diabetes at a younger age,^6^ increasing the risk of complications,^7^ and they also have a higher prevalence of lifestyle-related cardiovascular risk factors.^5^ Core symptoms of ADHD^5^ may directly undermine the ability to maintain a consistent, long-term medication regimen required for diabetes care.^2^^,^^4^ This may lead to poor glycaemic control among individuals with ADHD.^8^

ADHD medications, including stimulants and non-stimulants, are effective in controlling symptoms of ADHD.^9^ However, the addition of ADHD medication may also complicate medication regimens and increase the potential for drug–drug interactions and adverse effects, which may in turn lead to poor adherence to antidiabetic medications.^5^ In addition, individuals with ADHD often present with multiple psychiatric and physical comorbidities,^5^^,^^10^ which may further complicate adherence due to symptoms of comorbid conditions, polypharmacy, and complex medication regimens.

To date, only one study has reported the association between ADHD and adherence to pharmacotherapy for type 2 diabetes; however, this study included only three individuals with ADHD and examined only insulin.^11^ Therefore, the influence of ADHD and ADHD medication on adherence to pharmacotherapy for type 2 diabetes remains unclear but is pertinent to management strategies to reduce complications and premature mortality.^2^ This multinational cohort study, using patient-level data from seven countries, aimed to investigate (1) whether ADHD is associated with poorer diabetes medication adherence among new users of non-insulin antidiabetic medications, and (2) whether ADHD medication use is associated with improved adherence to antidiabetic medications among individuals with ADHD.

## Methods

### Study design and data sources

This multinational, population-based cohort study used a distributed network approach^12^ with a common data model and analytical protocol across seven countries. We used pseudonymized patient-level healthcare data from the MedIntel Data Platform providing statewide administrative and insurance claims data in New South Wales, Australia^13^; nationwide registers and administrative databases in Denmark, the Netherlands, Norway, and Sweden^14^; The Health Improvement Network [THIN], covering approximately 6% of the UK population and broadly representative of key demographic and health indicators^15^; and TriNetX, a federated electronic health record (EHR) network covering more than 60 US healthcare organisations.^16^ Details of data sources are provided in Supplementary Methods. Medication identification was based on pharmacy dispensations in Australia, Denmark, the Netherlands, Norway, and Sweden; and on prescription records in the UK and US (for simplicity, dispensations/prescriptions are called dispensations hereafter).^14^ Antidiabetic medication dispensing data were available from January 1, 2009, to December 31, 2020, with the following country-specific variations: Australia (July 1, 2012–December 31, 2020), and Norway (January 1, 2009–December 31, 2019).

### Ethics

Ethical review of, or approval for the use of, each data source was obtained by the contributing authors and ethics committees in each participating country or region. Informed consent was not required for the data sources used. Details are provided in Supplementary Note S1.

### Study population

Eligible individuals were adults aged ≥18 years who initiated non-insulin antidiabetic medication (WHO Anatomical Therapeutic Chemical [ATC] code A10B) between January 1, 2010, and December 31, 2020 (country-specific study periods as detailed above); the initiation date was defined as baseline. This cohort-entry criterion aligns with treatment guidelines for type 2 diabetes and minimises the inclusion of individuals with type 1 diabetes who typically initiate insulin (ATC code A10A). To define a new user cohort, we applied a washout period of at least 12 months with no records of any antidiabetic medication use (ATC codes A10A or A10B) before baseline. We excluded individuals who, before or at baseline, had emigrated, died or had a diagnosis of type 1 diabetes, where data were available (Supplementary Table S1), and those with missing data on sex or date of birth. Follow-up started at baseline and ended at the outcome of interest, death, emigration, or the administrative end of the study period (i.e., December 31, 2019 in Norway, and December 31, 2020 in all other countries), whichever occurred first. In the US, follow-up was additionally censored at the last available record date in TriNetX to account for individuals leaving participating healthcare organisations. For analyses of ADHD medication, we restricted the cohort to individuals with ADHD identified before or at baseline, using the same baseline date and follow-up definition as in the main cohort.

### Measures

#### Antidiabetic medication

During follow-up, all antidiabetic medication dispensations were collected, including oral and non-insulin injectable blood glucose-lowering drugs (ATC code A10B), and insulins and analogues (ATC code A10A) that are also used to treat type 2 diabetes when non-insulin treatment is insufficient.

#### ADHD and ADHD medication use

ADHD was identified by a recorded clinical diagnosis (International Classification of Diseases, 10th Revision [ICD-10] code F90 or equivalent database-specific codes), or at least one ADHD medication dispensation (methylphenidate [N06BA04], amfetamine [N06BA01], dexamfetamine [N06BA02], lisdexamfetamine [N06BA12], atomoxetine [N06BA09], and guanfacine [C02AC02]), or both.^14^

Among individuals with ADHD, treatment duration of each ADHD medication dispensation was unavailable in most participating databases and was therefore estimated from dispensation records following our previous approach (Supplementary Figure S1).^14^ For each dispensation, treatment duration was defined from the dispensation date to the country-age-sex-specific 75th percentile of time to subsequent dispensation (only intervals of ≤180 days). Overlapping coverage, including from early refills, was carried forward to avoid double-counting. Medication switches were not considered as non-use if coverage was continuous. ADHD medication status was then classified as use or non-use over time according to estimated medication coverage.

#### Covariates

Baseline covariates included sex, age and calendar year at baseline, and history of psychiatric comorbidities. Psychiatric comorbidities were identified from the start of data availability in each country up to and including baseline, including anxiety disorders, autism spectrum disorder, bipolar disorder, depressive disorder, eating disorders, intellectual disability, personality disorders, schizophrenia, and substance use disorders, each defined as a separate binary variable (Supplementary Table S2).

#### Outcomes

**Fig. 1:**
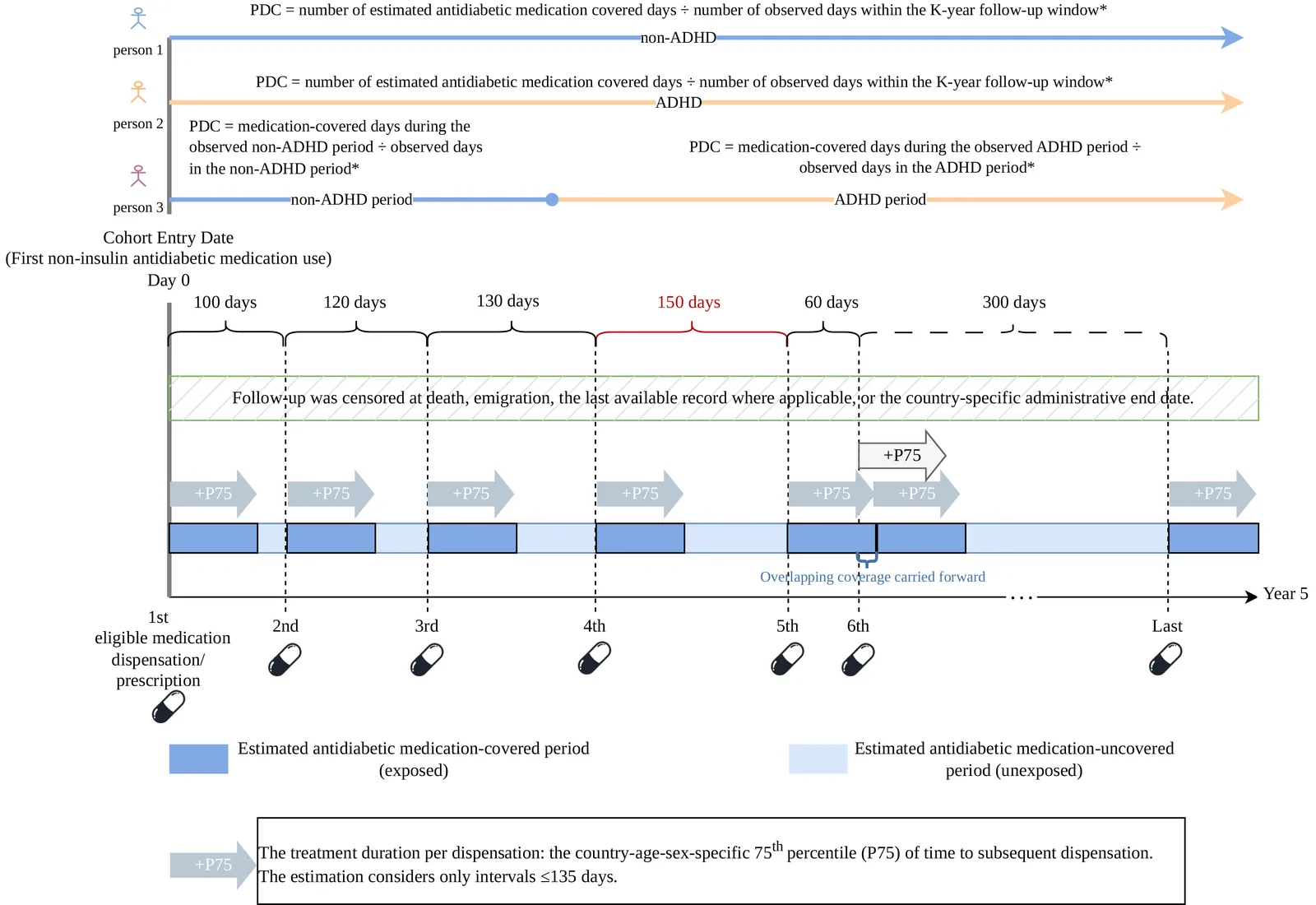
**Schematic illustration of poor adherence to antidiabetic medication.** PDC, proportion of days covered. Daily antidiabetic medication coverage was estimated from pharmacy dispensations in Australia, Denmark, the Netherlands, Norway, and Sweden, and from prescription records in the UK and US. The initial treatment was non-insulin antidiabetic medication; subsequent treatment could include any antidiabetic medication, including insulin. For each dispensation or prescription, treatment duration was estimated as the country-age-sex-specific 75th percentile of the time to subsequent dispensation, using intervals of ≤135 days that indicated continuous use. Overlapping coverage from early refills or different antidiabetic drug classes was carried forward to avoid double-counting. Days within estimated coverage periods were classified as on medication. ∗PDC was calculated as the sum of on-medication days divided by total days in each follow-up period, censored at death, emigration, or the end of the study period. Poor adherence was defined as PDC <80%. ADHD was modelled as a time-varying exposure; when ADHD was first identified within a follow-up window, the window was split at the identification date and PDC was calculated separately for the non-ADHD and ADHD periods.

The primary outcome was poor adherence to antidiabetic medication over 1-, 2-, and 5-year follow-up periods (Fig. 1), measured using the proportion of days covered (PDC).^17^^,^^18^ Treatment duration of each dispensation was unavailable in most databases and was therefore estimated from dispensation records. We defined continuous use as consecutive dispensations with an interval of ≤135 days, based on an assumed typical 90-day prescription length under national healthcare regulations plus a 45-day grace period (one-half of the prescription length) to account for refill variability while avoiding classification of long treatment interruptions as continuous use.^19^ For each dispensation, treatment duration was estimated as the country-age-sex-specific 75th percentile of time to subsequent dispensation (using only intervals of ≤135 days).

For each follow-up period, the PDC numerator was the sum of days covered by any antidiabetic medication. Overlapping treatment periods, either from early refills or different antidiabetic drug classes, were carried forward to avoid double-counting. The PDC denominator was the total days in the corresponding follow-up period, censored at death, emigration, or the end of the study period.^17^^,^^18^ Poor adherence (binary variable, yes vs no) was defined as PDC <80%.^17^

The secondary outcome was time to first discontinuation of any antidiabetic medication within five years of baseline (Supplementary Figure S2). Discontinuation was defined as a gap of >135 days between consecutive dispensations.^17^ Medication switches were not classified as discontinuations. Time to discontinuation was calculated from initiation to the estimated end date of the last dispensation before the discontinuation gap (Supplementary Figure S2).

### Statistical analysis

We performed analyses locally in each participating site using a common study protocol (preregistered in https://doi.org/10.17605/OSF.IO/MU4NW) and analytical R script (R version 4.2.3). We then pooled country-specific estimates using random-effects meta-analysis (R version 4.5.1). A two-sided p < 0.05 in Cochran's Q test and I^2^>75% indicated a statistically significant between-country heterogeneity.

#### Analyses at each participating country

For the primary outcome, poor adherence over 1-, 2-, and 5-year follow-up, we used logistic regression with a person-period approach (Fig. 1). ADHD was modelled as a time-varying exposure, with individuals classified as non-ADHD until the date of ADHD identification and as ADHD thereafter. If ADHD status did not change within a follow-up window, PDC was calculated over the entire window. If ADHD was first identified within a window, the window was split at the ADHD identification date. For each exposure-state period, PDC was calculated as days covered by any antidiabetic medication divided by the exposure-state period length, and poor adherence was defined separately within each period.^17^^,^^18^ Robust standard errors accounted for repeated person-period observations within individuals.

Among individuals who had ADHD at baseline, we applied the same person-period approach to estimate the association between time-varying ADHD medication use and poor adherence to antidiabetic medication. We reported odds ratios (ORs) with 95% confidence intervals (CIs).

For the secondary outcome, first discontinuation of any antidiabetic medication within five years after baseline, we estimated unadjusted cumulative incidence using Kaplan-Meier curves and used Cox models to estimate hazard ratios (HRs) with 95% CIs. ADHD was modelled as a time-varying exposure. Instead of assuming proportional hazards, we interpreted the estimates from the Cox models as weighted-average hazard ratios over time.^20^

All main models were adjusted for sex, age and calendar year at baseline. Additional adjustment for psychiatric comorbidities was performed in exploratory analyses. There were no missing data on exposure, outcome, and covariates. We conducted subgroup analyses by baseline age groups (young adults [18–34 years], middle-aged adults [35–64], and older adults [≥65]) and sex (female and male captured from administrative registers or EHRs, representing biological female and male, respectively).

#### Sensitivity analyses

We conducted six sensitivity analyses to assess the robustness of results and explore potential bias. First, we modelled ADHD as a lifetime exposure, i.e., individuals identified with ADHD at any time before the end of the study period were classified as having ADHD throughout follow-up. Second, we redefined ADHD as a fixed baseline exposure, present or absent before baseline. Third, to assess robustness to different sources of ADHD ascertainment, we repeated main analyses using a four-level ADHD exposure: diagnosis only, medication only, both diagnosis and medication, and no ADHD diagnosis or medication as the reference group. Fourth, other prescription lengths and grace periods have been suggested in Australia and the UK. Therefore, we repeated the main analyses using a prescription length of 120 days with a 60-day grace period in Australia, and a prescription length of 90 days with a 56-day grace period in the UK to define the outcomes. Fifth, using the THIN database (UK), which has well-documented primary care type 2 diabetes diagnoses, we restricted the cohort to individuals with a clinical type 2 diabetes diagnosis in two separate analyses: at any time before baseline, or within one year before baseline. This assessed potential misclassification from using initiation of non-insulin antidiabetic medication as a proxy for type 2 diabetes. Sixth, to quantify the potential impact of unmeasured confounding, we calculated E-values for the main pooled estimates.

#### Post-hoc analysis

We conducted three post-hoc analyses to investigate heterogeneity observed in the main results. First, because several country-specific estimates differed from each other, we used best linear unbiased predictions (BLUP) to estimate country-specific deviations from the overall meta-analytic estimate and applied Bonferroni correction for seven country-level tests, and then repeated the meta-analyses after excluding the countries with statistically significant deviations (Supplementary Table S3).^21^ Second, given the relatively high proportion of young adults in the Australian cohort, we performed age-by-sex stratified analyses within Australia to assess whether its sex-stratified findings were partly explained by cohort age structure. Third, to statistically assess whether the associations between ADHD and outcomes differed by age or sex, we fitted models including ADHD-by-age-group and ADHD-by-sex interaction terms.

### Role of funding source

The funders of the study had no role in study design, data collection, data analysis, data interpretation, or writing of the report.

## Results

The final study population included 3,643,197 individuals, of whom 1,871,870 (51.4%) were female and 88,339 (2.4%) had ADHD (Supplementary Table S4). Median follow-up ranged from 1.8 to 6.1 years across countries (Supplementary Table S5). Individuals with ADHD were younger at baseline and had a higher prevalence of psychiatric comorbidities than those without ADHD (Table 1). Among individuals with ADHD (N = 88,339), 39,441 (44.6%) had a recorded ADHD diagnosis and 76,973 (87.1%) had received ADHD medication before the end of study period (Table 1). Ethnicity data were not available.

**Table 1:** Baseline characteristics of the study population by country.

Baseline characteristics	Pooled population		Australia		Denmark		Netherlands		Norway		Sweden		United Kingdom		United States	
	ADHD^a^	Non-ADHD^a^	ADHD^a^	Non-ADHD^a^	ADHD^a^	Non-ADHD^a^	ADHD^a^	Non-ADHD^a^	ADHD^a^	Non-ADHD^a^	ADHD^a^	Non-ADHD^a^	ADHD^a^	Non-ADHD^a^	ADHD^a^	Non-ADHD^a^
N	88,339	3,554,858	5551	421,427	3160	212,100	8490	530,173	1170	23,049	5066	347,964	553	234,043	64,349	1,786,102
ADHD diagnosis^b^, n (%)	39,441 (44.6)	–	0 (0%)	–	1160 (36.7)	–	3153 (37.1)	–	995 (85.0)	–	4292 (84.7)	–	447 (80.8)	–	29,394 (45.7)	–
ADHD medication^b^, n (%)	76,973 (87.1)	–	5551 (100%)	–	2970 (94.0)	–	7608 (89.6)	–	999 (85.4)	–	4185 (82.6)	–	227 (41.0)	–	55,433 (86.1)	–
Age at baseline, years	32–51^c^	38–63^c^	33 [24–45]	54 [39–66]	42 [30–53]	60 [50–70]	51 [42–61]	62 [53–71]	32 [25–40]	38 [30–44]	42 [31–52]	63 [52–71]	40 [28–57]	59 [48–70]	46 [34–58]	59 [49–68]
Age, years, n (%)																
Young adults (18–34)	26,694 (30.2)	310,382 (8.7)	2920 (52.6)	78,240 (18.6)	1070 (33.9)	17,210 (8.1)	1195 (14.1)	14,227 (2.7)	664 (56.8)	9027 (39.2)	1667 (32.9)	17,657 (5.1)	232 (42.0)	22,313 (9.5)	18,946 (29.4)	151,708 (8.5)
Middle-aged adults (35–64)	52,184 (59.1)	1,939,116 (54.5)	2477 (44.6)	227,956 (54.1)	1810 (57.3)	111,710 (52.7)	5735 (67.6)	283,322 (53.4)	506 (43.2)	14,022 (60.8)	3174 (62.7)	172,388 (49.5)	235 (42.5)	123,030 (52.6)	38,247 (59.4)	1,006,688 (56.4)
Older adults (≥65)	9461 (10.7)	1,305,360 (36.7)	154 (2.8)	115,231 (27.3)	280 (8.9)	83,180 (39.2)	1560 (18.4)	232,624 (43.9)	0	0	225 (4.4)	157,919 (45.4)	86 (15.6)	88,700 (37.9)	7156 (11.1)	627,706 (35.1)
Sex, female, n (%)	55,501 (62.8)	1,816,369 (51.1)	3449 (62.1)	234,524 (55.6)	1740 (55.1)	99,670 (47.0)	3695 (43.5)	242,586 (45.8)	772 (66.0)	14,299 (62.0)	2579 (50.9)	155,141 (44.6)	283 (51.2)	112,422 (48.0)	42,983 (66.8)	957,727 (53.6)
Time to end of follow-up, days	848–1886^c^	661–2242^c^	1297 [590–2123]	1491 [670–2327]	1400 [580–2650]	1750 [760–2980]	1590 [707–2744]	1862 [870–2957]	1233 [564–2329]	1355 [625–2421]	1297 [576–2345]	1516 [706–2617]	1886 [966–2885]	2242 [1,189–3138]	848 [346–1522]	661 [226–1345]
At least one psychiatric comorbidity, n (%)	36,331 (41.1)	502,344 (14.1)	1831 (33.0)	41,426 (9.8)	1300 (41.1)	16,900 (8.0)	3145 (37.0)	33,897 (6.4)	696 (59.5)	4694 (20.4)	3893 (76.8)	49,895 (14.3)	351 (63.5)	67,971 (29.0)	25,115 (39.0)	287,561 (16.1)
Anxiety disorder, n (%)	23,962 (27.1)	220,397 (6.2)	862 (15.5)	13,779 (3.3)	340 (10.8)	3180 (1.5)	1000 (11.8)	11,118 (2.1)	302 (25.8)	1934 (8.4)	2434 (48.0)	20,793 (6.0)	76 (13.7)	13,177 (5.6)	18,948 (29.4)	156,416 (8.8)
Autism spectrum disorder, n (%)	1391 (1.6)	3090 (0.1)	126 (2.3)	443 (0.1)	100 (3.2)	240 (0.1)	338 (4.0)	1062 (0.2)	36 (3.1)	111 (0.5)	715 (14.1)	907 (0.3)	41 (7.4)	308 (0.1)	35 (0.1)	19 (0.0)
Bipolar disorder, n (%)	6570 (7.4)	36,662 (1.0)	475 (8.6)	4852 (1.2)	100 (3.2)	1280 (0.6)	269 (3.2)	2678 (0.5)	96 (8.2)	408 (1.8)	755 (14.9)	3718 (1.1)	25 (4.5)	1886 (0.8)	4850 (7.5)	21,840 (1.2)
Intellectual disability, n (%)	1479 (1.7)	10,008 (0.3)	83 (1.5)	1409 (0.3)	80 (2.5)	640 (0.3)	108 (1.3)	891 (0.2)	45 (3.8)	196 (0.9)	297 (5.9)	1397 (0.4)	60 (10.8)	1910 (0.8)	806 (1.3)	3565 (0.2)
Depressive disorder, n (%)	8330 (9.4)	133,759 (3.8)	1065 (19.2)	17,186 (4.1)	640 (20.3)	7890 (3.7)	1620 (19.1)	17,106 (3.2)	361 (30.9)	2563 (11.1)	2393 (47.2)	22,827 (6.6)	252 (45.6)	57,967 (24.8)	1999 (3.1)	8220 (0.5)
Schizophrenia, n (%)	4266 (4.8)	51,537 (1.4)	321 (5.8)	8903 (2.1)	270 (8.5)	3790 (1.8)	306 (3.6)	5499 (1.0)	59 (5.0)	543 (2.4)	545 (10.8)	6557 (1.9)	41 (7.4)	4849 (2.1)	2724 (4.2)	21,396 (1.2)
Personality disorder, n (%)	5748 (6.5)	31,343 (0.9)	570 (10.3)	5166 (1.2)	460 (14.6)	2000 (0.9)	1520 (17.9)	10,733 (2.0)	141 (12.1)	667 (2.9)	1069 (21.1)	5230 (1.5)	37 (6.7)	2447 (1.0)	1951 (3.0)	5100 (0.3)
Eating disorders, n (%)	2367 (2.7)	11,277 (0.3)	115 (2.1)	525 (0.1)	60 (1.9)	360 (0.2)	244 (2.9)	1474 (0.3)	15 (1.3)	219 (1.0)	295 (5.8)	2103 (0.6)	14 (2.5)	1311 (0.6)	1624 (2.5)	5285 (0.3)
Substance use disorder, n (%)	13,029 (14.7)	200,327 (5.6)	833 (15.0)	18,696 (4.4)	440 (13.9)	5300 (2.5)	776 (9.1)	5280 (1.0)	200 (17.1)	838 (3.6)	1615 (31.9)	16,119 (4.6)	48 (8.7)	3115 (1.3)	9117 (14.2)	150,979 (8.5)

In Denmark, due to data privacy concerns, the counts were rounded to the nearest 10.

– Not applicable.

a Lifetime ADHD, ever identified with ADHD before the end of the study period.

b Receiving ADHD diagnosis or at least one ADHD medication before the end of the study period.

c Median range for age across countries, and median range across countries for follow-up.

PDC distributions varied across countries (Supplementary Table S6). Australia, Norway, and the US had lower median PDC values, whereas Denmark, the Netherlands, Sweden, and the UK had higher median PDC values. For example, among individuals without ADHD, median 1-year PDC was 0.40 (IQR 0.17–0.79) in the US, compared with 1.00 (IQR 0.64–1.00) in the Netherlands.

**Fig. 2:**
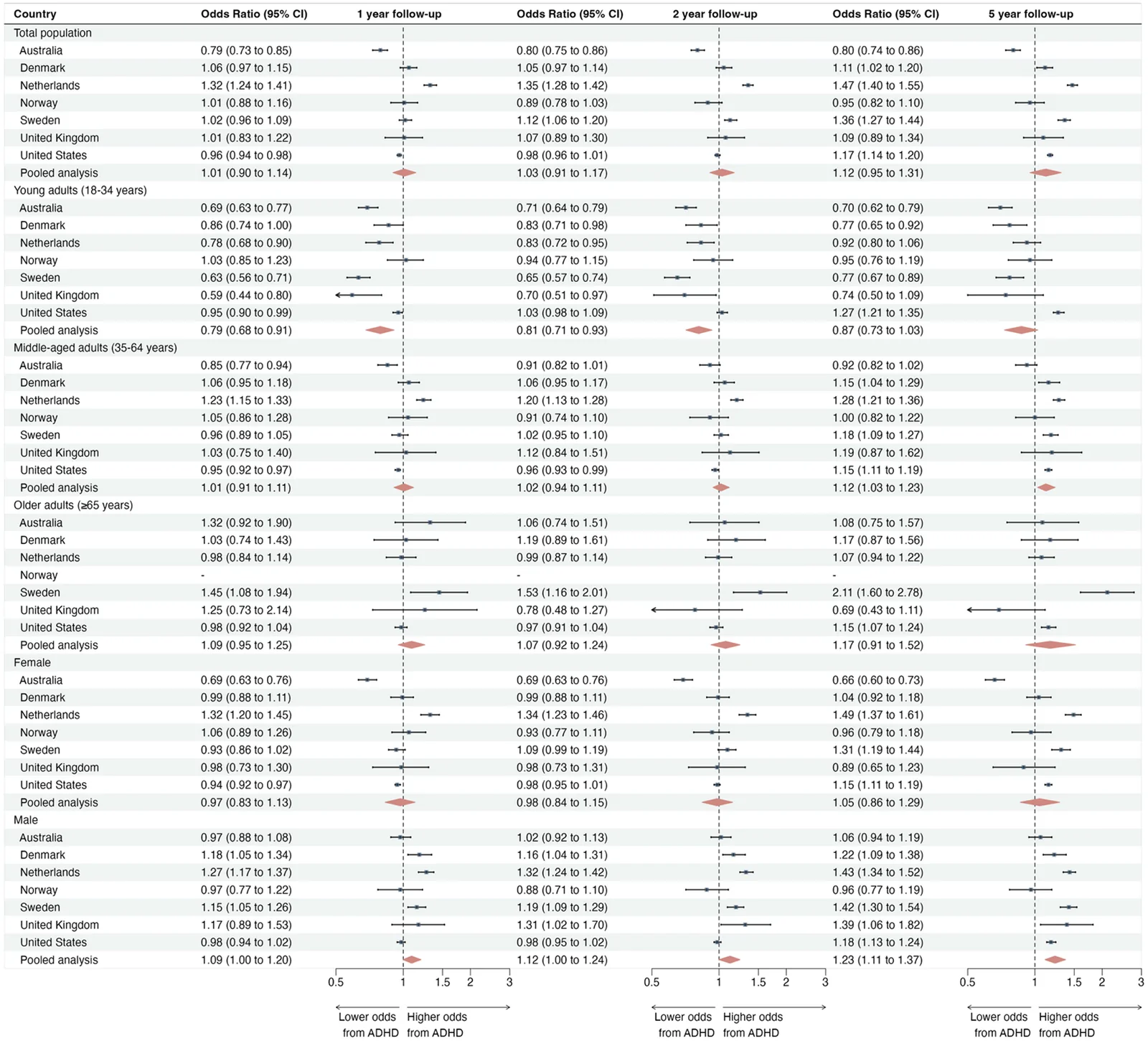
**Pooled and country-specific association between ADHD and poor adherence to antidiabetic medication.** CI, confidence interval. Models adjusted for sex, age and calendar year at initiation. Poor adherence to antidiabetic medication was defined as proportion of days covered (PDC) <80%. Pooled estimates were obtained using random-effects meta-analysis across all seven countries. Medication identification was based on pharmacy dispensations in Australia, Denmark, the Netherlands, Norway, and Sweden; and on prescription records in the UK and US.

In the main analysis adjusted for sex, age and calendar year at baseline, ADHD was not associated with poor adherence to antidiabetic medication across follow-up periods (pooled OR 1.01 [95% CI 0.90–1.14] at 1 year, 1.03 [95% CI 0.91–1.17] at 2 years, 1.12 [95% CI 0.95–1.31] at 5 years; Fig. 2). Country-specific estimates varied, ranging from 0.79 to 1.32 at 1 year, 0.80 to 1.35 at 2 years, and 0.80 to 1.47 at 5 years, with significant between-country heterogeneity (I^2^>75%; p < 0.001; Supplementary Table S7). At 5 years, ADHD was associated with poorer adherence in Denmark (OR 1.11 [95% CI 1.02–1.20]), the Netherlands (OR 1.47 [95% CI 1.40–1.55]), and Sweden (OR 1.36 [95% CI 1.27–1.44]), whereas it was associated with better adherence in Australia (OR 0.80 [95% CI 0.74–0.86]) (Fig. 2).

When stratified by age (Fig. 2), the estimates were more consistent across countries. ADHD was associated with better adherence among young adults (pooled OR 0.79 [95% CI 0.68–0.91] at 1 year, 0.81 [0.71–0.93] at 2 years, and 0.87 [0.73–1.03] at 5 years). Estimates among middle-aged and older adults were close to the null in most countries. When stratified by sex (Fig. 2), among males, estimates were consistently near or above l.0 across countries (pooled OR 1.09 [95% CI 1.00–1.20] at 1 year, 1.12 [95% CI 1.00–1.24] at 2 years, and 1.23 [95% CI 1.11–1.37] at 5 years). Among females, pooled estimates were close to the null: 0.97 (95% CI 0.83–1.13) at 1 year, 0.98 (0.84–1.15) at 2 years, and 1.05 (0.86–1.29) at 5 years, with heterogeneous country-specific estimates. Additional adjustment for psychiatric comorbidities yielded minimal changes to the pooled estimates (Supplementary Figure S3).

Analyses of ADHD medication were conducted among 61,403 individuals identified with ADHD before baseline. Among them, 36,749 (59.8%) were female, and 54,478 (88.7%) had received ADHD medication before the end of study period (Supplementary Tables S8 and S9). Individuals treated with ADHD medication had a higher median PDC than those untreated. For example, in Denmark, median 5-year PDC was 0.74 (IQR 0.28–1.00) among untreated and 0.89 (IQR 0.49–1.00) among treated (Supplementary Table S6).

**Fig. 3:**
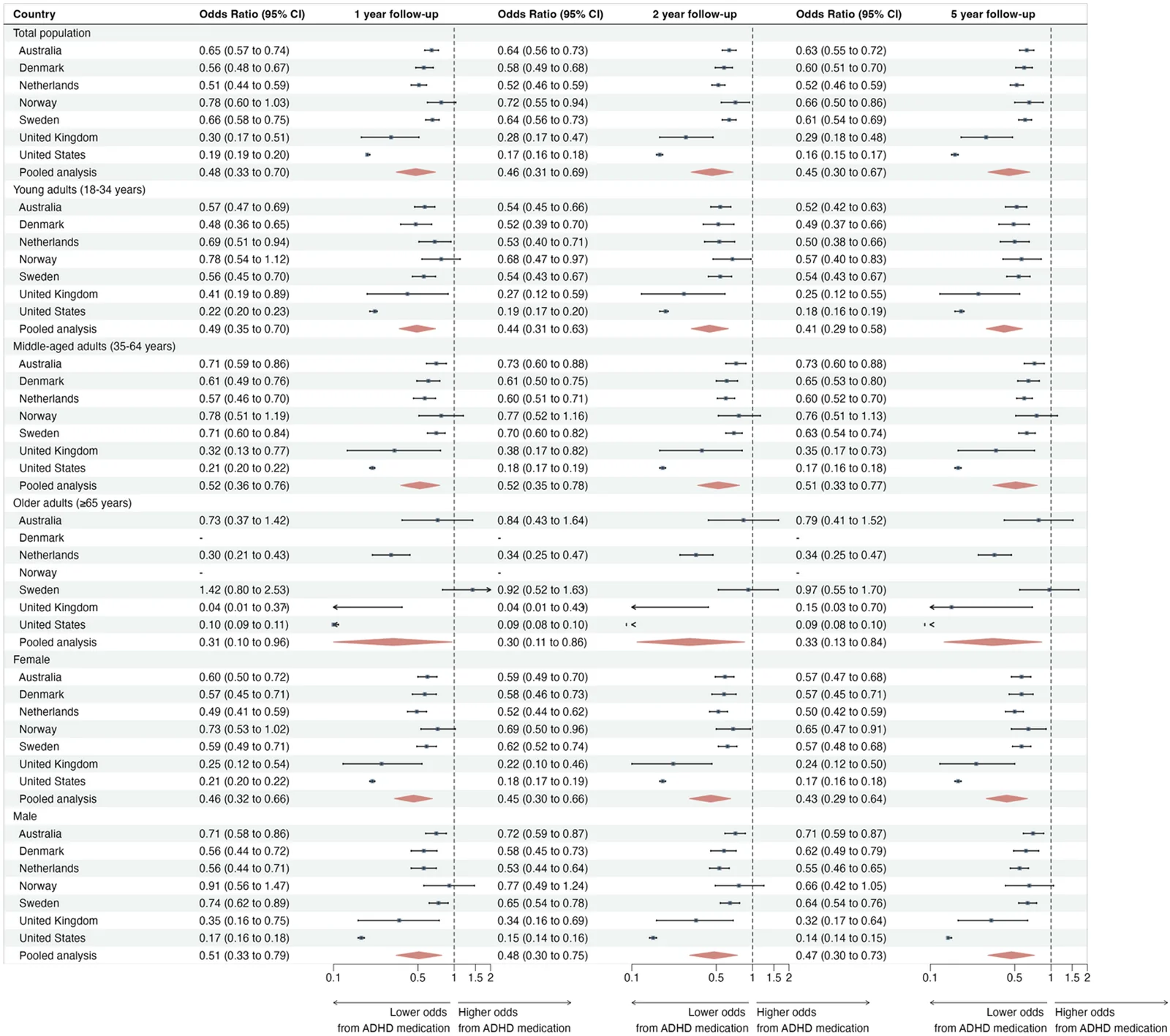
**Pooled and country-specific association between ADHD medication use and poor adherence to antidiabetic medication among individuals with ADHD.** CI, confidence interval. Models adjusted for sex, age and calendar year at initiation. Poor adherence to antidiabetic medication was defined as proportion of days covered (PDC) <80%. Pooled estimates were obtained using random-effects meta-analysis across all seven countries. Medication identification was based on pharmacy dispensations in Australia, Denmark, the Netherlands, Norway, and Sweden; and on prescription records in the UK and US.

In the main analysis adjusted for sex, age and calendar year at baseline, ADHD medication use was consistently associated with better adherence to antidiabetic medication across follow-up periods (pooled OR 0.48 [0.33–0.70] at 1 year, 0.46 [0.31–0.69] at 2 years, 0.45 [0.30–0.67] at 5 years). Associations were directionally consistent across countries (Fig. 3), although between-country heterogeneity was statistically significant (I^2^>75%; p < 0.001; Supplementary Table S7).

Age-, and sex-stratified analyses showed similar associations across subgroups (Fig. 3). Additional adjustment for psychiatric comorbidities resulted in minimal changes to the pooled estimates (Supplementary Figure S4).

**Fig. 4:**
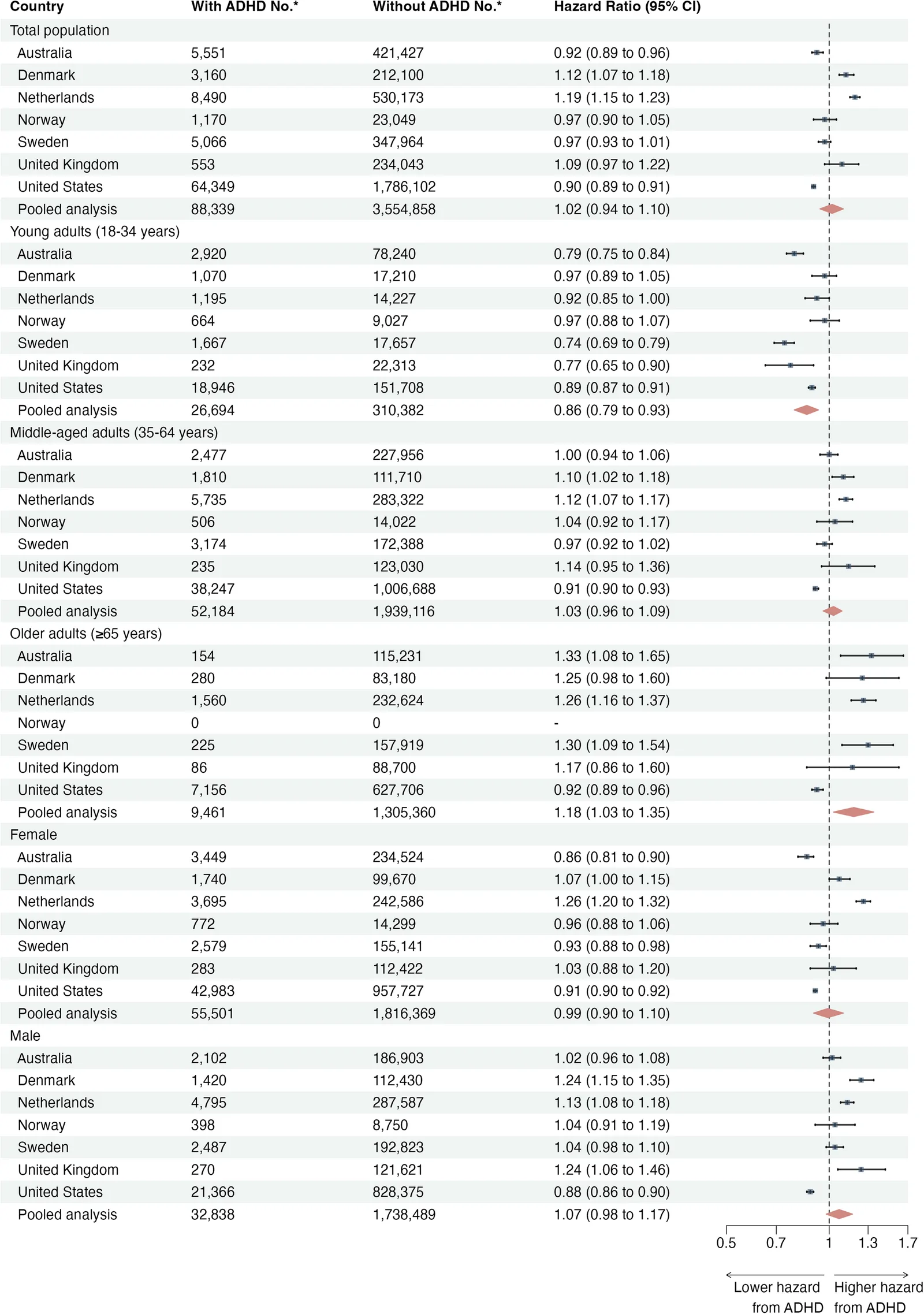
**Pooled and country-specific association between ADHD and the first discontinuation of antidiabetic medication.** CI, confidence interval. Models adjusted for sex, age and calendar year at initiation. ∗No., number of individuals with or without ADHD, respectively, before the end of the study period. The counts are descriptive. Pooled estimates were obtained using random-effects meta-analysis across all seven countries. Medication identification was based on pharmacy dispensations in Australia, Denmark, the Netherlands, Norway, and Sweden; and on prescription records in the UK and US.

The unadjusted cumulative incidence of first discontinuation of antidiabetic medication varied across countries (Supplementary Figure S5). Australia, Norway, and the US showed a pattern of high and early discontinuation in both ADHD and non-ADHD groups, where 1-year risks reached 63%, 64%, and 86%, respectively, and 2-year risks reached 74%, 80%, and 93% in the non-ADHD group. In contrast, discontinuation risks were lower and increased more gradually in Denmark, Sweden, the UK, and particularly in the Netherlands (1-, 2-, and 5-year risk in the non-ADHD group: 28%, 41%, and 59%). In the main analysis adjusted for sex, age and calendar year at baseline, pooled analyses showed no association between ADHD and first discontinuation (HR [95% CI] 1.02 [0.94–1.10]; Fig. 4), with significant between-country heterogeneity (I^2^>75%; p < 0.001; Supplementary Table S7). Subgroup patterns were broadly consistent with those observed for poor adherence (Figs. 2 and 4). Additional adjustment for psychiatric comorbidities resulted in minimal changes to the pooled estimates (Supplementary Figure S6).

### Sensitivity and post-hoc analyses

Sensitivity analyses generally supported the main findings. Modelling ADHD as a lifetime exposure produced results consistent with the main analyses (Supplementary Tables S10 and S11). Defining ADHD as a fixed baseline exposure also supported the main findings (Supplementary Tables S12–S14). Sensitivity analyses by ADHD ascertainment source (diagnosis only, medication only, or both vs no ADHD) supported the main findings and suggested these ascertainment groups may partly represent clinically heterogeneous ADHD populations (Supplementary Tables S15–S17). Results were robust to country-specific prescription-length and grace-period assumptions, including 120 days plus a 60-day grace period in Australia and 90 days plus a 56-day grace period in the UK; the largest point-estimate change was from 1.33 to 1.21 (Supplementary Tables S18–S20). Analyses restricted to individuals with confirmed type 2 diabetes diagnoses in the UK also supported the main findings (Supplementary Tables S21 and S22). E-values indicated that the associations in ADHD analyses could potentially be explained by unmeasured confounding of moderate strength (E-values ranging from 1.08 to 1.31 across follow-ups among the total population), whereas an unmeasured confounder of strong strength would be required to fully explain the association between ADHD medication use and antidiabetic medication adherence (E-values ranging from 2.24 to 2.35 across follow-ups among the total population), conditional on sex, age, and calendar year at initiation (Supplementary Table S23).

Post-hoc BLUP analyses identified significant deviations from the overall pooled associations for Australia in ADHD–poor adherence analyses, particularly in the total population and among females, and for the US in ADHD–discontinuation and ADHD medication–poor adherence analyses (Supplementary Table S3). Excluding these deviating estimates had minimal impact on the pooled results (Supplementary Figures S6–S8). Age-by-sex stratified analyses within Australia showed similar associations in females and males in each age group (Supplementary Table S24). Consistent with the age- and sex-stratified estimates, formal interaction tests showed evidence of ADHD-by-age-group interaction in most countries, except Norway and the US. ADHD-by-sex interaction was significant in Australia, the Netherlands, and the US at 1 and 2 years, but at 5 years only in Australia and the Netherlands (Supplementary Table S25).

## Discussion

In this large multinational cohort study of adults with type 2 diabetes, ADHD was not associated with poor antidiabetic medication adherence or discontinuation overall, although males with ADHD may require closer monitoring. Although country-specific estimates varied, as expected in multinational real-world data, the pooled associations were not driven by any single country's estimate. Among individuals with ADHD, ADHD medication use was consistently associated with better antidiabetic medication adherence across countries, subgroups, and follow-up periods.

Type 2 diabetes requires long-term pharmacological treatment and consistent self-management.^2^^,^^3^ ADHD symptoms, including inattention and impulsivity, could plausibly impair self-management.^4^^,^^5^^,^^22^^,^^23^ However, the results suggest that ADHD may not necessarily compromise antidiabetic medication adherence when appropriately managed in routine care. One potential explanation is that most individuals with ADHD in our study received ADHD medication (87.1%), which may have mitigated ADHD symptoms and supported sustained medication-taking. Clinically, the findings are reassuring: an ADHD diagnosis should not be assumed to indicate poor antidiabetic medication adherence or to require fundamentally different diabetes management strategies. Instead, attention should be directed toward ensuring adequate recognition and management of ADHD as part of integrated, long-term care. Among individuals with both ADHD and type 2 diabetes, ADHD medication use was associated with better antidiabetic medication adherence. This finding raises the possibility that ADHD medication treatment may support the management of chronic physical conditions requiring sustained self-care. This interpretation, however, remains tentative, because residual confounding cannot be excluded, including by ADHD heterogeneity,^5^^,^^24^ whereby individuals with different manifestations or behavioural profiles of ADHD differ in their adherence to any treatment, and by healthcare access, which could influence access to both ADHD and antidiabetic medications. Cardiovascular health may also confound the association, as it could influence decisions to stop, reduce, or restart ADHD medication,^25^ and may affect antidiabetic medication adherence through care intensity or comorbidity burden. Although E-value sensitivity analyses suggested that a strong unmeasured confounder would be required to fully explain the observed association conditional on measured covariates, further causal evaluation is needed. For clinical practice, these findings suggest that clinicians may need to assess whether ADHD symptoms are adequately managed and, if not, consider coordinated ADHD care to support both ADHD and diabetes self-management. If ADHD medication is unwarranted, support for diabetes self-management should likewise be considered.

Country-specific estimates varied. Differences across countries in healthcare systems, reimbursement structures, clinical guidelines, regulations, prescribing practices, access to ADHD and diabetes medications, and follow-up care may all contribute to this.^14^^,^^26^ We interpreted this heterogeneity as reflecting real-world contextual differences across countries rather than inconsistency in the findings such that they could not be trusted. Importantly, excluding potentially outlying country-specific estimates resulted in minimal changes to the pooled results, indicating that main conclusions were not materially influenced by any single country. The random-effects model was used to allow for between-country variation; therefore, pooled estimates should be interpreted as average associations across countries and considered alongside the country-specific estimates, rather than as uniform associations across countries.

Subgroup analyses provided some preliminary insights. Males with ADHD showed slightly higher odds of poor adherence than males without ADHD, particularly over longer follow-up, with an OR of 1.23 at 5 years. These patterns may reflect sex- and gender-related differences in ADHD presentation, compensatory strategies, and related behaviours.^5^ For example, compensatory strategies used by some females with ADHD to meet social expectations, e.g., spending a lot of mental energy to appear organised and socially compliant, could help maintain routines such as medication taking. By contrast, males with ADHD may be more likely to show externalising symptoms and persistent struggles with organisation and routine tasks,^27^ which could interfere with long-term medication adherence. We also observed better adherence associated with ADHD among young adults. One possible explanation is type 2 diabetes misclassification in this age group. Type 2 diabetes is usually diagnosed in primary care, but primary care diagnoses were unavailable in most databases; we therefore used initiation of non-insulin antidiabetic medication after a washout period as a proxy for newly diagnosed type 2 diabetes. This approach may have included some young adults without type 2 diabetes, particularly females prescribed metformin for polyendocrine metabolic ovarian syndrome or gestational diabetes. Inclusion of these non-diabetes indications could have influenced adherence patterns in young adults. Sensitivity analyses restricted to individuals with confirmed type 2 diabetes diagnoses in the UK suggested that this source of bias was unlikely to materially affect overall findings or findings among middle-aged and older adults, or males, although estimates among young adults were less precise because of the small sample size. Further studies in young adults with confirmed type 2 diabetes are needed.

Several limitations warrant consideration. First, ADHD may have been misclassified because adult ADHD can present heterogeneously, with variable symptoms, functional impairment, compensatory strategies, and comorbidities that may obscure diagnosis.^5^^,^^27^ Country-specific clinical practices, cultural attitudes toward ADHD, and healthcare access may have further affected ADHD ascertainment across databases.^14^^,^^26^ Second, diabetes remission could not be identified in the participating databases; we therefore could not distinguish medication discontinuation due to non-adherence from clinically appropriate discontinuation after remission. Because remission remains uncommon in routine care,^28^ major bias from remission is unlikely. Third, treatment duration was unavailable in most databases and was therefore estimated from dispensation patterns.^14^^,^^19^ This may have introduced some measurement error, particularly for discontinuation, which depended on a predefined gap between consecutive dispensations. However, our primary outcome, poor adherence based on PDC, was less dependent on any single gap definition and was therefore more robust. For this reason, we focused on poor adherence as the primary outcome. In analyses of ADHD medication, exposure was also estimated from dispensation records; therefore, we did not examine ADHD medication in relation to discontinuation, to avoid compounding uncertainty in both exposure and outcome measurement. Finally, as our study included data only from high-income countries, the generalisability of our findings to low- and middle-income settings may be limited.

This large multinational observational study showed that ADHD was not associated with poorer adherence to antidiabetic medication among individuals with type 2 diabetes overall, while males with ADHD may require closer monitoring. Among individuals with both type 2 diabetes and ADHD, ADHD medication use was consistently associated with better antidiabetic medication adherence. These findings suggest that ADHD itself should not be assumed to compromise diabetes medication adherence when appropriately recognised and managed in routine care. Instead, clinicians should monitor adherence when difficulties emerge and consider coordinated psychiatric, primary, and diabetes care to support long-term self-management. ADHD medication treatment may support antidiabetic medication adherence, but whether this association is causal, and whether improved adherence translates into better glycaemic control and long-term diabetes outcomes, require further evaluation.

## Contributors

YZ, HY, ZC, MBG, YZ-J, IB, AE, LG, KKCM, AA, BDS, TW, and TX directly accessed and verified the country or region-specific data and performed data curation and formal analysis of the country or region-specific data underlying the meta-analyses reported in the manuscript. YZ and HY did all meta-analyses and visualisation of results presented under the supervision of HZ, HS, ZC, and CH. YZ, HY, MBG, YZ-J, HZ, ZC, and CH wrote the original draft. HL, IB, SD, SVF, ICKW, KK, HZ, ZC, and CH contributed to funding acquisition. All authors confirm that the authors responsible for each country-specific analysis had full access to the relevant country-specific data in the study. All authors had access to the aggregate estimates used in the meta-analyses. All authors approved the final version of the manuscript and had final responsibility for the decision to submit it for publication.

## Data sharing statement

Country-specific regulations and laws prohibit sharing or making the individual-level data in this study publicly available. Access to these data is not possible without the permission of the relevant approving human research ethics committees or the data custodians. Details and country-specific data sharing statements are provided in Supplementary Note S1.

## Declaration of interests

HL reports receiving grants from TAKEDA and Shire Pharmaceuticals; personal fees from and serving as a speaker for Medice, Shire/Takeda Pharmaceuticals and Evolan Pharma AB; all outside the submitted work. HL is editor-in-chief of JCPP Advances.

SVF reports personal fees and travel expenses from Mentavi/ADHD Online, Axsome, Ironshore/Collegium, KemPharm/Corium, Noven, Supernus, Sandoz, Tris and Medice; has a US patent (via his institution) for the use of sodium-hydrogen exchange inhibitors in the treatment of ADHD (US20130217707 A1); receives royalties from books published by Guilford Press (Straight Talk about Your Child's Mental Health), Oxford University Press (Schizophrenia: The Facts), and Elsevier (ADHD: Non-Pharmacologic Interventions); and is program director of www.ADHDEvidence.org and www.ADHDinAdults.com.

ICKW reports research funding from Amgen and GSK, the Hong Kong Research Grants Council, the Hong Kong Health and Medical Research Fund, the National Institute for Health Research in England and the European Commission, consulting fees from IQVIA and World Health Organization, outside of the submitted work. He is a non-executive director of Jacobson Medical, Advance Data Analytics for Medical Science (ADAMS) Limited in Hong Kong and OCUS Innovation Limited (HK, Ireland and UK); a former director of Therakind in England and Asia Medicine Regulatory Affairs (AMERA) Services Limited in Hong Kong.

ZC received lecture honoraria from TAKEDA Pharmaceuticals, outside the submitted work.

ASCY reports grants from the University College London Hospitals NIHR Biomedical Research Centre, College of Mental Health Pharmacy, and UK Turing Scheme.

KKCM reports grants from CW Maplethorpe Fellowship, IQVIA, the European Union Horizon 2020, the UK NIHR and the Hong Kong Research Grant Council and Hong Kong Innovation and Technology Commission.

MBG reports that the Medicines Intelligence Research Program, School of Population Health, UNSW has received research funding from IQVIA Australia, unrelated to this project.

All other authors declare no competing interests.
