# Supplementary material for: The association of ADHD and ADHD medication with adherence to pharmacotherapy for type 2 diabetes: a population-based cohort study from seven countries
Source: eClinicalMedicine. 2026 Aug 31;99:104182. doi: 10.1016/j.eclinm.2026.104182 (PMC13559831; doi:10.1016/j.eclinm.2026.104182)
Supplement: Supplementary file 1 [file mmc1.docx]

Table of Contents

[Supplemental methods	3](#_Toc236735149)

[Data sources	3](#_Toc236735150)

[Supplemental note 1. Ethical review and data sharing summary.	4](#_Toc236735151)

[Supplementary table 1. Data source information by country/region.	5](#_Toc236735152)

[Supplementary table 2. Definitions of covariates.	6](#_Toc236735153)

[Supplementary table 3. Country-specific deviations from pooled estimates after Bonferroni correction.	7](#_Toc236735154)

[Supplementary table 4. Exclusion criteria by country/region.	11](#_Toc236735155)

[Supplementary table 5. Baseline characteristics of the pooled population, stratified by ADHD.	12](#_Toc236735156)

[Supplementary table 6. Median (25th percentile, 75th percentile) proportion of days covered (PDC) for antidiabetic medication by subgroups across seven countries.	13](#_Toc236735157)

[Supplementary table 7. Statistical test for between-country heterogeneity.	18](#_Toc236735158)

[Supplementary table 8. Number of individuals included by subgroups.	20](#_Toc236735159)

[Supplementary table 9. Baseline characteristics among individuals identified with ADHD before baseline across seven countries.	21](#_Toc236735160)

[Supplementary table 10. Sensitivity analyses modelling ADHD as lifetime ADHD: association between ADHD and poor adherence to antidiabetic medication.	22](#_Toc236735161)

[Supplementary table 11. Sensitivity analyses modelling ADHD as lifetime ADHD: association between ADHD and first discontinuation of antidiabetic medication.	23](#_Toc236735162)

[Supplementary table 12. The number and proportion for individuals with ADHD identified before baseline versus during follow-up.	24](#_Toc236735163)

[Supplementary table 13. Sensitivity analyses redefining ADHD as a baseline exposure: association between ADHD and poor adherence to antidiabetic medication.	25](#_Toc236735164)

[Supplementary table 14. Sensitivity analyses redefining ADHD as a baseline exposure: association between ADHD and first discontinuation of antidiabetic medication.	26](#_Toc236735165)

[Supplementary table 15. Distribution of ADHD diagnosis and ADHD medication records by country and age group among individuals identified with ADHD.	27](#_Toc236735166)

[Supplementary table 16. Sensitivity analyses on ADHD ascertainment source: association between ADHD and poor adherence to antidiabetic medication.	28](#_Toc236735167)

[Supplementary table 17. Sensitivity analyses on ADHD ascertainment source: association between ADHD and first discontinuation of antidiabetic medication.	31](#_Toc236735168)

[Supplementary table 18. Sensitivity analyses using country-specific prescription length and grace period: association between ADHD and poor adherence to antidiabetic medication.	33](#_Toc236735169)

[Supplementary table 19. Sensitivity analyses using country-specific prescription length and grace period: association between ADHD and first discontinuation of antidiabetic medication.	34](#_Toc236735170)

[Supplementary table 20. Sensitivity analyses using country-specific prescription length and grace period: association between ADHD medication and poor adherence to antidiabetic medication.	35](#_Toc236735171)

[Supplementary table 21. Age-stratified baseline characteristics.	36](#_Toc236735172)

[Supplementary table 22. Sensitivity analyses among individuals with confirmed type 2 diabetes diagnosis.	37](#_Toc236735173)

[Supplementary table 23. E-values for the associations of ADHD and ADHD medication use with antidiabetic medication discontinuation and poor adherence.	39](#_Toc236735174)

[Supplementary table 24. Post-hoc analyses of age-by-sex stratified analyses within the Australian cohort.	41](#_Toc236735175)

[Supplementary table 25. Country-specific p values for interaction terms in analyses examining the association of ADHD with antidiabetic medication discontinuation and poor adherence.	42](#_Toc236735176)

[Supplementary Figure 1. Schematic illustration of time-varying ADHD medication exposure classification based on dispensation or prescription data.	43](#_Toc236735177)

[Supplementary Figure 2. Schematic illustration of first discontinuation of any antidiabetic medication within five years of baseline.	44](#_Toc236735178)

[Supplementary Figure 3. Pooled and country-specific association between ADHD and poor adherence to antidiabetic medication in the exploratory model.	45](#_Toc236735179)

[Supplementary Figure 4. Pooled and country-specific association between ADHD medication and poor adherence to antidiabetic medication in the exploratory model.	46](#_Toc236735180)

[Supplementary Figure 5. Unadjusted cumulative incidence of the first antidiabetic medication discontinuation across seven countries.	47](#_Toc236735181)

[Supplementary Figure 6. Pooled and country-specific association between ADHD and the first discontinuation of antidiabetic medication in the exploratory model.	48](#_Toc236735182)

[Supplementary Figure 7. Pooled association between ADHD and poor adherence to antidiabetic medication with and without Australia.	49](#_Toc236735183)

[Supplementary Figure 8. Pooled association between ADHD medication and poor adherence to antidiabetic medication with and without US.	50](#_Toc236735184)

# Supplemental methods

## Data sources

Data sources included: national registers, administrative data, insurance claims or electronic health records (EHRs) with nationwide coverage (Denmark, Netherlands, Norway, Sweden); linked primary care and hospital EHR data representing ~6% of the UK population (The Health Improvement Network [THIN]);^1^ linked administrative and register data for Medicare-eligible adults residing in the state of New South Wales, Australia (MedIntel Data Platform);^2^ and federated EHR data from the US (TriNetX Research Network database, accessed on September 20, 2024).^3^ TriNetX included both insured and uninsured patients, covering over 60 healthcare organizations (hospitals, primary care and specialist providers).

Medication identification primarily used WHO Anatomical Therapeutic Chemical (ATC) codes from dispensation records (Australia, Denmark, Netherlands, Norway, Sweden) or equivalent drug codes mapped to ATC codes from prescription records (the UK, the US).^4^

Psychiatric diagnoses were identified using International Classification of Diseases, 10th Revision (ICD-10) codes, ICD-9 codes, ICD-8 codes or equivalent database-specific codes. The sources of diagnostic information and coverage period for psychiatric comorbidities varied by site: specialist diagnoses (Denmark 1969–2020, Netherlands 2011–2020, Norway 2008–2019, Sweden 1987–2020), combined primary care, hospital and specialist diagnoses (the UK 2001–2020, the US 1979–2020), and hospital inpatient diagnoses (Australia 2002–2020).

The four databases (Nordic and Dutch registers) provide nationwide coverage; the Australian data cover adults in New South Wales; the Health Improvement Network (THIN) represent ~6% of the UK population and is generalisable to the UK for demographics, major condition prevalence and death rates adjusted for demographics and deprivation; and the US database TriNetX, which covers over 60 healthcare organizations, includes a large routine-care population in USA.

**Reference**

1	McCarthy S, Wilton L, Murray ML, Hodgkins P, Asherson P, Wong ICK. Persistence of pharmacological treatment into adulthood, in UK primary care, for ADHD patients who started treatment in childhood or adolescence. *BMC Psychiatry* 2012; **12**: 219.

2	Zoega H, Falster MO, Gillies MB, *et al.* The Medicines Intelligence Data Platform: A Population-Based Data Resource From New South Wales, Australia. *Pharmacoepidemiology and Drug Safety* 2024; **33**: e5887.

3	Palchuk MB, London JW, Perez-Rey D, *et al.* A global federated real-world data and analytics platform for research. *JAMIA Open* 2023; **6**: ooad035.

4	Brikell I, Yao H, Li L, *et al.* ADHD medication discontinuation and persistence across the lifespan: a retrospective observational study using population-based databases. *Lancet Psychiatry* 2024; **11**: 16–26.

# Supplemental note 1. Ethical review and data sharing summary.

| **Country/region** | **Ethical Review Oversight** | **Data sharing statement** |
| --- | --- | --- |
| **Australia** | This research received ethical approval from the AIHW Human Research Ethics Committee (AIHW HREC) (approval number EO2021/1/1233) and the NSW Population and Health Services Research Ethics Committee (PHSREC) (approval number 2020/ETH02273). | Direct access to the data and analytical files to other individuals or authorities is not permitted without the express permission of the approving human research ethics committees and data custodians. |
| **Denmark** | The study was approved by the Danish Data Protection Agency, and data access was agreed upon by Statistics Denmark and the Danish Health Data Authority. Approval by the Ethics Committee and written informed consent were not required for register-based projects (cf. LBK nr 1338 af 01/09/2020, Act no. 1338 of 1 September 2020, section 10 on research ethics for administration of health scientific research projects and health data scientific research projects). | The data supporting the findings of this study are available from Statistics Denmark. Data access required the completion of a detailed application form from the Danish Data Protection Agency, the Danish National Board of Health, and Statistics Denmark. Researchers at Danish research institutions may obtain the relevant approval and data. International researchers may gain data access if governed by a Danish research institution having needed approval and data access. For more information on accessing the data, see http://www.dst.dk. |
| **Netherlands** | Analyses of administrative health records are exempt from the requirement of informed consent. Individual-level data were anonymized and did not leave the Statistics Netherlands server. | This study was performed under project agreement 9205 microdata services research. Individual-level data are prohibited from sharing or being publicly available. Data can be obtained by application to Statistics Netherlands. |
| **Norway** | The study was approved by the Regional Ethics Committee in Western Norway (2020/75421). | National regulations and laws prohibit sharing or making individual-level data publicly available. The data can be obtained by application to the respective registries and one of the Norwegian Regional Committees  for Medical and Health Research Ethics. |
| **Sweden** | This study was approved by the Swedish Ethical Review Authority (2020-06540). Informed consent is not required for pseudo-anonymized register-based research according to Swedish law. | The Public Access to Information and Secrecy Act in Sweden prohibits making individual-level data publicly available. Researchers interested in replicating this work can apply for individual-level data through Statistics Sweden at: https://www.scb.se/en/services/guidance-for-researchers-and-universities/. |
| **The United Kingdom** | The study was approved by the Health Improvement Network Scientific Review Committee (17THIN102). | Individual-level data are prohibited from sharing or being publicly available. Data can be obtained by application to the Health Improvement Network. |
| **The United States** | The study was approved by the Institutional Review Board of the SUNY Upstate Medical University (1933232-2). | The individual-level electronic health record data in the TriNetX database cannot be shared, as they are the property of TriNetX and can only be accessed through a direct contract with the company. |

# Supplementary table 1. Data source information by country/region.

| Selection criteria | **Australia** | **Denmark** | **Netherlands** | **Norway** | **Sweden** | **UK** | **US** |
| --- | --- | --- | --- | --- | --- | --- | --- |
| Death information | ✓ | ✓ | ✓ | ✓ | ✓ | ✓ | ✓ |
| Emigration information | X | ✓ | ✓ | ✓ | ✓ | ✓* | ✓** |
| Clinical diagnosis of type 1 diabetes | X | ✓ | X | ✓ | ✓ | ✓ | ✓ |

Note: √ = Required data available; X = Required data unavailable, * De-registration in the primary practice, **Last record in the participating healthcare organizations covered by TriNetX.

# Supplementary table 2. Definitions of covariates.

| Psychiatric Comorbidities | ICD-8 Codes | ICD-9 Codes | ICD-10 Codes |
| --- | --- | --- | --- |
| Anxiety disorders | 300.0 | 300.00,300.02 | F40-F41 |
| Autism spectrum disorder | 299.00, 299.01, 299.02, 299.03 | 299 | F84 |
| Bipolar disorder | 296.1, 296.3, 296.8 | 296A/ C/D/E/W | F30-F31 |
| Depressive disorder | 296.2, 298.0,300.4 | 296B,300E | F32-33 |
| Eating disorders | 306.5x | 307.5 | F50 |
| Intellectual disability | 310-315 | 317-319 | F70-F79 |
| Personality disorders | 301 | 301 | F60, F69 |
| Schizophrenia | 295 excl. 295.7 | 295A-E/G/W/X | F20-F29 |
| Substance use disorders | 291,303,304 | 291,292,304,305A,305X | F10-16, F18-F19 |

In the Netherlands, using mapping equivalents Netherlands Statistics codes; In the UK, using mapping equivalents READ codes.

# Supplementary table 3. Country-specific deviations from pooled estimates after Bonferroni correction.

| **Model** | **Year** | **Subgroup** | **Netherlands** | **Sweden** | **Australia** | **Denmark** | **Norway** | **United Kingdom** | **United States** |
| --- | --- | --- | --- | --- | --- | --- | --- | --- | --- |
| ADHD and poor adherence to antidiabetic medication | | | | | | | | | |
| Main model | 1 | Total population | TRUE |  | TRUE |  |  |  |  |
| Main model | 1 | Young adults (18-34 years) |  |  |  |  |  |  |  |
| Main model | 1 | Middle-aged adults (35-64 years) | TRUE |  |  |  |  |  |  |
| Main model | 1 | Older adults (≥65 years) |  |  |  |  |  |  |  |
| Main model | 1 | Female | TRUE |  | TRUE |  |  |  |  |
| Main model | 1 | Male |  |  |  |  |  |  |  |
| Exploratory model | 1 | Total population | TRUE |  | TRUE |  |  |  |  |
| Exploratory model | 1 | Young adults (18-34 years) |  |  |  |  |  |  |  |
| Exploratory model | 1 | Middle-aged adults (35-64 years) | TRUE |  |  |  |  |  |  |
| Exploratory model | 1 | Older adults (≥65 years) |  |  |  |  |  |  |  |
| Exploratory model | 1 | Female | TRUE |  | TRUE |  |  |  |  |
| Exploratory model | 1 | Male |  |  |  |  |  |  | TRUE |
| Main model | 2 | Total population | TRUE |  | TRUE |  |  |  |  |
| Main model | 2 | Young adults (18-34 years) |  |  |  |  |  |  | TRUE |
| Main model | 2 | Middle-aged adults (35-64 years) | TRUE |  |  |  |  |  |  |
| Main model | 2 | Older adults (≥65 years) |  |  |  |  |  |  |  |
| Main model | 2 | Female | TRUE |  | TRUE |  |  |  |  |
| Main model | 2 | Male | TRUE |  |  |  |  |  |  |
| Exploratory model | 2 | Total population | TRUE |  | TRUE |  |  |  |  |
| Exploratory model | 2 | Young adults (18-34 years) |  |  |  |  |  |  |  |
| Exploratory model | 2 | Middle-aged adults (35-64 years) |  |  |  |  |  |  |  |
| Exploratory model | 2 | Older adults (≥65 years) |  |  |  |  |  |  |  |
| Exploratory model | 2 | Female | TRUE |  | TRUE |  |  |  |  |
| Exploratory model | 2 | Male |  |  |  |  |  |  |  |
| Main model | 5 | Total population | TRUE |  | TRUE |  |  |  |  |
| Main model | 5 | Young adults (18-34 years) |  |  |  |  |  |  | TRUE |
| Main model | 5 | Middle-aged adults (35-64 years) |  |  |  |  |  |  |  |
| Main model | 5 | Older adults (≥65 years) |  | TRUE |  |  |  |  |  |
| Main model | 5 | Female | TRUE |  | TRUE |  |  |  |  |
| Main model | 5 | Male |  |  |  |  |  |  |  |
| Exploratory model | 5 | Total population | TRUE |  | TRUE |  |  |  |  |
| Exploratory model | 5 | Young adults (18-34 years) |  |  |  |  |  |  | TRUE |
| Exploratory model | 5 | Middle-aged adults (35-64 years) |  |  |  |  |  |  |  |
| Exploratory model | 5 | Older adults (≥65 years) |  | TRUE |  |  |  |  |  |
| Exploratory model | 5 | Female | TRUE |  | TRUE |  |  |  |  |
| Exploratory model | 5 | Male |  |  |  |  |  |  |  |
| ADHD medication and poor adherence to antidiabetic medication | | | | | | | | | |
| Main model | 1 | Total population |  |  |  |  |  |  | TRUE |
| Main model | 1 | Young adults (18-34 years) |  |  |  |  |  |  | TRUE |
| Main model | 1 | Middle-aged adults (35-64 years) |  |  |  |  |  |  | TRUE |
| Main model | 1 | Older adults (≥65 years) |  |  |  |  |  |  |  |
| Main model | 1 | Female |  |  |  |  |  |  | TRUE |
| Main model | 1 | Male |  |  |  |  |  |  | TRUE |
| Exploratory model | 1 | Total population |  |  |  |  |  |  | TRUE |
| Exploratory model | 1 | Young adults (18-34 years) |  |  |  |  |  |  | TRUE |
| Exploratory model | 1 | Middle-aged adults (35-64 years) |  |  |  |  |  |  | TRUE |
| Exploratory model | 1 | Older adults (≥65 years) |  |  |  |  |  |  |  |
| Exploratory model | 1 | Female |  |  |  |  |  |  | TRUE |
| Exploratory model | 1 | Male |  |  |  |  |  |  | TRUE |
| Main model | 2 | Total population |  |  |  |  |  |  | TRUE |
| Main model | 2 | Young adults (18-34 years) |  |  |  |  |  |  | TRUE |
| Main model | 2 | Middle-aged adults (35-64 years) |  |  |  |  |  |  | TRUE |
| Main model | 2 | Older adults (≥65 years) |  |  |  |  |  |  |  |
| Main model | 2 | Female |  |  |  |  |  |  | TRUE |
| Main model | 2 | Male |  |  |  |  |  |  | TRUE |
| Exploratory model | 2 | Total population |  |  |  |  |  |  | TRUE |
| Exploratory model | 2 | Young adults (18-34 years) |  |  |  |  |  |  | TRUE |
| Exploratory model | 2 | Middle-aged adults (35-64 years) |  |  |  |  |  |  | TRUE |
| Exploratory model | 2 | Older adults (≥65 years) |  |  |  |  |  |  |  |
| Exploratory model | 2 | Female |  |  |  |  |  |  | TRUE |
| Exploratory model | 2 | Male |  |  |  |  |  |  | TRUE |
| Main model | 5 | Total population |  |  |  |  |  |  | TRUE |
| Main model | 5 | Young adults (18-34 years) |  |  |  |  |  |  | TRUE |
| Main model | 5 | Middle-aged adults (35-64 years) |  |  |  |  |  |  | TRUE |
| Main model | 5 | Older adults (≥65 years) |  |  |  |  |  |  |  |
| Main model | 5 | Female |  |  |  |  |  |  | TRUE |
| Main model | 5 | Male |  |  |  |  |  |  | TRUE |
| Exploratory model | 5 | Total population |  |  |  |  |  |  | TRUE |
| Exploratory model | 5 | Young adults (18-34 years) |  |  |  |  |  |  | TRUE |
| Exploratory model | 5 | Middle-aged adults (35-64 years) |  |  |  |  |  |  | TRUE |
| Exploratory model | 5 | Older adults (≥65 years) |  |  |  |  |  |  |  |
| Exploratory model | 5 | Female |  |  |  |  |  |  | TRUE |
| Exploratory model | 5 | Male |  |  |  |  |  |  | TRUE |
| ADHD and first discontinuation of antidiabetic medication | | | | | | | | | |
| Main model | 5 | Total population | TRUE |  |  |  |  |  | TRUE |
| Main model | 5 | Young adults (18-34 years) |  |  |  |  |  |  |  |
| Main model | 5 | Middle-aged adults (35-64 years) |  |  |  |  |  |  | TRUE |
| Main model | 5 | Older adults (≥65 years) |  |  |  |  |  |  | TRUE |
| Main model | 5 | Female | TRUE |  |  |  |  |  |  |
| Main model | 5 | Male |  |  |  |  |  |  | TRUE |
| Exploratory model | 5 | Total population | TRUE |  |  |  |  |  | TRUE |
| Exploratory model | 5 | Young adults (18-34 years) |  |  |  |  |  |  |  |
| Exploratory model | 5 | Middle-aged adults (35-64 years) |  |  |  |  |  |  | TRUE |
| Exploratory model | 5 | Older adults (≥65 years) |  |  |  |  |  |  | TRUE |
| Exploratory model | 5 | Female | TRUE |  |  |  |  |  | TRUE |
| Exploratory model | 5 | Male |  |  |  |  |  |  | TRUE |

TRUE: significant; Main model, adjusted for sex, age and calendar year at initiation; Exploratory model, additionally adjusted for psychiatric comorbidities. Bonferroni correction for seven tests.

# Supplementary table 4. Exclusion criteria by country/region.

| **Selection criteria** | **Australia** | **Denmark** | **Netherlands** | **Norway** | **Sweden** | **United Kingdom** | **United States** |
| --- | --- | --- | --- | --- | --- | --- | --- |
| All individuals who used any antidiabetic medication (non-insulin) between 2010 to 2020 | 773655 | 370580 | 1170887 | 35086 | 652933 | 430262 | 3224044 |
| After excluding individuals filling any antidiabetic drug (including insulin) prescription before baseline* | 433385 | 225800 | 569272 | 27771 | 386171 | 238237 | 2798193 |
| After excluding individuals aged younger than 18 years at baseline | 427680 | 225110 | 567881 | 27320 | 384343 | 237256 | 2789020 |
| After excluding individuals with type 1 diabetes before or at baseline | - | 215720 | - | 25408 | 370065 | 234690 | 2016897 |
| After excluding individuals who started antidiabetic medication on December 31, 2020 | 427437 | 215700 | 567767 | 25408 | 370039 | 234647 | 2016004 |
| After excluding individuals who died before or at baseline** | 426979 | 215700 | 567739 | 25405 | 369996 | 234609 | 2009761 |
| After excluding individuals who emigrated before or at baseline** | 426979 | 215260 | 538663 | 24219 | 353030 | 234605 | 1850451 |
| After excluding individuals with missing values in sex or the birth year and month | 426978 | 215260 | 538663 | 24219 | 353030 | 234596 | 1850451 |
| Individuals who met the eligibility criteria | 426978 | 215260 | 538663 | 24219 | 353030 | 234596 | 1850451 |

Exclusion depended on the data available in each country/region, as reported in supplementary table 1.

*Baseline was this first eligible non-insulin antidiabetic medication dispensation.

**Records of emigration prior to antidiabetic medication dispensation can occur when a person emigrates but is still covered by a country’s healthcare system or when emigration is followed by immigration. Records of death prior to medication dispensation were rare and likely reflect entry errors in the databases.

- Clinical diagnoses of type 1 diabetes were not available.

# Supplementary table 5. Baseline characteristics of the pooled population, stratified by ADHD.

| **Baseline characteristics** | **Overall population** | **Total ADHD^+^** | **Total Non-ADHD^+^** |
| --- | --- | --- | --- |
| **N** | **3,643,197** | **88,339** | **3,554,858** |
| ADHD diagnosis^++^, n (%) | – | 39,441 (44.6) | – |
| ADHD medication^++^, n (%) | – | 76,973 (87.1) | – |
| Age at baseline, years, median range across countries | 38–63 | 32–51 | 38–63 |
| **Age, years, n (%)** |  |  |  |
| Young adults (18–34) | 337,076 (9.3) | 26,694 (30.2) | 310,382 (8.7) |
| Middle-aged adults (35–64) | 1,991,300 (54.7) | 52,184 (59.1) | 1,939,116 (54.5) |
| Older adults (≥65) | 1,314,821 (36.1) | 9,461 (10.7) | 1,305,360 (36.7) |
| Sex, female, n (%) | 1,871,870 (51.4) | 55,501 (62.8) | 1,816,369 (51.1) |
| Time to end of follow-up, days, median range across countries | 667–2,241 | 848–1,886 | 661–2,242 |
| At least one psychiatric comorbidity, n (%) | 538,675 (14.8) | 36,331 (41.1) | 502,344 (14.1) |
| Anxiety disorder, n (%) | 244,359 (6.7) | 23,962 (27.1) | 220,397 (6.2) |
| Autism spectrum disorders, n (%) | 4,481 (0.1) | 1,391 (1.6) | 3,090 (0.1) |
| Bipolar disorder, n (%) | 43,232 (1.2) | 6,570 (7.4) | 36,662 (1.0) |
| Intellectual disability, n (%) | 11,487 (0.3) | 1,479 (1.7) | 10,008 (0.3) |
| Depressive disorder, n (%) | 142,089 (3.9) | 8,330 (9.4) | 133,759 (3.8) |
| Schizophrenia, n (%) | 55,803 (1.5) | 4,266 (4.8) | 51,537 (1.4) |
| Personality disorder, n (%) | 37,091 (1.0) | 5,748 (6.5) | 31,343 (0.9) |
| Eating disorders, n (%) | 13,644 (0.4) | 2,367 (2.7) | 11,277 (0.3) |
| Substance use disorder, n (%) | 213,356 (5.9) | 13,029 (14.7) | 200,327 (5.6) |

^+^Lifetime ADHD; ^++^ recorded ADHD diagnosis or at least one ADHD medication before the end of the study period; – not applicable.

# Supplementary table 6. Median (25th percentile, 75th percentile) proportion of days covered (PDC) for antidiabetic medication by subgroups across seven countries.

**A 1-year follow up**

| **Country** | **Subgroup** | **Analysis of ADHD and poor adherence to antidiabetic medication** | | **Analysis of ADHD medication and poor adherence to antidiabetic medication** | |
| --- | --- | --- | --- | --- | --- |
|  |  | **Without ADHD** | **With ADHD** | **Without ADHD medication** | **With ADHD medication** |
| Australia | Total population | 0.57 (0.28–0.90) | 0.56 (0.25–0.93) | 0.56 (0.27–0.98) | 0.73 (0.37–1.00) |
|  | Young adults (18-34 years) | 0.38 (0.19–0.74) | 0.54 (0.19–0.92) | 0.54 (0.21–0.95) | 0.72 (0.35–1.00) |
|  | Middle-aged adults (35-64 years) | 0.56 (0.28–0.90) | 0.56 (0.28–0.95) | 0.58 (0.28–0.99) | 0.74 (0.38–1.00) |
|  | Older adults (≥65 years) | 0.63 (0.45–0.95) | 0.62 (0.45–0.92) | 0.63 (0.45–1.00) | 0.69 (0.50–1.00) |
|  | Female | 0.51 (0.19–0.88) | 0.55 (0.20–0.93) | 0.55 (0.23–0.95) | 0.73 (0.37–1.00) |
|  | Male | 0.60 (0.31–0.94) | 0.56 (0.28–0.93) | 0.59 (0.28–0.99) | 0.74 (0.39–1.00) |
| Denmark | Total population | 0.91 (0.67–1.00) | 0.83 (0.40–1.00) | 0.85 (0.43–1.00) | 0.97 (0.69–1.00) |
|  | Young adults (18-34 years) | 0.52 (0.31–0.94) | 0.69 (0.31–1.00) | 0.58 (0.25–1.00) | 0.82 (0.45–1.00) |
|  | Middle-aged adults (35-64 years) | 0.92 (0.67–1.00) | 0.86 (0.52–1.00) | 0.93 (0.56–1.00) | 1.00 (0.72–1.00) |
|  | Older adults (≥65 years) | 0.95 (0.75–1.00) | 0.98 (0.58–1.00) | - | - |
|  | Female | 0.86 (0.57–1.00) | 0.75 (0.35–1.00) | 0.74 (0.35–1.00) | 0.93 (0.56–1.00) |
|  | Male | 0.95 (0.69–1.00) | 0.86 (0.55–1.00) | 0.92 (0.55–1.00) | 1.00 (0.75–1.00) |
| Netherlands | Total population | 1.00 (0.64–1.00) | 1.00 (0.70–1.00) | 1.00 (0.87–1.00) | 1.00 (1.00–1.00) |
|  | Young adults (18-34 years) | 0.85 (0.46–1.00) | 0.94 (0.49–1.00) | 1.00 (0.54–1.00) | 1.00 (0.70–1.00) |
|  | Middle-aged adults (35-64 years) | 1.00 (1.00–1.00) | 1.00 (0.90–1.00) | 1.00 (1.00–1.00) | 1.00 (1.00–1.00) |
|  | Older adults (≥65 years) | 0.81 (0.44–1.00) | 0.86 (0.44–1.00) | 0.88 (0.46–1.00) | 1.00 (0.88–1.00) |
|  | Female | 0.90 (0.44–1.00) | 1.00 (0.48–1.00) | 1.00 (0.60–1.00) | 1.00 (0.95–1.00) |
|  | Male | 1.00 (0.89–1.00) | 1.00 (0.88–1.00) | 1.00 (1.00–1.00) | 1.00 (1.00–1.00) |
| Norway | Total population | 0.84 (0.50–1.00) | 0.75 (0.41–1.00) | 0.90 (0.50–1.00) | 1.00 (0.50–1.00) |
|  | Young adults (18-34 years) | 0.58 (0.25–1.00) | 0.67 (0.25–1.00) | 0.75 (0.42–1.00) | 0.75 (0.42–1.00) |
|  | Middle-aged adults (35-64 years) | 0.99 (0.59–1.00) | 1.00 (0.50–1.00) | 1.00 (0.67–1.00) | 1.00 (0.76–1.00) |
|  | Female | 0.75 (0.29–1.00) | 0.66 (0.25–1.00) | 0.75 (0.39–1.00) | 0.85 (0.50–1.00) |
|  | Male | 1.00 (0.75–1.00) | 1.00 (0.67–1.00) | 1.00 (0.75–1.00) | 1.00 (0.84–1.00) |
| Sweden | Total population | 0.93 (0.71–1.00) | 0.90 (0.49–1.00) | 0.92 (0.53–1.00) | 1.00 (0.70–1.00) |
|  | Young adults (18-34 years) | 0.52 (0.19–0.84) | 0.62 (0.32–1.00) | 0.70 (0.35–1.00) | 0.90 (0.49–1.00) |
|  | Middle-aged adults (35-64 years) | 0.94 (0.72–1.00) | 0.96 (0.68–1.00) | 1.00 (0.72–1.00) | 1.00 (0.82–1.00) |
|  | Older adults (≥65 years) | 0.94 (0.75–1.00) | 0.98 (0.61–1.00) | 1.00 (0.73–1.00) | 1.00 (0.62–1.00) |
|  | Female | 0.91 (0.66–1.00) | 0.81 (0.36–1.00) | 0.85 (0.41–1.00) | 1.00 (0.61–1.00) |
|  | Male | 0.96 (0.75–1.00) | 0.95 (0.61–1.00) | 0.97 (0.71–1.00) | 1.00 (0.76–1.00) |
| United Kingdom | Total population | 0.90 (0.55–1.00) | 0.84 (0.41–1.00) | 0.84 (0.41–1.00) | 1.00 (0.86–1.00) |
|  | Young adults (18-34 years) | 0.41 (0.23–0.88) | 0.71 (0.27–1.00) | 0.76 (0.27–1.00) | 1.00 (0.52–1.00) |
|  | Middle-aged adults (35-64 years) | 0.95 (0.64–1.00) | 0.96 (0.58–1.00) | 0.99 (0.60–1.00) | 1.00 (0.91–1.00) |
|  | Older adults (≥65 years) | 0.88 (0.58–1.00) | 0.83 (0.52–1.00) | 0.69 (0.30–1.00) | 1.00 (1.00–1.00) |
|  | Female | 0.81 (0.41–1.00) | 0.68 (0.27–1.00) | 0.58 (0.25–1.00) | 1.00 (0.72–1.00) |
|  | Male | 0.96 (0.66–1.00) | 0.97 (0.60–1.00) | 1.00 (0.64–1.00) | 1.00 (0.92–1.00) |
| United States | Total population | 0.40 (0.17–0.79) | 0.38 (0.15–0.78) | 0.30 (0.15–0.68) | 0.99 (0.56–1.00) |
|  | Young adults (18-34 years) | 0.27 (0.14–0.62) | 0.29 (0.14–0.68) | 0.27 (0.14–0.58) | 0.73 (0.46–1.00) |
|  | Middle-aged adults (35-64 years) | 0.42 (0.15–0.78) | 0.43 (0.15–0.82) | 0.31 (0.15–0.73) | 1.00 (0.58–1.00) |
|  | Older adults (≥65 years) | 0.42 (0.17–0.83) | 0.40 (0.17–0.84) | 0.33 (0.17–0.70) | 1.00 (0.74–1.00) |
|  | Female | 0.38 (0.17–0.77) | 0.36 (0.15–0.75) | 0.30 (0.15–0.67) | 0.89 (0.51–1.00) |
|  | Male | 0.43 (0.16–0.81) | 0.42 (0.16–0.83) | 0.33 (0.15–0.72) | 1.00 (0.64–1.00) |

**B 2-year follow up**

| **Country** | **Subgroup** | **Analysis of ADHD and poor adherence to antidiabetic medication** | | **Analysis of ADHD medication and poor adherence to antidiabetic medication** | |
| --- | --- | --- | --- | --- | --- |
|  |  | **Without ADHD** | **With ADHD** | **Without ADHD medication** | **With ADHD medication** |
| Australia | Total population | 0.49 (0.19–0.85) | 0.44 (0.16–0.85) | 0.47 (0.19–0.94) | 0.65 (0.28–1.00) |
|  | Young adults (18-34 years) | 0.27 (0.09–0.57) | 0.38 (0.15–0.78) | 0.41 (0.17–0.88) | 0.64 (0.28–1.00) |
|  | Middle-aged adults (35-64 years) | 0.51 (0.21–0.86) | 0.46 (0.15–0.88) | 0.52 (0.20–0.98) | 0.68 (0.30–1.00) |
|  | Older adults (≥65 years) | 0.60 (0.31–0.92) | 0.57 (0.37–0.91) | 0.61 (0.40–0.98) | 0.66 (0.42–1.00) |
|  | Female | 0.44 (0.15–0.79) | 0.40 (0.15–0.82) | 0.44 (0.17–0.90) | 0.64 (0.28–1.00) |
|  | Male | 0.56 (0.28–0.90) | 0.47 (0.16–0.88) | 0.54 (0.21–0.98) | 0.67 (0.31–1.00) |
| Denmark | Total population | 0.82 (0.52–1.00) | 0.69 (0.26–1.00) | 0.76 (0.32–1.00) | 0.91 (0.54–1.00) |
|  | Young adults (18-34 years) | 0.38 (0.17–0.78) | 0.48 (0.17–0.89) | 0.47 (0.17–0.96) | 0.74 (0.34–1.00) |
|  | Middle-aged adults (35-64 years) | 0.83 (0.55–1.00) | 0.77 (0.35–1.00) | 0.86 (0.48–1.00) | 0.97 (0.65–1.00) |
|  | Older adults (≥65 years) | 0.85 (0.66–1.00) | 0.83 (0.23–1.00) | - | - |
|  | Female | 0.77 (0.43–1.00) | 0.60 (0.17–0.96) | 0.65 (0.24–1.00) | 0.86 (0.43–1.00) |
|  | Male | 0.86 (0.60–1.00) | 0.78 (0.37–1.00) | 0.83 (0.47–1.00) | 0.97 (0.68–1.00) |
| Netherlands | Total population | 0.91 (0.45–1.00) | 0.95 (0.45–1.00) | 1.00 (0.70–1.00) | 1.00 (0.94–1.00) |
|  | Young adults (18-34 years) | 0.68 (0.25–1.00) | 0.70 (0.26–1.00) | 0.87 (0.40–1.00) | 1.00 (0.60–1.00) |
|  | Middle-aged adults (35-64 years) | 1.00 (0.82–1.00) | 1.00 (0.61–1.00) | 1.00 (0.90–1.00) | 1.00 (1.00–1.00) |
|  | Older adults (≥65 years) | 0.67 (0.36–0.94) | 0.66 (0.26–0.99) | 0.79 (0.40–1.00) | 1.00 (0.75–1.00) |
|  | Female | 0.71 (0.37–1.00) | 0.84 (0.34–1.00) | 1.00 (0.46–1.00) | 1.00 (0.80–1.00) |
|  | Male | 0.97 (0.74–1.00) | 1.00 (0.58–1.00) | 1.00 (0.84–1.00) | 1.00 (1.00–1.00) |
| Norway | Total population | 0.66 (0.25–1.00) | 0.62 (0.25–1.00) | 0.78 (0.32–1.00) | 0.94 (0.38–1.00) |
|  | Young adults (18-34 years) | 0.37 (0.18–0.81) | 0.46 (0.15–0.87) | 0.57 (0.25–1.00) | 0.70 (0.29–1.00) |
|  | Middle-aged adults (35-64 years) | 0.84 (0.38–1.00) | 0.84 (0.35–1.00) | 1.00 (0.56–1.00) | 1.00 (0.66–1.00) |
|  | Female | 0.50 (0.25–0.91) | 0.48 (0.16–0.89) | 0.57 (0.25–1.00) | 0.75 (0.30–1.00) |
|  | Male | 0.88 (0.50–1.00) | 0.87 (0.41–1.00) | 1.00 (0.71–1.00) | 1.00 (0.76–1.00) |
| Sweden | Total population | 0.87 (0.59–1.00) | 0.76 (0.31–1.00) | 0.86 (0.44–1.00) | 1.00 (0.62–1.00) |
|  | Young adults (18-34 years) | 0.36 (0.17–0.72) | 0.48 (0.18–0.86) | 0.57 (0.21–0.99) | 0.80 (0.38–1.00) |
|  | Middle-aged adults (35-64 years) | 0.88 (0.62–1.00) | 0.86 (0.46–1.00) | 0.95 (0.66–1.00) | 1.00 (0.75–1.00) |
|  | Older adults (≥65 years) | 0.89 (0.65–1.00) | 0.84 (0.39–1.00) | 0.97 (0.69–1.00) | 1.00 (0.59–1.00) |
|  | Female | 0.84 (0.49–0.99) | 0.64 (0.24–0.98) | 0.77 (0.29–1.00) | 0.95 (0.49–1.00) |
|  | Male | 0.90 (0.64–1.00) | 0.85 (0.45–1.00) | 0.94 (0.63–1.00) | 1.00 (0.74–1.00) |
| United Kingdom | Total population | 0.77 (0.34–1.00) | 0.66 (0.23–1.00) | 0.76 (0.27–1.00) | 1.00 (0.81–1.00) |
|  | Young adults (18-34 years) | 0.27 (0.14–0.68) | 0.48 (0.14–0.99) | 0.60 (0.20–1.00) | 1.00 (0.48–1.00) |
|  | Middle-aged adults (35-64 years) | 0.85 (0.46–1.00) | 0.77 (0.37–1.00) | 0.88 (0.42–1.00) | 1.00 (0.85–1.00) |
|  | Older adults (≥65 years) | 0.75 (0.38–1.00) | 0.81 (0.31–1.00) | 0.77 (0.25–1.00) | 1.00 (1.00–1.00) |
|  | Female | 0.68 (0.24–1.00) | 0.46 (0.14–0.97) | 0.48 (0.16–1.00) | 1.00 (0.56–1.00) |
|  | Male | 0.85 (0.48–1.00) | 0.78 (0.37–1.00) | 0.90 (0.47–1.00) | 1.00 (0.86–1.00) |
| United States | Total population | 0.30 (0.13–0.67) | 0.28 (0.11–0.64) | 0.23 (0.09–0.57) | 0.89 (0.49–1.00) |
|  | Young adults (18-34 years) | 0.21 (0.09–0.52) | 0.24 (0.11–0.55) | 0.20 (0.07–0.48) | 0.69 (0.36–1.00) |
|  | Middle-aged adults (35-64 years) | 0.30 (0.14–0.66) | 0.30 (0.12–0.67) | 0.25 (0.10–0.61) | 0.91 (0.50–1.00) |
|  | Older adults (≥65 years) | 0.32 (0.13–0.71) | 0.30 (0.09–0.71) | 0.25 (0.09–0.59) | 1.00 (0.72–1.00) |
|  | Female | 0.30 (0.13–0.66) | 0.27 (0.11–0.62) | 0.22 (0.08–0.54) | 0.81 (0.45–1.00) |
|  | Male | 0.31 (0.13–0.68) | 0.30 (0.12–0.69) | 0.25 (0.10–0.61) | 1.00 (0.57–1.00) |

**C 5-year follow up**

| **Country** | **Subgroup** | **Analysis of ADHD and poor adherence to antidiabetic medication** | | **Analysis of ADHD medication and poor adherence to antidiabetic medication** | |
| --- | --- | --- | --- | --- | --- |
|  |  | **Without ADHD** | **With ADHD** | **Without ADHD medication** | **With ADHD medication** |
| Australia | Total population | 0.40 (0.10–0.75) | 0.32 (0.09–0.72) | 0.44 (0.15–0.91) | 0.64 (0.24–1.00) |
|  | Young adults (18-34 years) | 0.18 (0.08–0.46) | 0.28 (0.09–0.67) | 0.36 (0.13–0.82) | 0.60 (0.22–1.00) |
|  | Middle-aged adults (35-64 years) | 0.43 (0.11–0.78) | 0.34 (0.08–0.75) | 0.50 (0.17–0.97) | 0.66 (0.26–1.00) |
|  | Older adults (≥65 years) | 0.52 (0.16–0.83) | 0.50 (0.19–0.82) | 0.61 (0.36–0.94) | 0.72 (0.42–1.00) |
|  | Female | 0.31 (0.08–0.69) | 0.30 (0.10–0.70) | 0.38 (0.14–0.86) | 0.59 (0.22–1.00) |
|  | Male | 0.49 (0.14–0.81) | 0.35 (0.08–0.73) | 0.52 (0.17–0.96) | 0.67 (0.28–1.00) |
| Denmark | Total population | 0.70 (0.30–0.94) | 0.48 (0.10–0.87) | 0.74 (0.28–1.00) | 0.89 (0.49–1.00) |
|  | Young adults (18-34 years) | 0.24 (0.08–0.59) | 0.31 (0.09–0.76) | 0.39 (0.11–0.89) | 0.70 (0.25–1.00) |
|  | Middle-aged adults (35-64 years) | 0.71 (0.35–0.96) | 0.57 (0.11–0.90) | 0.85 (0.46–1.00) | 0.93 (0.63–1.00) |
|  | Older adults (≥65 years) | 0.73 (0.40–0.95) | 0.61 (0.09–0.98) | - | - |
|  | Female | 0.66 (0.22–0.92) | 0.38 (0.09–0.83) | 0.62 (0.17–1.00) | 0.83 (0.33–1.00) |
|  | Male | 0.73 (0.39–0.96) | 0.59 (0.11–0.92) | 0.83 (0.44–1.00) | 0.95 (0.66–1.00) |
| Netherlands | Total population | 0.74 (0.30–0.99) | 0.66 (0.11–1.00) | 1.00 (0.61–1.00) | 1.00 (0.86–1.00) |
|  | Young adults (18-34 years) | 0.47 (0.11–0.87) | 0.40 (0.06–0.87) | 0.80 (0.30–1.00) | 0.99 (0.53–1.00) |
|  | Middle-aged adults (35-64 years) | 0.92 (0.45–1.00) | 0.79 (0.18–1.00) | 1.00 (0.81–1.00) | 1.00 (0.95–1.00) |
|  | Older adults (≥65 years) | 0.49 (0.23–0.82) | 0.40 (0.01–0.82) | 0.74 (0.37–1.00) | 1.00 (0.75–1.00) |
|  | Female | 0.51 (0.25–0.96) | 0.51 (0.09–0.96) | 0.93 (0.40–1.00) | 1.00 (0.71–1.00) |
|  | Male | 0.82 (0.41–1.00) | 0.75 (0.13–1.00) | 1.00 (0.78–1.00) | 1.00 (0.95–1.00) |
| Norway | Total population | 0.43 (0.13–0.86) | 0.39 (0.10–0.84) | 0.68 (0.24–1.00) | 0.92 (0.35–1.00) |
|  | Young adults (18-34 years) | 0.27 (0.10–0.63) | 0.30 (0.10–0.68) | 0.48 (0.17–0.99) | 0.67 (0.23–1.00) |
|  | Middle-aged adults (35-64 years) | 0.61 (0.16–0.92) | 0.53 (0.09–0.94) | 1.00 (0.50–1.00) | 1.00 (0.60–1.00) |
|  | Female | 0.31 (0.10–0.74) | 0.30 (0.10–0.70) | 0.49 (0.18–1.00) | 0.67 (0.23–1.00) |
|  | Male | 0.69 (0.22–0.95) | 0.56 (0.08–0.94) | 1.00 (0.60–1.00) | 1.00 (0.79–1.00) |
| Sweden | Total population | 0.79 (0.39–0.94) | 0.53 (0.11–0.91) | 0.85 (0.41–1.00) | 0.98 (0.58–1.00) |
|  | Young adults (18-34 years) | 0.26 (0.09–0.63) | 0.31 (0.08–0.73) | 0.53 (0.19–0.97) | 0.76 (0.33–1.00) |
|  | Middle-aged adults (35-64 years) | 0.80 (0.42–0.95) | 0.66 (0.15–0.95) | 0.94 (0.61–1.00) | 1.00 (0.75–1.00) |
|  | Older adults (≥65 years) | 0.81 (0.45–0.94) | 0.56 (0.10–0.90) | 0.95 (0.67–1.00) | 0.99 (0.59–1.00) |
|  | Female | 0.75 (0.29–0.92) | 0.43 (0.09–0.85) | 0.74 (0.25–1.00) | 0.92 (0.44–1.00) |
|  | Male | 0.82 (0.47–0.96) | 0.64 (0.15–0.94) | 0.92 (0.58–1.00) | 1.00 (0.73–1.00) |
| United Kingdom | Total population | 0.52 (0.12–0.90) | 0.36 (0.06–0.82) | 0.59 (0.14–1.00) | 1.00 (0.75–1.00) |
|  | Young adults (18-34 years) | 0.14 (0.05–0.43) | 0.21 (0.05–0.62) | 0.39 (0.12–1.00) | 0.96 (0.52–1.00) |
|  | Middle-aged adults (35-64 years) | 0.62 (0.17–0.96) | 0.42 (0.08–0.92) | 0.75 (0.24–1.00) | 1.00 (0.79–1.00) |
|  | Older adults (≥65 years) | 0.50 (0.12–0.86) | 0.56 (0.07–0.95) | 0.73 (0.11–0.98) | 1.00 (0.96–1.00) |
|  | Female | 0.41 (0.09–0.83) | 0.22 (0.05–0.74) | 0.27 (0.08–0.99) | 1.00 (0.53–1.00) |
|  | Male | 0.61 (0.17–0.95) | 0.47 (0.07–0.88) | 0.77 (0.33–1.00) | 1.00 (0.81–1.00) |
| United States | Total population | 0.24 (0.08–0.58) | 0.20 (0.06–0.51) | 0.19 (0.06–0.51) | 0.83 (0.44–1.00) |
|  | Young adults (18-34 years) | 0.18 (0.07–0.47) | 0.18 (0.07–0.46) | 0.15 (0.06–0.41) | 0.69 (0.33–1.00) |
|  | Middle-aged adults (35-64 years) | 0.25 (0.08–0.57) | 0.21 (0.06–0.53) | 0.21 (0.07–0.55) | 0.84 (0.46–1.00) |
|  | Older adults (≥65 years) | 0.26 (0.08–0.62) | 0.20 (0.04–0.56) | 0.20 (0.07–0.54) | 1.00 (0.68–1.00) |
|  | Female | 0.24 (0.08–0.56) | 0.19 (0.06–0.49) | 0.18 (0.06–0.48) | 0.74 (0.39–1.00) |
|  | Male | 0.25 (0.08–0.59) | 0.21 (0.06–0.54) | 0.21 (0.07–0.56) | 0.98 (0.51–1.00) |

Median Proportion of Days Covered (PDC) for antidiabetic medication (25% percentile, 75% percentile).

In analysis of ADHD medication and poor adherence to antidiabetic medication, study population restricted to individuals who received a clinical ADHD diagnosis or an ADHD medication before or at baseline.

# Supplementary table 7. Statistical test for between-country heterogeneity.

| **Subgroup** | **Cochran Q test p value (I², %)** | **Year** | **Analysis** |
| --- | --- | --- | --- |
| Total population | <0.001 (95.37) | 1 | ADHD and poor adherence |
| Young adults (18-34 years) | <0.001 (91.06) | 1 | ADHD and poor adherence |
| Middle-aged adults (35-64 years) | <0.001 (88.74) | 1 | ADHD and poor adherence |
| Older adults (≥65 years) | 0.092 (47.11) | 1 | ADHD and poor adherence |
| Female | <0.001 (93.38) | 1 | ADHD and poor adherence |
| Male | <0.001 (86.75) | 1 | ADHD and poor adherence |
| Total population | <0.001 (96.33) | 2 | ADHD and poor adherence |
| Young adults (18-34 years) | <0.001 (91.92) | 2 | ADHD and poor adherence |
| Middle-aged adults (35-64 years) | <0.001 (86.44) | 2 | ADHD and poor adherence |
| Older adults (≥65 years) | 0.032 (58.95) | 2 | ADHD and poor adherence |
| Female | <0.001 (94.23) | 2 | ADHD and poor adherence |
| Male | <0.001 (91.16) | 2 | ADHD and poor adherence |
| Total population | <0.001 (97.06) | 5 | ADHD and poor adherence |
| Young adults (18-34 years) | <0.001 (95.33) | 5 | ADHD and poor adherence |
| Middle-aged adults (35-64 years) | <0.001 (81.43) | 5 | ADHD and poor adherence |
| Older adults (≥65 years) | <0.001 (79.09) | 5 | ADHD and poor adherence |
| Female | <0.001 (96.38) | 5 | ADHD and poor adherence |
| Male | <0.001 (86.95) | 5 | ADHD and poor adherence |
| Total population | <0.001 (99.29) | 1 | ADHD medication and poor adherence |
| Young adults (18-34 years) | <0.001 (97.16) | 1 | ADHD medication and poor adherence |
| Middle-aged adults (35-64 years) | <0.001 (98.74) | 1 | ADHD medication and poor adherence |
| Older adults (≥65 years) | <0.001 (96.99) | 1 | ADHD medication and poor adherence |
| Female | <0.001 (98.40) | 1 | ADHD medication and poor adherence |
| Male | <0.001 (98.83) | 1 | ADHD medication and poor adherence |
| Total population | <0.001 (99.44) | 2 | ADHD medication and poor adherence |
| Young adults (18-34 years) | <0.001 (97.42) | 2 | ADHD medication and poor adherence |
| Middle-aged adults (35-64 years) | <0.001 (99.09) | 2 | ADHD medication and poor adherence |
| Older adults (≥65 years) | <0.001 (97.20) | 2 | ADHD medication and poor adherence |
| Female | <0.001 (98.80) | 2 | ADHD medication and poor adherence |
| Male | <0.001 (99.02) | 2 | ADHD medication and poor adherence |
| Total population | <0.001 (99.48) | 5 | ADHD medication and poor adherence |
| Young adults (18-34 years) | <0.001 (97.37) | 5 | ADHD medication and poor adherence |
| Middle-aged adults (35-64 years) | <0.001 (99.19) | 5 | ADHD medication and poor adherence |
| Older adults (≥65 years) | <0.001 (97.30) | 5 | ADHD medication and poor adherence |
| Female | <0.001 (98.85) | 5 | ADHD medication and poor adherence |
| Male | <0.001 (99.10) | 5 | ADHD medication and poor adherence |
| Total population | <0.001 (97.93) | 5 | ADHD and first discontinuation |
| Young adults (18-34 years) | <0.001 (88.65) | 5 | ADHD and first discontinuation |
| Middle-aged adults (35-64 years) | <0.001 (94.54) | 5 | ADHD and first discontinuation |
| Older adults (≥65 years) | <0.001 (92.62) | 5 | ADHD and first discontinuation |
| Female | <0.001 (96.62) | 5 | ADHD and first discontinuation |
| Male | <0.001 (96.62) | 5 | ADHD and first discontinuation |

We assessed between-country heterogeneity using Cochran’s Q test and I² statistics, with both a two-sided *P*<0.05 in Cochran's Q test and I²>75% indicating statistically significant heterogeneity.

# Supplementary table 8. Number of individuals included by subgroups.

| **Population** | **Subgroup** | **Analysis of ADHD and antidiabetic medication discontinuation and adherence** | | | **Analysis of ADHD medication and antidiabetic medication adherence** | | |
| --- | --- | --- | --- | --- | --- | --- | --- |
|  |  | **Total** | **ADHD^+^** | **Non-ADHD^+^** | **Total** | **ADHD medication*^+^** | **No ADHD medication*^+^** |
| Pooled from seven countries | Total population | **3,643,197** | 88,339 | 3,554,858 | 61,403 | 54,478 (88.7%) | 6,925 (11.3%) |
|  | Young adults (18-34 years) | 337,076 | 26,694 | 310,382 | 17,132 | 14,552 (84.9%) | 2,580 (15.1%) |
|  | Middle-aged adults (35-64 years) | 1,991,300 | 52,184 | 1,939,116 | 37,699 | 33,915 (90.0%) | 3,784 (10.0%) |
|  | Older adults (≥65 years) | 1,314,821 | 9,461 | 1,305,360 | 6,572 | 6,011 (91.5%) | 561 (8.5%) |
|  | Female | 1,871,870 | 55,501 | 1,816,369 | 36,749 | 33,081 (90.0%) | 3,668 (10.0%) |
|  | Male | 1,771,327 | 32,838 | 1,738,489 | 24,654 | 21,397 (86.8%) | 3,257 (13.2%) |

In analysis of ADHD medication and poor adherence to antidiabetic medication, study population restricted to individuals who had a diagnosis or received an ADHD medication at or before baseline.

**^+^**ADHD is defined as individuals had a recorded diagnosis or received an ADHD medication by the end of study period. ADHD medication is defined as individuals had received an ADHD medication by the end of study period, otherwise, no ADHD medication.

*Among individuals who had a diagnosis or received an ADHD medication at or before baseline, the percentage of individuals who received (never received) an ADHD medication by the end of study period.

# Supplementary table 9. Baseline characteristics among individuals identified with ADHD before baseline across seven countries.

| **Baseline characteristics** | **Australia ADHD medication** | | **Denmark ADHD medication** | | **Netherlands**  **ADHD medication** | | **Norway**  **ADHD medication** | | **Sweden**  **ADHD medication** | | **United Kingdom**  **ADHD medication** | | **United States**  **ADHD medication** | |
| --- | --- | --- | --- | --- | --- | --- | --- | --- | --- | --- | --- | --- | --- | --- |
|  | **Yes^+^** | **No^+^** | **Yes^+^** | **No^+^** | **Yes^+^** | **No^+^** | **Yes^+^** | **No^+^** | **Yes^+^** | **No^+^** | **Yes^+^** | **No^+^** | **Yes^+^** | **No^+^** |
| N | 3,186 | - | ~2,040 | ~130 | 5,424 | 403 | 784 | 120 | 3,208 | 613 | 172 | 226 | 39,664 | 5,433 |
| Age at baseline, years | 34 [24, 48] | - | 42 [30, 51] | 34 [27, 44] | 50 [42, 60] | 49 [41, 56] | 33 [26, 41] | 31 [25, 40] | 43 [30, 52] | 42 [32, 52] | 39 [26, 52] | 34 [26, 52] | 47 [35, 58] | 41 [29, 54] |
| Age, years, n(%) | | | | | | | | | | | | | | |
| Young adults (18-34) | 1,608 (50.5) | - | ~680 (33.3) | ~70 (53.8) | 793 (14.6) | 63 (15.6) | 426 (54.3) | 71 (59.2) | 1,053 (32.8) | 198 (32.3) | 76 (44.2) | 115 (50.9) | 9,916 (25.0) | 2,063 (38.0) |
| Middle-aged adults (35-64) | 1,453 (45.6) | - | ~1,360 (66.7) | ~60 (46.2) | 3,701 (68.2) | 307 (76.2) | 358 (45.7) | 49 (40.8) | 2,003 (62.4) | 382 (62.3) | 75 (43.6) | 86 (38.1) | 24,965 (62.9) | 2,900 (53.4) |
| Older adults (≥65) | 125 (3.9) | - |  |  | 930 (17.1) | 33 (8.2) | - | - | 152 (4.7) | 33 (5.4) | 21 (12.2) | 25 (11.1) | 4,783 (12.1) | 470 (8.7) |
| Sex, female, n(%) | 1,758 (55.2) | - | ~1,090 (53.4) | ~70 (53.8) | 2,409 (44.4) | 150 (37.2) | 498 (63.5) | 74 (61.7) | 1,619 (50.5) | 262 (42.7) | 78 (45.3) | 111 (49.1) | 25,629 (64.6) | 3,001 (55.2) |
| Time to end of follow-up, days | 937 [391, 1,681] | - | 1,060 [420, 2,050] | 1,080 [440, 1,810] | 1,350 [593, 2,381] | 864 [317, 1,578] | 1,021 [411, 1,964] | 791 [252, 1,599] | 1,028 [427, 1,890] | 1,112 [471, 1,984] | 1,688 [962, 2,713.75] | 1,738 [815, 2,723] | 1,116 [495, 1,914] | 978 [443, 1,723] |
| At least one psychiatric comorbidity, n(%) | 1,146 (36.0) | - | ~970 (47.5) | 100 (76.9) | 2,311 (42.6) | 320 (79.4) | 495 (63.1) | 85 (70.8) | 2,665 (83.1) | 531 (86.6) | 123 (71.5) | 151 (66.8) | 15,976 (40.3) | 3,602 (66.3) |
| Anxiety disorder, n(%) | 525 (16.5) | - | ~270 (13.2) | ~20 (15.4) | 732 (13.5) | 100 (24.8) | 217 (27.7) | 36 (30.0) | 1,703 (53.1) | 315 (51.4) | 23 (13.4) | 36 (15.9) | 12,310 (31.0) | 2,636 (48.5) |
| Autism spectrum disorders, n(%) | 106 (3.3) | - | ~80 (3.9) | ~10 (7.7) | 263 (4.8) | 44 (10.9) | 25 (3.2) | 10 (8.3) | 557 (17.4) | 134 (21.9) | 18 (10.5) | 22 (9.7) | 28 (0.1) | 3 (0.1) |
| Bipolar disorder, n(%) | 305 (9.6) | - | ~70 (3.4) | ~10 (7.7) | 196 (3.6) | 29 (7.2) | 71 (9.1) | 10 (8.3) | 557 (17.4) | 86 (14.0) | 11 (6.4) | 10 (4.4) | 2971 (7.5) | 943 (17.4) |
| Intellectual disability, n(%) | 68 (2.1) | - | ~60 (2.9) | ~10 (7.7) | 80 (1.5) | 14 (3.5) | 30 (3.8) | 11 (9.2) | 203 (6.3) | 73 (11.9) | 19 (11.0) | 37 (16.4) | 476 (1.2) | 225 (4.1) |
| Depressive disorder, n(%) | 651 (20.4) | - | ~480 (23.5) | ~50 (38.5) | 1,195 (22.0) | 160 (39.7) | 261 (33.3) | 37 (30.8) | 1,672 (52.1) | 291 (47.5) | 97 (56.4) | 91 (40.3) | 1,948 (4.9) | 299 (5.5) |
| Schizophrenia, n(%) | 226 (7.1) | - | ~210 (10.3) | ~20 (15.4) | 236 (4.4) | 23 (5.7) | 41 (5.2) | 10 (8.3) | 370 (11.5) | 104 (17.0) | 9 (5.2) | 30 (13.3) | 1,670 (4.2) | 685 (12.6) |
| Personality disorder, n(%) | 358 (11.2) | - | ~350 (16.8) | ~40 (30.8) | 1,122 (20.7) | 157 (39.0) | 115 (14.7) | 13 (10.8) | 764 (23.8) | 159 (25.9) | 17 (9.9) | 11 (4.9) | 1,524 (3.8) | 368 (6.8) |
| Eating disorders, n(%) | 68 (2.1) | - | ~30 (1.7) | ~10 (7.7) | 177 (3.3) | 29 (7.2) | 11 (1.4) | 1 (0.8) | 229 (7.1) | 28 (4.6) | 6 (3.5) | 3 (1.3) | 1,098 (2.8) | 230 (4.2) |
| Substance use disorder, n(%) | 549 (17.2) | - | ~340 (16.7) | ~40 (30.8) | 589 (10.9) | 86 (21.3) | 152 (19.4) | 21 (17.5) | 1,138 (35.5) | 213 (34.7) | 18 (10.5) | 21 (9.3) | 5,277 (13.3) | 1,466 (27.0) |

^+^ADHD medication (Yes) is defined as individuals had received an ADHD medication by study end, otherwise, (No). -not applicable. All numbers in Danish results are rounded to 10 due to data privacy.

# Supplementary table 10. Sensitivity analyses modelling ADHD as lifetime ADHD: association between ADHD and poor adherence to antidiabetic medication.

| **Subgroup** | **Year** | **Australia** | **Denmark** | **Netherlands** | **Norway** | **Sweden** | **United Kingdom** | **United States** | **Pooled analysis** |
| --- | --- | --- | --- | --- | --- | --- | --- | --- | --- |
| Total population | 1 | 0.90 (0.84 to 0.95) | 1.23 (1.14 to 1.32) | 1.37 (1.30 to 1.44) | 1.08 (0.95 to 1.23) | 1.12 (1.06 to 1.19) | 1.05 (0.88 to 1.24) | 0.78 (0.76 to 0.79) | 1.06 (0.91 to 1.22) |
| Total population | 2 | 0.95 (0.89 to 1.01) | 1.26 (1.16 to 1.36) | 1.40 (1.34 to 1.47) | 0.96 (0.84 to 1.11) | 1.21 (1.14 to 1.29) | 1.03 (0.87 to 1.22) | 0.69 (0.67 to 0.71) | 1.05 (0.88 to 1.25) |
| Total population | 5 | 1.00 (0.93 to 1.08) | 1.56 (1.40 to 1.73) | 1.57 (1.49 to 1.66) | 0.97 (0.78 to 1.21) | 1.48 (1.36 to 1.61) | 0.97 (0.81 to 1.18) | 0.60 (0.58 to 0.62) | 1.11 (0.85 to 1.45) |
| Young adults (18-34 years) | 1 | 0.79 (0.72 to 0.86) | 0.95 (0.83 to 1.09) | 0.79 (0.70 to 0.90) | 1.09 (0.91 to 1.31) | 0.65 (0.58 to 0.73) | 0.68 (0.52 to 0.90) | 0.64 (0.61 to 0.67) | 0.78 (0.68 to 0.91) |
| Young adults (18-34 years) | 2 | 0.81 (0.74 to 0.89) | 0.85 (0.73 to 1.00) | 0.84 (0.74 to 0.96) | 0.97 (0.78 to 1.20) | 0.64 (0.56 to 0.74) | 0.72 (0.53 to 0.96) | 0.55 (0.52 to 0.59) | 0.75 (0.65 to 0.87) |
| Young adults (18-34 years) | 5 | 0.83 (0.74 to 0.92) | 0.73 (0.58 to 0.91) | 0.93 (0.78 to 1.11) | 0.91 (0.64 to 1.28) | 0.63 (0.52 to 0.77) | 0.69 (0.49 to 0.97) | 0.45 (0.40 to 0.50) | 0.71 (0.58 to 0.87) |
| Middle-aged adults (35-64 years) | 1 | 0.94 (0.86 to 1.02) | 1.17 (1.06 to 1.28) | 1.25 (1.17 to 1.33) | 1.13 (0.94 to 1.36) | 1.02 (0.95 to 1.10) | 1.02 (0.78 to 1.33) | 0.78 (0.76 to 0.80) | 1.03 (0.90 to 1.17) |
| Middle-aged adults (35-64 years) | 2 | 1.03 (0.94 to 1.13) | 1.18 (1.07 to 1.31) | 1.24 (1.17 to 1.31) | 1.01 (0.83 to 1.23) | 1.03 (0.96 to 1.11) | 1.03 (0.80 to 1.34) | 0.68 (0.66 to 0.70) | 1.01 (0.87 to 1.18) |
| Middle-aged adults (35-64 years) | 5 | 1.12 (1.01 to 1.23) | 1.43 (1.25 to 1.63) | 1.34 (1.25 to 1.43) | 1.10 (0.83 to 1.47) | 1.13 (1.02 to 1.25) | 1.05 (0.79 to 1.39) | 0.59 (0.57 to 0.62) | 1.07 (0.86 to 1.34) |
| Older adults (≥65 years) | 1 | 1.32 (0.94 to 1.84) | 1.25 (0.98 to 1.59) | 1.01 (0.90 to 1.14) | – | 1.24 (0.95 to 1.62) | 1.05 (0.69 to 1.60) | 0.72 (0.69 to 0.76) | 1.05 (0.85 to 1.29) |
| Older adults (≥65 years) | 2 | 1.12 (0.80 to 1.56) | 1.56 (1.21 to 2.00) | 0.98 (0.87 to 1.10) | – | 1.19 (0.91 to 1.56) | 0.70 (0.46 to 1.07) | 0.66 (0.62 to 0.70) | 0.99 (0.75 to 1.29) |
| Older adults (≥65 years) | 5 | 1.11 (0.77 to 1.59) | 2.31 (1.66 to 3.22) | 1.07 (0.92 to 1.24) | – | 1.39 (0.95 to 2.03) | 0.52 (0.34 to 0.80) | 0.57 (0.52 to 0.62) | 1.02 (0.65 to 1.59) |
| Female | 1 | 0.83 (0.77 to 0.89) | 1.20 (1.08 to 1.32) | 1.45 (1.35 to 1.56) | 1.10 (0.93 to 1.30) | 1.06 (0.98 to 1.15) | 1.01 (0.79 to 1.28) | 0.78 (0.76 to 0.80) | 1.04 (0.88 to 1.23) |
| Female | 2 | 0.85 (0.78 to 0.92) | 1.24 (1.11 to 1.39) | 1.51 (1.41 to 1.62) | 0.95 (0.79 to 1.16) | 1.23 (1.12 to 1.35) | 0.97 (0.75 to 1.24) | 0.71 (0.69 to 0.73) | 1.04 (0.85 to 1.27) |
| Female | 5 | 0.87 (0.80 to 0.96) | 1.63 (1.39 to 1.90) | 1.80 (1.65 to 1.96) | 0.87 (0.64 to 1.18) | 1.51 (1.33 to 1.71) | 0.80 (0.61 to 1.05) | 0.61 (0.58 to 0.65) | 1.08 (0.79 to 1.48) |
| Male | 1 | 1.03 (0.94 to 1.13) | 1.29 (1.16 to 1.43) | 1.26 (1.18 to 1.35) | 1.11 (0.90 to 1.36) | 1.19 (1.10 to 1.29) | 1.18 (0.92 to 1.51) | 0.74 (0.72 to 0.77) | 1.09 (0.94 to 1.27) |
| Male | 2 | 1.14 (1.03 to 1.25) | 1.31 (1.17 to 1.47) | 1.31 (1.23 to 1.39) | 1.04 (0.83 to 1.29) | 1.21 (1.11 to 1.32) | 1.18 (0.93 to 1.50) | 0.65 (0.62 to 0.67) | 1.09 (0.90 to 1.32) |
| Male | 5 | 1.24 (1.11 to 1.39) | 1.54 (1.33 to 1.78) | 1.43 (1.33 to 1.54) | 1.21 (0.87 to 1.69) | 1.47 (1.32 to 1.64) | 1.22 (0.94 to 1.59) | 0.56 (0.53 to 0.59) | 1.18 (0.90 to 1.55) |

Odds ratio (95% CI). Models adjusted for sex, age and calendar year at initiation. Poor adherence to antidiabetic medication was defined as proportion of days covered (PDC) <80%. Sensitivity analysis modeling ADHD as a lifetime exposure, where individuals diagnosed with ADHD or receiving ADHD medication at any point by study end were considered exposed from baseline until the end of follow-up.

# Supplementary table 11. Sensitivity analyses modelling ADHD as lifetime ADHD: association between ADHD and first discontinuation of antidiabetic medication.

| Subgroup | Australia | Denmark | Netherlands | Norway | Sweden | United Kingdom | United States | Pooled analysis |
| --- | --- | --- | --- | --- | --- | --- | --- | --- |
| Total population | 0.95 (0.92 to 0.98) | 1.20 (1.15 to 1.25) | 1.23 (1.20 to 1.27) | 1.01 (0.94 to 1.08) | 1.04 (1.01 to 1.08) | 1.05 (0.95 to 1.16) | 0.90 (0.89 to 0.91) | 1.05 (0.96 to 1.14) |
| Young adults (18-34 years) | 0.84 (0.81 to 0.88) | 1.00 (0.94 to 1.07) | 0.97 (0.90 to 1.04) | 0.98 (0.90 to 1.07) | 0.79 (0.75 to 0.84) | 0.80 (0.69 to 0.93) | 0.90 (0.89 to 0.92) | 0.90 (0.84 to 0.96) |
| Middle-aged adults (35-64 years) | 1.01 (0.96 to 1.05) | 1.16 (1.09 to 1.23) | 1.16 (1.12 to 1.20) | 1.12 (1.00 to 1.24) | 1.02 (0.98 to 1.06) | 1.06 (0.90 to 1.24) | 0.91 (0.90 to 0.92) | 1.05 (0.98 to 1.13) |
| Older adults (≥65 years) | 1.36 (1.13 to 1.65) | 1.26 (1.07 to 1.49) | 1.23 (1.15 to 1.32) | - | 1.27 (1.08 to 1.49) | 0.90 (0.69 to 1.19) | 0.89 (0.87 to 0.92) | 1.14 (0.98 to 1.32) |
| Female | 0.89 (0.86 to 0.93) | 1.17 (1.11 to 1.24) | 1.31 (1.26 to 1.37) | 1.00 (0.92 to 1.08) | 0.99 (0.95 to 1.04) | 1.00 (0.88 to 1.15) | 0.91 (0.90 to 0.92) | 1.03 (0.93 to 1.15) |
| Male | 1.04 (0.99 to 1.10) | 1.25 (1.16 to 1.34) | 1.16 (1.12 to 1.21) | 1.07 (0.94 to 1.21) | 1.09 (1.04 to 1.14) | 1.14 (0.98 to 1.32) | 0.87 (0.86 to 0.89) | 1.08 (0.99 to 1.18) |

Hazard ratio (95% CI). Models adjusted for sex, age and calendar year at initiation. Sensitivity analysis modeling ADHD as a lifetime exposure, where individuals diagnosed with ADHD or receiving ADHD medication at any point by study end were considered exposed from baseline until the end of follow-up.

# Supplementary table 12. The number and proportion for individuals with ADHD identified before baseline versus during follow-up.

|  | Australia | Denmark | Netherlands | Norway | Sweden | UK | United States |
| --- | --- | --- | --- | --- | --- | --- | --- |
| Overall ADHD by the study end, N | 5,551 | 3160 | 8,490 | 1,170 | 5,066 | 553 | 64,349 |
| ADHD at baseline, n (%) | 3186 (57.4) | ~2,170 (68.7) | 5827 (68.6) | 904 (77.3) | 3821 (75.4) | 398 (72.0) | 45097 (70.1) |

“Overall ADHD by study end” refers to individuals identified with ADHD by diagnosis and/or ADHD medication at any point from the start of available data to the end of follow-up. “ADHD at baseline” refers to individuals with ADHD diagnosis and/or ADHD medication records before or at initiation of non-insulin antidiabetic medication. Percentages were calculated among individuals identified with ADHD by the end of follow-up within each country. In Denmark, counts were rounded to the nearest 10 because of data privacy requirements.

# Supplementary table 13. Sensitivity analyses redefining ADHD as a baseline exposure: association between ADHD and poor adherence to antidiabetic medication.

| **Study year** | **Subgroup name** | **Australia** | **Denmark** | **Netherlands** | **Norway** | **Sweden** | **UK** | **United States** | **Pooled analyses** |
| --- | --- | --- | --- | --- | --- | --- | --- | --- | --- |
| 1 | Total population | 0.81 (0.75, 0.87) | 1.05 (0.96, 1.14) | 1.26 (1.18, 1.34) | 1.00 (0.87, 1.15) | 1.03 (0.96, 1.10) | 1.03 (0.85, 1.26) | 1.03 (1.01, 1.06) | 1.02 (0.92, 1.13) |
| 2 | Total population | 0.84 (0.78, 0.91) | 1.05 (0.97, 1.15) | 1.33 (1.25, 1.40) | 0.85 (0.74, 0.99) | 1.12 (1.05, 1.19) | 1.11 (0.91, 1.37) | 1.03 (1.00, 1.05) | 1.04 (0.92, 1.17) |
| 5 | Total population | 0.83 (0.76, 0.91) | 1.09 (0.99, 1.20) | 1.49 (1.41, 1.58) | 0.94 (0.80, 1.10) | 1.41 (1.31, 1.51) | 1.10 (0.88, 1.39) | 1.08 (1.04, 1.11) | 1.12 (0.95, 1.31) |
| 1 | Young adults (18-34 years) | 0.72 (0.64, 0.80) | 0.89 (0.77, 1.04) | 0.78 (0.67, 0.90) | 1.03 (0.85, 1.25) | 0.64 (0.56, 0.72) | 0.62 (0.46, 0.84) | 0.98 (0.93, 1.03) | 0.80 (0.69, 0.93) |
| 2 | Young adults (18-34 years) | 0.75 (0.67, 0.85) | 0.86 (0.72, 1.01) | 0.82 (0.71, 0.94) | 0.89 (0.72, 1.09) | 0.64 (0.57, 0.74) | 0.69 (0.50, 0.96) | 1.05 (0.99, 1.11) | 0.81 (0.71, 0.93) |
| 5 | Young adults (18-34 years) | 0.76 (0.67, 0.87) | 0.83 (0.69, 1.01) | 0.93 (0.79, 1.09) | 0.93 (0.74, 1.18) | 0.82 (0.71, 0.95) | 0.70 (0.49, 1.04) | 1.17 (1.10, 1.24) | 0.89 (0.78, 1.01) |
| 1 | Middle-aged adults (35-64 years) | 0.88 (0.79, 0.98) | 1.05 (0.94, 1.18) | 1.17 (1.08, 1.26) | 1.02 (0.83, 1.26) | 0.96 (0.88, 1.05) | 0.97 (0.70, 1.34) | 1.04 (1.00, 1.07) | 1.02 (0.94, 1.10) |
| 2 | Middle-aged adults (35-64 years) | 0.95 (0.85, 1.06) | 1.10 (0.98, 1.22) | 1.17 (1.10, 1.26) | 0.88 (0.72, 1.08) | 1.02 (0.94, 1.11) | 1.12 (0.82, 1.54) | 1.02 (0.98, 1.06) | 1.04 (0.97, 1.11) |
| 5 | Middle-aged adults (35-64 years) | 0.96 (0.85, 1.10) | 1.16 (1.03, 1.30) | 1.26 (1.18, 1.34) | 1.01 (0.82, 1.25) | 1.21 (1.11, 1.32) | 1.11 (0.79, 1.58) | 1.07 (1.03, 1.11) | 1.12 (1.04, 1.21) |
| 1 | Older adults (≥65 years) | 1.31 (0.91, 1.91) | 0.76 (0.50, 1.11) | 0.83 (0.71, 0.97) | – | 1.46 (1.07, 1.96) | 1.63 (0.91, 2.96) | 1.05 (0.97, 1.13) | 1.08 (0.87, 1.35) |
| 2 | Older adults (≥65 years) | 1.09 (0.76, 1.60) | 0.83 (0.57, 1.19) | 0.88 (0.76, 1.02) | – | 1.43 (1.06, 1.91) | 1.00 (0.56, 1.81) | 1.00 (0.93, 1.09) | 1.01 (0.87, 1.17) |
| 5 | Older adults (≥65 years) | 1.09 (0.74, 1.66) | 0.77 (0.54, 1.12) | 0.96 (0.83, 1.12) | – | 2.09 (1.55, 2.84) | 0.81 (0.44, 1.55) | 1.01 (0.93, 1.11) | 1.08 (0.81, 1.43) |
| 1 | Female | 0.73 (0.66, 0.81) | 0.98 (0.87, 1.11) | 1.28 (1.17, 1.39) | 1.04 (0.87, 1.24) | 0.94 (0.86, 1.03) | 1.04 (0.78, 1.40) | 1.00 (0.97, 1.03) | 0.99 (0.86, 1.13) |
| 2 | Female | 0.73 (0.66, 0.82) | 0.99 (0.87, 1.12) | 1.37 (1.26, 1.49) | 0.87 (0.72, 1.05) | 1.08 (0.99, 1.19) | 1.05 (0.77, 1.45) | 1.01 (0.97, 1.04) | 1.00 (0.86, 1.16) |
| 5 | Female | 0.68 (0.60, 0.76) | 1.02 (0.90, 1.17) | 1.58 (1.45, 1.73) | 0.94 (0.77, 1.17) | 1.39 (1.26, 1.55) | 0.87 (0.62, 1.24) | 1.06 (1.02, 1.10) | 1.05 (0.84, 1.31) |
| 1 | Male | 0.97 (0.87, 1.09) | 1.18 (1.04, 1.33) | 1.18 (1.08, 1.29) | 0.97 (0.77, 1.23) | 1.15 (1.05, 1.26) | 1.17 (0.88, 1.55) | 1.08 (1.03, 1.13) | 1.10 (1.04, 1.17) |
| 2 | Male | 1.04 (0.93, 1.17) | 1.18 (1.04, 1.34) | 1.26 (1.17, 1.36) | 0.87 (0.69, 1.09) | 1.18 (1.07, 1.29) | 1.33 (1.01, 1.75) | 1.05 (1.01, 1.10) | 1.13 (1.04, 1.22) |
| 5 | Male | 1.12 (0.98, 1.28) | 1.22 (1.06, 1.39) | 1.40 (1.30, 1.50) | 0.96 (0.76, 1.21) | 1.44 (1.31, 1.58) | 1.45 (1.07, 2.00) | 1.10 (1.05, 1.16) | 1.23 (1.10, 1.37) |

Values are odds ratios with 95% confidence intervals. Baseline ADHD was defined as ADHD diagnosis and/or ADHD medication recorded before or at initiation of non-insulin antidiabetic medication. Poor adherence was defined as proportion of days covered <80% over the corresponding 1-, 2-, or 5-year follow-up period. Models were adjusted for sex, age, and calendar year at initiation of non-insulin antidiabetic medication. Pooled estimates were obtained using random-effects meta-analysis of country-specific estimates. Norway had no older adult group because the oldest individuals in the birth cohort register were born in 1967. “–” indicates that the estimate was not available.

# Supplementary table 14. Sensitivity analyses redefining ADHD as a baseline exposure: association between ADHD and first discontinuation of antidiabetic medication.

| **Subgroup name** | **Australia** | **Denmark** | **Netherlands** | **Norway** | **Sweden** | **UK** | **United States** | **Pooled analyses** |
| --- | --- | --- | --- | --- | --- | --- | --- | --- |
| Total population | 0.91 (0.87, 0.95) | 1.13 (1.07, 1.19) | 1.17 (1.13, 1.21) | 0.95 (0.88, 1.03) | 0.96 (0.92, 1.00) | 1.05 (0.94, 1.19) | 0.93 (0.92, 0.94) | 1.01 (0.93, 1.09) |
| Young adults (18-34 years) | 0.78 (0.74, 0.82) | 0.96 (0.88, 1.05) | 0.89 (0.82, 0.98) | 0.94 (0.85, 1.05) | 0.73 (0.68, 0.78) | 0.76 (0.64, 0.89) | 0.90 (0.88, 0.92) | 0.85 (0.78, 0.93) |
| Middle-aged adults (35-64 years) | 1.00 (0.94, 1.06) | 1.10 (1.02, 1.18) | 1.11 (1.06, 1.16) | 1.01 (0.89, 1.14) | 0.95 (0.90, 1.00) | 1.09 (0.90, 1.33) | 0.95 (0.93, 0.96) | 1.02 (0.96, 1.08) |
| Older adults (≥65 years) | 1.33 (1.07, 1.65) | 1.29 (0.99, 1.68) | 1.22 (1.11, 1.33) | – | 1.27 (1.07, 1.52) | 1.15 (0.80, 1.66) | 0.97 (0.93, 1.01) | 1.18 (1.04, 1.33) |
| Female | 0.85 (0.80, 0.89) | 1.07 (1.00, 1.15) | 1.24 (1.17, 1.30) | 0.94 (0.86, 1.04) | 0.93 (0.87, 0.98) | 0.96 (0.81, 1.14) | 0.94 (0.92, 0.95) | 0.98 (0.89, 1.08) |
| Male | 1.01 (0.95, 1.08) | 1.25 (1.15, 1.36) | 1.12 (1.07, 1.18) | 1.00 (0.87, 1.16) | 1.01 (0.95, 1.07) | 1.26 (1.06, 1.49) | 0.92 (0.90, 0.94) | 1.07 (0.98, 1.16) |

Values are hazard ratios with 95% confidence intervals. Models were adjusted for sex, age, and calendar year at initiation of non-insulin antidiabetic medication. Norway had no older adult group because the oldest individuals in the birth cohort register were born in 1967. “–” indicates that the estimate was not available.

# Supplementary table 15. Distribution of ADHD diagnosis and ADHD medication records by country and age group among individuals identified with ADHD.

| **ADHD definitions** | **Denmark** | | | **Netherlands** | | | **Norway** | | **Sweden** | | | **UK** | | | **United States** | | |
| --- | --- | --- | --- | --- | --- | --- | --- | --- | --- | --- | --- | --- | --- | --- | --- | --- | --- |
|  | **Young** | **Middle-aged** | **Older** | **Young** | **Middle-aged** | **Older** | **Young** | **Middle-aged** | **Young** | **Middle-aged** | **Older** | **Young** | **Middle-aged** | **Older** | **Young** | **Middle-aged** | **Older** |
| ADHD diagnosis and/or medication | 1070 | 1810 | 280 | 1,195 | 5,735 | 1,560 | 664 | 506 | 1,667 | 3,174 | 225 | 232 | 235 | 86 | 18,946 | 38,247 | 7,156 |
| Diagnosis only (no medication) | 100 (9.3) | 90 (5.0) | <10 | 133 (11.1) | 672 (11.7) | 77 (4.9) | 99 (14.9) | 72 (14.2) | 290 (17.4) | 551 (17.4) | 40 (17.8) | 136 (58.6) | 132 (56.2) | 58 (67.4) | 3420 (18.1) | 4720 (12.3) | 776 (10.8) |
| Medication only (no diagnosis) | 510 (47.7) | 1,220 (67.4) | >270 (96.4) | 549 (45.9) | 3373 (58.8) | 1415 (90.7) | 84 (12.7) | 91 (18.0) | 137 (8.2) | 528 (16.6) | 109 (48.4) | 26 (11.2) | 55 (23.4) | 25 (29.1) | 7573 (40.0) | 21990 (57.5) | 5392 (75.3) |
| Diagnosis and medication | 460 (43.0) | 510 (28.2) | <10 | 513 (42.9) | 1690 (29.5) | 68 (4.4) | 481 (72.4) | 343 (67.8) | 1240 (74.4) | 2095 (66.0) | 76 (33.8) | 70 (30.2) | 48 (20.4) | 3 (3.5) | 7953 (42.0) | 11537 (30.2) | 988 (13.8) |
| Diagnosis (±medication) | 560 (52.3) | 600 (33.1) | <10 | 646 (54.1) | 2362 (41.2) | 145 (9.3) | 580 (87.3) | 415 (82.0) | 1530 (91.8) | 2646 (83.4) | 116 (51.6) | 206 (88.8) | 180 (76.6) | 61 (70.9) | 11373 (60.0) | 16257 (42.5) | 1764 (24.7) |
| Medication (±diagnosis) | 970 (90.7) | 1,730 (95.6) | >270 (96.4) | 1062 (88.9) | 5063 (88.3) | 1483 (95.1) | 565 (85.1) | 434 (85.8) | 1377 (82.6) | 2623 (82.6) | 185 (82.2) | 96 (41.4) | 103 (43.8) | 28 (32.6) | 15526 (81.9) | 33527 (87.7) | 6380 (89.2) |

Values are n (%) unless otherwise indicated. Percentages were calculated among individuals identified with ADHD by diagnosis and/or medication within each country and age group. “Diagnosis only” refers to individuals with an ADHD diagnosis record but no ADHD medication record; “medication only” refers to individuals with an ADHD medication record but no ADHD diagnosis record; and “diagnosis and medication” refers to individuals with both types of records. “Diagnosis (±medication)” includes diagnosis only and diagnosis plus medication; “medication (±diagnosis)” includes medication only and diagnosis plus medication. ADHD diagnosis data were unavailable in Australia. In Denmark, cell counts were rounded to the nearest 10 or suppressed because of data privacy requirements. Norway had no older adult group because the oldest individuals in the birth cohort register were born in 1967.

# Supplementary table 16. Sensitivity analyses on ADHD ascertainment source: association between ADHD and poor adherence to antidiabetic medication.

| **Exposure level** | **Study year** | **Subgroups** | **Australia** | **Denmark** | **Netherlands** | **Norway** | **Sweden** | **UK** | **United States** | **Pooled analyses** |
| --- | --- | --- | --- | --- | --- | --- | --- | --- | --- | --- |
| Diagnosis and medication | 1 | Total population | — | 1.03 (0.91, 1.17) | 1.46 (1.32, 1.62) | 1.03 (0.89, 1.20) | 1.12 (1.05, 1.20) | 1.10 (0.77, 1.58) | 1.14 (1.10, 1.18) | 1.15 (1.03, 1.28) |
| Diagnosis and medication | 2 | Total population | — | 1.02 (0.90, 1.16) | 1.71 (1.57, 1.87) | 0.91 (0.79, 1.06) | 1.21 (1.13, 1.30) | 1.07 (0.74, 1.56) | 1.19 (1.15, 1.24) | 1.18 (0.98, 1.41) |
| Diagnosis and medication | 5 | Total population | — | 1.14 (0.99, 1.32) | 1.98 (1.81, 2.16) | 0.96 (0.81, 1.13) | 1.48 (1.37, 1.59) | 1.21 (0.80, 1.90) | 1.42 (1.36, 1.48) | 1.35 (1.09, 1.66) |
| Diagnosis only (no medication) | 1 | Total population | — | 1.41 (1.06, 1.89) | 1.25 (1.05, 1.47) | 1.15 (0.83, 1.59) | 1.01 (0.88, 1.16) | 0.99 (0.79, 1.24) | 1.18 (1.12, 1.24) | 1.14 (1.05, 1.25) |
| Diagnosis only (no medication) | 2 | Total population | — | 1.26 (0.94, 1.71) | 1.14 (0.98, 1.32) | 1.13 (0.81, 1.59) | 1.10 (0.96, 1.26) | 1.04 (0.83, 1.30) | 1.29 (1.22, 1.37) | 1.18 (1.08, 1.29) |
| Diagnosis only (no medication) | 5 | Total population | — | 1.37 (0.99, 1.93) | 1.24 (1.08, 1.43) | 1.19 (0.82, 1.76) | 1.45 (1.26, 1.68) | 0.90 (0.71, 1.15) | 1.45 (1.36, 1.56) | 1.28 (1.11, 1.48) |
| Medication only (no diagnosis) | 1 | Total population | 0.90 (0.84, 0.95) | 1.15 (1.05, 1.26) | 1.30 (1.22, 1.38) | 1.22 (0.89, 1.68) | 1.21 (1.05, 1.40) | 1.17 (0.79, 1.73) | 0.97 (0.94, 0.99) | 1.10 (0.98, 1.24) |
| Medication only (no diagnosis) | 2 | Total population | 0.95 (0.89, 1.01) | 1.16 (1.06, 1.27) | 1.29 (1.22, 1.37) | 1.06 (0.77, 1.47) | 1.34 (1.16, 1.54) | 0.95 (0.65, 1.41) | 1.00 (0.97, 1.03) | 1.11 (0.99, 1.25) |
| Medication only (no diagnosis) | 5 | Total population | 1.00 (0.93, 1.08) | 1.19 (1.08, 1.32) | 1.39 (1.32, 1.48) | 1.37 (0.96, 2.01) | 1.54 (1.32, 1.79) | 0.99 (0.65, 1.54) | 1.15 (1.12, 1.19) | 1.23 (1.08, 1.39) |
| Diagnosis and medication | 1 | Young adults (18-34 years) | — | 0.78 (0.65, 0.94) | 0.77 (0.64, 0.93) | 0.97 (0.80, 1.19) | 0.71 (0.63, 0.81) | 0.81 (0.49, 1.35) | 1.06 (1.00, 1.12) | 0.85 (0.73, 0.99) |
| Diagnosis and medication | 2 | Young adults (18-34 years) | — | 0.76 (0.62, 0.94) | 0.87 (0.72, 1.04) | 0.85 (0.69, 1.05) | 0.69 (0.60, 0.79) | 0.74 (0.44, 1.29) | 1.20 (1.12, 1.28) | 0.86 (0.71, 1.03) |
| Diagnosis and medication | 5 | Young adults (18-34 years) | — | 0.74 (0.59, 0.94) | 0.96 (0.79, 1.17) | 0.87 (0.69, 1.11) | 0.83 (0.71, 0.97) | 0.70 (0.38, 1.36) | 1.46 (1.35, 1.57) | 0.93 (0.74, 1.17) |
| Diagnosis only (no medication) | 1 | Young adults (18-34 years) | — | 1.14 (0.74, 1.80) | 0.59 (0.40, 0.85) | 1.13 (0.74, 1.75) | 0.68 (0.53, 0.88) | 0.60 (0.42, 0.86) | 1.16 (1.06, 1.27) | 0.85 (0.64, 1.11) |
| Diagnosis only (no medication) | 2 | Young adults (18-34 years) | — | 0.98 (0.62, 1.60) | 0.71 (0.50, 1.01) | 1.18 (0.74, 1.96) | 0.71 (0.54, 0.93) | 0.79 (0.53, 1.18) | 1.36 (1.23, 1.51) | 0.93 (0.72, 1.20) |
| Diagnosis only (no medication) | 5 | Young adults (18-34 years) | — | 1.19 (0.69, 2.18) | 0.91 (0.63, 1.33) | 1.41 (0.81, 2.64) | 0.81 (0.60, 1.10) | 0.71 (0.46, 1.13) | 1.58 (1.41, 1.77) | 1.06 (0.80, 1.41) |
| Medication only (no diagnosis) | 1 | Young adults (18-34 years) | 0.79 (0.72, 0.86) | 1.11 (0.92, 1.34) | 0.99 (0.82, 1.18) | 1.35 (0.85, 2.19) | 0.63 (0.44, 0.90) | 0.84 (0.37, 2.01) | 0.97 (0.91, 1.03) | 0.93 (0.80, 1.08) |
| Medication only (no diagnosis) | 2 | Young adults (18-34 years) | 0.81 (0.74, 0.89) | 1.02 (0.83, 1.27) | 0.95 (0.80, 1.14) | 1.40 (0.84, 2.48) | 0.88 (0.59, 1.35) | 0.42 (0.19, 1.00) | 1.05 (0.99, 1.13) | 0.95 (0.84, 1.07) |
| Medication only (no diagnosis) | 5 | Young adults (18-34 years) | 0.83 (0.74, 0.92) | 0.90 (0.71, 1.14) | 1.10 (0.90, 1.34) | 1.57 (0.85, 3.20) | 1.27 (0.79, 2.18) | 0.57 (0.23, 1.64) | 1.27 (1.18, 1.37) | 1.04 (0.87, 1.25) |
| Diagnosis and medication | 1 | Middle-aged adults (35-64 years) | — | 0.95 (0.80, 1.14) | 1.06 (0.93, 1.20) | 1.16 (0.93, 1.45) | 0.98 (0.90, 1.08) | 0.96 (0.52, 1.70) | 1.13 (1.08, 1.18) | 1.06 (0.98, 1.14) |
| Diagnosis and medication | 2 | Middle-aged adults (35-64 years) | — | 0.93 (0.78, 1.10) | 1.25 (1.12, 1.38) | 1.03 (0.83, 1.28) | 1.05 (0.96, 1.14) | 0.95 (0.53, 1.69) | 1.18 (1.12, 1.24) | 1.10 (0.99, 1.21) |
| Diagnosis and medication | 5 | Middle-aged adults (35-64 years) | — | 1.07 (0.89, 1.29) | 1.43 (1.29, 1.58) | 1.08 (0.86, 1.37) | 1.22 (1.11, 1.33) | 1.08 (0.58, 2.13) | 1.44 (1.35, 1.52) | 1.26 (1.12, 1.42) |
| Diagnosis only (no medication) | 1 | Middle-aged adults (35-64 years) | — | 1.31 (0.87, 1.98) | 1.13 (0.92, 1.37) | 1.28 (0.79, 2.05) | 0.92 (0.77, 1.09) | 1.06 (0.74, 1.50) | 1.17 (1.09, 1.25) | 1.11 (0.99, 1.23) |
| Diagnosis only (no medication) | 2 | Middle-aged adults (35-64 years) | — | 1.19 (0.79, 1.82) | 0.97 (0.81, 1.16) | 1.17 (0.72, 1.88) | 0.95 (0.80, 1.12) | 1.02 (0.72, 1.45) | 1.26 (1.16, 1.36) | 1.08 (0.95, 1.24) |
| Diagnosis only (no medication) | 5 | Middle-aged adults (35-64 years) | — | 1.21 (0.78, 1.92) | 0.98 (0.83, 1.15) | 1.13 (0.69, 1.91) | 1.24 (1.05, 1.48) | 1.01 (0.69, 1.47) | 1.47 (1.34, 1.61) | 1.19 (1.01, 1.40) |
| Medication only (no diagnosis) | 1 | Middle-aged adults (35-64 years) | 0.94 (0.86, 1.02) | 1.17 (1.04, 1.31) | 1.39 (1.27, 1.51) | 1.15 (0.75, 1.76) | 1.33 (1.12, 1.59) | 1.01 (0.57, 1.74) | 0.96 (0.93, 0.99) | 1.13 (0.98, 1.30) |
| Medication only (no diagnosis) | 2 | Middle-aged adults (35-64 years) | 1.03 (0.94, 1.13) | 1.21 (1.08, 1.36) | 1.30 (1.21, 1.40) | 0.89 (0.58, 1.36) | 1.32 (1.11, 1.57) | 1.15 (0.67, 1.98) | 0.97 (0.94, 1.01) | 1.13 (1.01, 1.27) |
| Medication only (no diagnosis) | 5 | Middle-aged adults (35-64 years) | 1.12 (1.01, 1.24) | 1.27 (1.12, 1.44) | 1.35 (1.26, 1.46) | 1.29 (0.83, 2.06) | 1.41 (1.18, 1.68) | 1.13 (0.64, 2.08) | 1.12 (1.08, 1.17) | 1.23 (1.13, 1.34) |
| Diagnosis and medication | 1 | Older adults (≥65 years) | — | 1.62 (0.19, 13.48) | 0.95 (0.53, 1.65) | — | 2.13 (1.34, 3.34) | — | 1.19 (1.02, 1.38) | 1.35 (0.90, 2.01) |
| Diagnosis and medication | 2 | Older adults (≥65 years) | — | 1.09 (0.13, 9.13) | 1.03 (0.61, 1.73) | — | 1.69 (1.07, 2.65) | — | 1.15 (0.98, 1.35) | 1.20 (1.00, 1.45) |
| Diagnosis and medication | 5 | Older adults (≥65 years) | — | 0.56 (0.07, 4.71) | 1.52 (0.88, 2.75) | — | 2.56 (1.60, 4.21) | 1.11 (0.10, 24.07) | 1.42 (1.17, 1.71) | 1.64 (1.17, 2.30) |
| Diagnosis only (no medication) | 1 | Older adults (≥65 years) | — | 0.79 (0.11, 4.04) | 0.69 (0.39, 1.20) | — | 0.89 (0.41, 1.75) | 0.94 (0.55, 1.58) | 1.10 (0.94, 1.30) | 0.99 (0.80, 1.23) |
| Diagnosis only (no medication) | 2 | Older adults (≥65 years) | — | 1.12 (0.21, 6.09) | 0.56 (0.34, 0.92) | — | 1.02 (0.52, 1.93) | 0.68 (0.40, 1.13) | 1.24 (1.03, 1.49) | 0.88 (0.60, 1.28) |
| Diagnosis only (no medication) | 5 | Older adults (≥65 years) | — | 0.61 (0.11, 3.30) | 0.82 (0.50, 1.36) | — | 2.07 (1.11, 4.03) | 0.41 (0.24, 0.69) | 1.30 (1.06, 1.59) | 0.93 (0.52, 1.68) |
| Medication only (no diagnosis) | 1 | Older adults (≥65 years) | 1.32 (0.94, 1.85) | 0.86 (0.66, 1.11) | 0.97 (0.85, 1.10) | — | 1.27 (0.85, 1.89) | 1.67 (0.76, 3.77) | 0.96 (0.90, 1.02) | 0.97 (0.92, 1.03) |
| Medication only (no diagnosis) | 2 | Older adults (≥65 years) | 1.12 (0.80, 1.57) | 0.95 (0.74, 1.22) | 1.00 (0.89, 1.13) | — | 1.58 (1.08, 2.30) | 0.93 (0.42, 2.09) | 1.01 (0.95, 1.08) | 1.02 (0.97, 1.08) |
| Medication only (no diagnosis) | 5 | Older adults (≥65 years) | 1.11 (0.78, 1.61) | 1.00 (0.79, 1.29) | 1.03 (0.91, 1.17) | — | 1.88 (1.28, 2.79) | 0.86 (0.37, 2.13) | 1.17 (1.08, 1.26) | 1.13 (1.00, 1.27) |
| Diagnosis and medication | 1 | Female | — | 0.91 (0.77, 1.07) | 1.77 (1.54, 2.04) | 1.01 (0.85, 1.22) | 1.02 (0.93, 1.13) | 1.16 (0.70, 1.96) | 1.13 (1.08, 1.18) | 1.14 (0.93, 1.39) |
| Diagnosis and medication | 2 | Female | — | 1.01 (0.85, 1.21) | 2.02 (1.77, 2.31) | 0.90 (0.74, 1.10) | 1.18 (1.07, 1.30) | 1.19 (0.70, 2.15) | 1.22 (1.17, 1.28) | 1.22 (0.96, 1.55) |
| Diagnosis and medication | 5 | Female | — | 1.06 (0.88, 1.30) | 2.26 (1.97, 2.60) | 0.94 (0.76, 1.18) | 1.49 (1.33, 1.66) | 0.94 (0.52, 1.82) | 1.44 (1.36, 1.52) | 1.33 (1.01, 1.75) |
| Diagnosis only (no medication) | 1 | Female | — | 1.23 (0.83, 1.83) | 1.18 (0.90, 1.53) | 1.30 (0.87, 1.96) | 1.02 (0.83, 1.24) | 1.08 (0.79, 1.48) | 1.22 (1.14, 1.31) | 1.18 (1.09, 1.28) |
| Diagnosis only (no medication) | 2 | Female | — | 1.05 (0.70, 1.60) | 1.20 (0.93, 1.53) | 1.10 (0.72, 1.73) | 1.06 (0.87, 1.29) | 1.03 (0.75, 1.44) | 1.32 (1.22, 1.43) | 1.18 (1.04, 1.33) |
| Diagnosis only (no medication) | 5 | Female | — | 1.25 (0.80, 2.04) | 1.48 (1.16, 1.90) | 1.41 (0.85, 2.47) | 1.44 (1.16, 1.79) | 0.78 (0.56, 1.13) | 1.47 (1.34, 1.60) | 1.31 (1.09, 1.58) |
| Medication only (no diagnosis) | 1 | Female | 0.83 (0.77, 0.90) | 1.24 (1.10, 1.40) | 1.30 (1.19, 1.43) | 1.45 (0.96, 2.22) | 1.17 (0.95, 1.43) | 0.69 (0.39, 1.21) | 0.96 (0.93, 0.99) | 1.08 (0.91, 1.27) |
| Medication only (no diagnosis) | 2 | Female | 0.85 (0.78, 0.92) | 1.20 (1.05, 1.36) | 1.31 (1.21, 1.43) | 1.34 (0.86, 2.14) | 1.28 (1.05, 1.57) | 0.64 (0.36, 1.12) | 0.99 (0.96, 1.03) | 1.08 (0.92, 1.27) |
| Medication only (no diagnosis) | 5 | Female | 0.87 (0.80, 0.96) | 1.27 (1.10, 1.47) | 1.48 (1.35, 1.62) | 1.42 (0.85, 2.49) | 1.52 (1.23, 1.90) | 0.72 (0.40, 1.35) | 1.15 (1.10, 1.19) | 1.20 (1.00, 1.44) |
| Diagnosis and medication | 1 | Male | — | 1.24 (1.02, 1.50) | 1.14 (0.98, 1.31) | 1.10 (0.85, 1.41) | 1.26 (1.14, 1.39) | 1.15 (0.66, 1.96) | 1.11 (1.04, 1.17) | 1.16 (1.09, 1.24) |
| Diagnosis and medication | 2 | Male | — | 1.06 (0.87, 1.28) | 1.45 (1.29, 1.63) | 0.95 (0.75, 1.22) | 1.25 (1.13, 1.38) | 1.08 (0.63, 1.83) | 1.11 (1.04, 1.19) | 1.17 (1.03, 1.33) |
| Diagnosis and medication | 5 | Male | — | 1.26 (1.02, 1.55) | 1.76 (1.57, 1.97) | 0.99 (0.77, 1.28) | 1.48 (1.34, 1.64) | 1.63 (0.91, 3.07) | 1.37 (1.27, 1.48) | 1.39 (1.19, 1.63) |
| Diagnosis only (no medication) | 1 | Male | — | 1.74 (1.14, 2.66) | 1.11 (0.89, 1.36) | 0.93 (0.52, 1.62) | 1.06 (0.87, 1.27) | 0.98 (0.70, 1.36) | 1.13 (1.04, 1.22) | 1.12 (1.05, 1.20) |
| Diagnosis only (no medication) | 2 | Male | — | 1.60 (1.04, 2.51) | 1.01 (0.84, 1.22) | 1.19 (0.69, 2.05) | 1.17 (0.98, 1.41) | 1.14 (0.83, 1.56) | 1.26 (1.15, 1.38) | 1.19 (1.08, 1.31) |
| Diagnosis only (no medication) | 5 | Male | — | 1.55 (0.97, 2.54) | 1.07 (0.90, 1.26) | 0.97 (0.56, 1.74) | 1.50 (1.24, 1.81) | 1.08 (0.78, 1.52) | 1.44 (1.30, 1.60) | 1.28 (1.10, 1.50) |
| Medication only (no diagnosis) | 1 | Male | 1.03 (0.94, 1.13) | 1.07 (0.93, 1.22) | 1.30 (1.18, 1.42) | 0.99 (0.59, 1.63) | 1.29 (1.04, 1.59) | 1.97 (1.15, 3.38) | 0.97 (0.93, 1.01) | 1.13 (1.00, 1.28) |
| Medication only (no diagnosis) | 2 | Male | 1.14 (1.03, 1.26) | 1.15 (1.00, 1.31) | 1.28 (1.18, 1.39) | 0.83 (0.50, 1.34) | 1.41 (1.15, 1.73) | 1.41 (0.82, 2.43) | 0.99 (0.95, 1.04) | 1.16 (1.04, 1.29) |
| Medication only (no diagnosis) | 5 | Male | 1.24 (1.11, 1.39) | 1.14 (1.00, 1.31) | 1.34 (1.24, 1.45) | 1.33 (0.80, 2.26) | 1.55 (1.26, 1.92) | 1.34 (0.75, 2.50) | 1.16 (1.10, 1.22) | 1.26 (1.16, 1.36) |

Models were adjusted for sex, age, and calendar year at initiation of non-insulin antidiabetic medication. Odds ratios compare each ADHD ascertainment-source category with individuals with no ADHD diagnosis and no ADHD medication record. Poor adherence was defined as proportion of days covered <80% over the corresponding 1-, 2-, or 5-year follow-up period. “Diagnosis only” refers to ADHD diagnosis without ADHD medication; “medication only” refers to ADHD medication without ADHD diagnosis; and “diagnosis and medication” refers to individuals with both ADHD diagnosis and ADHD medication records. Pooled estimates were obtained using random-effects meta-analysis of country-specific estimates. ADHD diagnosis data were unavailable in Australia; therefore, Australia contributed only to the medication-only category. Norway had no older adult group because the oldest individuals in the birth cohort register were born in 1967. “—” indicates that the estimate was not available because of unavailable data, small cell counts, or model non-convergence.

# Supplementary table 17. Sensitivity analyses on ADHD ascertainment source: association between ADHD and first discontinuation of antidiabetic medication.

| **Exposure level** | **Subgroups** | **Australia** | **Denmark** | **Netherlands** | **Norway** | **Sweden** | **UK** | **United States** | **Pooled analyses** |
| --- | --- | --- | --- | --- | --- | --- | --- | --- | --- |
| Diagnosis and medication | Total population | — | 1.15 (1.06, 1.24) | 1.25 (1.18, 1.32) | 0.99 (0.92, 1.07) | 1.05 (1.00, 1.09) | 1.21 (0.98, 1.49) | 0.89 (0.88, 0.91) | 1.07 (0.96, 1.19) |
| Diagnosis only (no medication) | Total population | — | 1.19 (1.01, 1.42) | 1.11 (1.01, 1.21) | 1.00 (0.84, 1.19) | 0.92 (0.85, 1.00) | 0.97 (0.84, 1.11) | 1.01 (0.98, 1.03) | 1.02 (0.95, 1.09) |
| Medication only (no diagnosis) | Total population | 0.95 (0.92, 0.98) | 1.23 (1.16, 1.30) | 1.24 (1.19, 1.28) | 1.11 (0.94, 1.31) | 1.15 (1.06, 1.25) | 1.14 (0.91, 1.43) | 0.88 (0.87, 0.89) | 1.09 (0.98, 1.21) |
| Diagnosis and medication | Young adults (18-34 years) | — | 0.96 (0.86, 1.06) | 0.98 (0.88, 1.08) | 0.96 (0.87, 1.07) | 0.79 (0.74, 0.85) | 0.96 (0.74, 1.25) | 0.88 (0.86, 0.90) | 0.91 (0.84, 0.98) |
| Diagnosis only (no medication) | Young adults (18-34 years) | — | 1.03 (0.82, 1.30) | 0.93 (0.75, 1.15) | 0.87 (0.70, 1.09) | 0.79 (0.69, 0.90) | 0.75 (0.62, 0.92) | 0.98 (0.95, 1.02) | 0.89 (0.80, 0.99) |
| Medication only (no diagnosis) | Young adults (18-34 years) | 0.84 (0.81, 0.88) | 1.04 (0.94, 1.15) | 0.98 (0.89, 1.09) | 1.21 (0.97, 1.52) | 0.75 (0.62, 0.91) | 0.69 (0.45, 1.07) | 0.89 (0.87, 0.91) | 0.92 (0.82, 1.02) |
| Diagnosis and medication | Middle-aged adults (35-64 years) | — | 1.00 (0.89, 1.13) | 1.15 (1.08, 1.22) | 1.09 (0.96, 1.24) | 1.01 (0.96, 1.07) | 1.14 (0.81, 1.61) | 0.91 (0.89, 0.93) | 1.03 (0.95, 1.12) |
| Diagnosis only (no medication) | Middle-aged adults (35-64 years) | — | 1.09 (0.84, 1.42) | 1.04 (0.94, 1.16) | 1.38 (1.05, 1.82) | 0.88 (0.79, 0.97) | 0.94 (0.76, 1.18) | 1.00 (0.97, 1.04) | 1.01 (0.92, 1.10) |
| Medication only (no diagnosis) | Middle-aged adults (35-64 years) | 1.01 (0.96, 1.05) | 1.23 (1.15, 1.33) | 1.19 (1.14, 1.25) | 1.04 (0.82, 1.33) | 1.22 (1.11, 1.35) | 1.32 (0.96, 1.81) | 0.89 (0.87, 0.90) | 1.10 (0.99, 1.23) |
| Diagnosis and medication | Older adults (≥65 years) | — | 2.15 (0.54, 8.59) | 0.98 (0.67, 1.44) | — | 1.52 (1.14, 2.04) | 0.84 (0.21, 3.37) | 0.92 (0.85, 0.98) | 1.11 (0.83, 1.49) |
| Diagnosis only (no medication) | Older adults (≥65 years) | — | 0.95 (0.31, 2.94) | 1.21 (0.89, 1.64) | — | 0.98 (0.67, 1.43) | 0.83 (0.59, 1.17) | 1.04 (0.96, 1.12) | 1.03 (0.96, 1.11) |
| Medication only (no diagnosis) | Older adults (≥65 years) | 1.36 (1.13, 1.65) | 1.26 (1.07, 1.49) | 1.24 (1.15, 1.34) | — | 1.28 (1.03, 1.60) | 1.14 (0.69, 1.89) | 0.87 (0.84, 0.90) | 1.17 (1.00, 1.37) |
| Diagnosis and medication | Female | — | 1.09 (0.99, 1.20) | 1.34 (1.24, 1.45) | 0.99 (0.90, 1.08) | 0.98 (0.93, 1.03) | 1.31 (1.00, 1.73) | 0.90 (0.89, 0.92) | 1.07 (0.94, 1.22) |
| Diagnosis only (no medication) | Female | — | 1.11 (0.89, 1.38) | 1.17 (1.01, 1.36) | 0.98 (0.80, 1.20) | 0.94 (0.84, 1.06) | 0.95 (0.79, 1.14) | 1.04 (1.01, 1.07) | 1.03 (0.98, 1.08) |
| Medication only (no diagnosis) | Female | 0.89 (0.86, 0.93) | 1.23 (1.14, 1.32) | 1.32 (1.25, 1.39) | 1.10 (0.89, 1.35) | 1.10 (0.98, 1.23) | 0.88 (0.64, 1.22) | 0.89 (0.88, 0.90) | 1.06 (0.93, 1.20) |
| Diagnosis and medication | Male | — | 1.24 (1.09, 1.41) | 1.18 (1.10, 1.27) | 1.03 (0.89, 1.19) | 1.12 (1.05, 1.18) | 1.20 (0.87, 1.65) | 0.86 (0.83, 0.88) | 1.08 (0.96, 1.22) |
| Diagnosis only (no medication) | Male | — | 1.37 (1.05, 1.80) | 1.08 (0.96, 1.21) | 1.09 (0.78, 1.54) | 0.93 (0.83, 1.04) | 1.02 (0.84, 1.25) | 0.97 (0.93, 1.01) | 1.02 (0.94, 1.11) |
| Medication only (no diagnosis) | Male | 1.04 (0.99, 1.10) | 1.24 (1.13, 1.35) | 1.17 (1.11, 1.23) | 1.22 (0.93, 1.60) | 1.22 (1.07, 1.37) | 1.48 (1.07, 2.05) | 0.86 (0.84, 0.88) | 1.13 (1.00, 1.28) |

Models were adjusted for sex, age, and calendar year at initiation of non-insulin antidiabetic medication. Hazard ratios compare each ADHD ascertainment-source category with individuals with no ADHD diagnosis and no ADHD medication record. “Diagnosis only” refers to ADHD diagnosis without ADHD medication; “medication only” refers to ADHD medication without ADHD diagnosis; and “diagnosis and medication” refers to individuals with both ADHD diagnosis and ADHD medication records. Pooled estimates were obtained using random-effects meta-analysis of country-specific estimates. ADHD diagnosis data were unavailable in Australia; therefore, Australia contributed only to the medication-only category. Norway had no older adult group because the oldest individuals in the birth cohort register were born in 1967. “—” indicates that the estimate was not available because of unavailable data, small cell counts, or model non-convergence.

# Supplementary table 18. Sensitivity analyses using country-specific prescription length and grace period: association between ADHD and poor adherence to antidiabetic medication.

| **Subgroup** | **Year** | **Australia*** | **Denmark** | **Netherlands** | **Norway** | **Sweden** | **United Kingdom*** | **United States** | **Pooled analysis** |
| --- | --- | --- | --- | --- | --- | --- | --- | --- | --- |
| Total population | 1 | 0.78 (0.73 to 0.84) | 1.06 (0.97 to 1.15) | 1.32 (1.24 to 1.41) | 1.01 (0.88 to 1.16) | 1.02 (0.96 to 1.09) | 0.92 (0.76 to 1.12) | 0.96 (0.94 to 0.98) | 1.00 (0.89 to 1.13) |
| Total population | 2 | 0.79 (0.73 to 0.84) | 1.05 (0.97 to 1.14) | 1.35 (1.28 to 1.42) | 0.89 (0.78 to 1.03) | 1.12 (1.06 to 1.20) | 0.91 (0.76 to 1.10) | 0.98 (0.96 to 1.01) | 1.01 (0.88 to 1.15) |
| Total population | 5 | 0.74 (0.70 to 0.79) | 1.11 (1.02 to 1.20) | 1.47 (1.40 to 1.55) | 0.95 (0.82 to 1.10) | 1.36 (1.27 to 1.44) | 0.88 (0.74 to 1.06) | 1.17 (1.14 to 1.20) | 1.07 (0.90 to 1.29) |
| Young adults (18-34 years) | 1 | 0.67 (0.60 to 0.74) | 0.86 (0.74 to 1.00) | 0.78 (0.68 to 0.90) | 1.03 (0.85 to 1.23) | 0.63 (0.56 to 0.71) | 0.51 (0.38 to 0.69) | 0.95 (0.90 to 0.99) | 0.77 (0.65 to 0.91) |
| Young adults (18-34 years) | 2 | 0.66 (0.60 to 0.73) | 0.83 (0.71 to 0.98) | 0.83 (0.72 to 0.95) | 0.94 (0.77 to 1.15) | 0.65 (0.57 to 0.74) | 0.52 (0.38 to 0.71) | 1.03 (0.98 to 1.09) | 0.78 (0.66 to 0.92) |
| Young adults (18-34 years) | 5 | 0.59 (0.53 to 0.65) | 0.77 (0.65 to 0.92) | 0.92 (0.80 to 1.06) | 0.95 (0.76 to 1.19) | 0.77 (0.67 to 0.89) | 0.50 (0.36 to 0.70) | 1.27 (1.21 to 1.35) | 0.81 (0.64 to 1.01) |
| Middle-aged adults (35-64 years) | 1 | 0.89 (0.80 to 0.98) | 1.06 (0.95 to 1.18) | 1.23 (1.15 to 1.33) | 1.05 (0.86 to 1.28) | 0.96 (0.89 to 1.05) | 1.01 (0.74 to 1.39) | 0.95 (0.92 to 0.97) | 1.01 (0.93 to 1.11) |
| Middle-aged adults (35-64 years) | 2 | 0.92 (0.83 to 1.01) | 1.06 (0.95 to 1.17) | 1.20 (1.13 to 1.28) | 0.91 (0.74 to 1.10) | 1.02 (0.95 to 1.10) | 1.03 (0.76 to 1.39) | 0.96 (0.93 to 0.99) | 1.02 (0.94 to 1.10) |
| Middle-aged adults (35-64 years) | 5 | 0.89 (0.81 to 0.97) | 1.15 (1.04 to 1.29) | 1.28 (1.21 to 1.36) | 1.00 (0.82 to 1.22) | 1.18 (1.09 to 1.27) | 1.01 (0.76 to 1.34) | 1.15 (1.11 to 1.19) | 1.10 (1.00 to 1.22) |
| Older adults (≥65 years) | 1 | 1.15 (0.81 to 1.63) | 1.03 (0.74 to 1.43) | 0.98 (0.84 to 1.14) | – | 1.45 (1.08 to 1.94) | 1.25 (0.72 to 2.16) | 0.98 (0.92 to 1.04) | 1.06 (0.94 to 1.20) |
| Older adults (≥65 years) | 2 | 1.07 (0.76 to 1.51) | 1.19 (0.89 to 1.61) | 0.99 (0.87 to 1.14) | – | 1.53 (1.16 to 2.01) | 0.84 (0.51 to 1.39) | 0.97 (0.91 to 1.04) | 1.08 (0.93 to 1.25) |
| Older adults (≥65 years) | 5 | 1.16 (0.83 to 1.61) | 1.17 (0.87 to 1.56) | 1.07 (0.94 to 1.22) | – | 2.11 (1.60 to 2.78) | 0.73 (0.46 to 1.16) | 1.15 (1.07 to 1.24) | 1.19 (0.93 to 1.52) |
| Female | 1 | 0.67 (0.61 to 0.74) | 0.99 (0.88 to 1.11) | 1.32 (1.20 to 1.45) | 1.06 (0.89 to 1.26) | 0.93 (0.86 to 1.02) | 0.88 (0.66 to 1.17) | 0.94 (0.92 to 0.97) | 0.95 (0.82 to 1.12) |
| Female | 2 | 0.66 (0.60 to 0.72) | 0.99 (0.88 to 1.11) | 1.34 (1.23 to 1.46) | 0.93 (0.77 to 1.11) | 1.09 (0.99 to 1.19) | 0.82 (0.62 to 1.09) | 0.98 (0.95 to 1.01) | 0.96 (0.81 to 1.14) |
| Female | 5 | 0.60 (0.55 to 0.65) | 1.04 (0.92 to 1.18) | 1.49 (1.37 to 1.61) | 0.96 (0.79 to 1.18) | 1.31 (1.19 to 1.44) | 0.79 (0.60 to 1.04) | 1.15 (1.11 to 1.19) | 1.02 (0.80 to 1.28) |
| Male | 1 | 1.00 (0.90 to 1.10) | 1.18 (1.05 to 1.34) | 1.27 (1.17 to 1.37) | 0.97 (0.77 to 1.22) | 1.15 (1.05 to 1.26) | 1.08 (0.82 to 1.42) | 0.98 (0.94 to 1.02) | 1.09 (1.00 to 1.19) |
| Male | 2 | 1.03 (0.93 to 1.14) | 1.16 (1.04 to 1.31) | 1.32 (1.24 to 1.42) | 0.88 (0.71 to 1.10) | 1.19 (1.09 to 1.29) | 1.13 (0.87 to 1.46) | 0.98 (0.95 to 1.02) | 1.10 (1.00 to 1.22) |
| Male | 5 | 1.02 (0.93 to 1.12) | 1.22 (1.09 to 1.38) | 1.43 (1.34 to 1.52) | 0.96 (0.77 to 1.19) | 1.42 (1.30 to 1.54) | 1.06 (0.83 to 1.36) | 1.18 (1.13 to 1.24) | 1.20 (1.07 to 1.34) |

Odds ratio (95% CI). Models adjusted for sex, age and calendar year at initiation. Sensitivity analysis using country-specific gaps different from 135 days to define outcomes, including a more conservative maximum prescription length of 120 days with a 60-day grace period in Australia, and a maximum prescription length of 90 days with a 56-day grace period in the UK. No additional gap definition was used in other countries.

# Supplementary table 19. Sensitivity analyses using country-specific prescription length and grace period: association between ADHD and first discontinuation of antidiabetic medication.

| **Subgroup** | **Australia*** | **Denmark** | **Netherlands** | **Norway** | **Sweden** | **United Kingdom*** | **United States** | **Pooled analysis** |
| --- | --- | --- | --- | --- | --- | --- | --- | --- |
| Total population | 0.94 (0.90 to 0.98) | 1.12 (1.07 to 1.18) | 1.19 (1.15 to 1.23) | 0.97 (0.90 to 1.05) | 0.97 (0.93 to 1.01) | 1.08 (0.96 to 1.21) | 0.90 (0.89 to 0.91) | 1.02 (0.94 to 1.10) |
| Young adults (18-34 years) | 0.80 (0.76 to 0.85) | 0.97 (0.89 to 1.05) | 0.92 (0.85 to 1.00) | 0.97 (0.88 to 1.07) | 0.74 (0.69 to 0.79) | 0.75 (0.63 to 0.88) | 0.89 (0.87 to 0.91) | 0.86 (0.79 to 0.93) |
| Middle-aged adults (35-64 years) | 1.05 (0.99 to 1.12) | 1.10 (1.02 to 1.18) | 1.12 (1.07 to 1.17) | 1.04 (0.92 to 1.17) | 0.97 (0.92 to 1.02) | 1.16 (0.97 to 1.39) | 0.91 (0.90 to 0.93) | 1.04 (0.97 to 1.11) |
| Older adults (≥65 years) | 1.21 (0.96 to 1.54) | 1.25 (0.98 to 1.60) | 1.26 (1.16 to 1.37) | - | 1.30 (1.09 to 1.54) | 1.10 (0.80 to 1.51) | 0.92 (0.89 to 0.96) | 1.15 (1.01 to 1.32) |
| Female | 0.85 (0.81 to 0.90) | 1.07 (1.00 to 1.15) | 1.26 (1.20 to 1.32) | 0.96 (0.88 to 1.06) | 0.93 (0.88 to 0.98) | 1.01 (0.86 to 1.19) | 0.91 (0.90 to 0.92) | 0.99 (0.90 to 1.10) |
| Male | 1.09 (1.02 to 1.16) | 1.24 (1.15 to 1.35) | 1.13 (1.08 to 1.18) | 1.04 (0.91 to 1.19) | 1.04 (0.98 to 1.10) | 1.24 (1.06 to 1.46) | 0.88 (0.86 to 0.90) | 1.08 (0.99 to 1.18) |

Hazard ratio (95% CI). Models adjusted for sex, age and calendar year at initiation. Sensitivity analysis using country-specific gaps different from 135 days to define outcomes, including a more conservative maximum prescription length of 120 days with a 60-day grace period in Australia, and a maximum prescription length of 90 days with a 56-day grace period in the UK. No additional gap definition was used in other countries.

# Supplementary table 20. Sensitivity analyses using country-specific prescription length and grace period: association between ADHD medication and poor adherence to antidiabetic medication.

| **Subgroup** | **Year** | **Australia*** | **Denmark** | **Netherlands** | **Norway** | **Sweden** | **United Kingdom*** | **United States** | **Pooled analysis** |
| --- | --- | --- | --- | --- | --- | --- | --- | --- | --- |
| Total population | 1 | 0.65 (0.57 to 0.74) | 0.56 (0.48 to 0.67) | 0.51 (0.44 to 0.59) | 0.78 (0.60 to 1.03) | 0.66 (0.58 to 0.75) | 0.30 (0.17 to 0.51) | 0.19 (0.19 to 0.20) | 0.48 (0.33 to 0.70) |
| Total population | 2 | 0.64 (0.57 to 0.74) | 0.58 (0.49 to 0.68) | 0.52 (0.46 to 0.59) | 0.72 (0.55 to 0.94) | 0.64 (0.56 to 0.73) | 0.28 (0.17 to 0.47) | 0.17 (0.16 to 0.18) | 0.46 (0.31 to 0.69) |
| Total population | 5 | 0.63 (0.55 to 0.71) | 0.60 (0.51 to 0.70) | 0.52 (0.46 to 0.59) | 0.66 (0.50 to 0.86) | 0.61 (0.54 to 0.69) | 0.29 (0.18 to 0.48) | 0.16 (0.15 to 0.17) | 0.45 (0.30 to 0.67) |
| Young adults (18-34 years) | 1 | 0.57 (0.47 to 0.69) | 0.48 (0.36 to 0.65) | 0.69 (0.51 to 0.94) | 0.78 (0.54 to 1.12) | 0.56 (0.45 to 0.70) | 0.41 (0.19 to 0.89) | 0.22 (0.20 to 0.23) | 0.49 (0.35 to 0.70) |
| Young adults (18-34 years) | 2 | 0.55 (0.45 to 0.67) | 0.52 (0.39 to 0.70) | 0.53 (0.40 to 0.71) | 0.68 (0.47 to 0.97) | 0.54 (0.43 to 0.67) | 0.27 (0.12 to 0.59) | 0.19 (0.17 to 0.20) | 0.44 (0.31 to 0.63) |
| Young adults (18-34 years) | 5 | 0.52 (0.43 to 0.63) | 0.49 (0.37 to 0.66) | 0.50 (0.38 to 0.66) | 0.57 (0.40 to 0.83) | 0.54 (0.43 to 0.67) | 0.25 (0.12 to 0.55) | 0.18 (0.16 to 0.19) | 0.41 (0.29 to 0.59) |
| Middle-aged adults (35-64 years) | 1 | 0.73 (0.60 to 0.88) | 0.61 (0.49 to 0.76) | 0.57 (0.46 to 0.70) | 0.78 (0.51 to 1.19) | 0.71 (0.60 to 0.84) | 0.32 (0.13 to 0.77) | 0.21 (0.20 to 0.22) | 0.52 (0.36 to 0.77) |
| Middle-aged adults (35-64 years) | 2 | 0.73 (0.60 to 0.88) | 0.61 (0.50 to 0.75) | 0.60 (0.51 to 0.71) | 0.77 (0.52 to 1.16) | 0.70 (0.60 to 0.82) | 0.38 (0.17 to 0.82) | 0.18 (0.17 to 0.19) | 0.52 (0.35 to 0.78) |
| Middle-aged adults (35-64 years) | 5 | 0.73 (0.60 to 0.88) | 0.65 (0.53 to 0.80) | 0.60 (0.52 to 0.70) | 0.76 (0.51 to 1.13) | 0.63 (0.54 to 0.74) | 0.35 (0.17 to 0.73) | 0.17 (0.16 to 0.18) | 0.51 (0.33 to 0.77) |
| Older adults (≥65 years) | 1 | 0.71 (0.36 to 1.37) | - | 0.30 (0.21 to 0.43) | - | 1.42 (0.80 to 2.53) | 0.04 (0.00 to 0.37) | 0.10 (0.09 to 0.11) | 0.31 (0.10 to 0.96) |
| Older adults (≥65 years) | 2 | 0.74 (0.38 to 1.44) | - | 0.34 (0.25 to 0.47) | - | 0.92 (0.52 to 1.63) | 0.04 (0.00 to 0.43) | 0.09 (0.08 to 0.10) | 0.30 (0.11 to 0.82) |
| Older adults (≥65 years) | 5 | 0.69 (0.36 to 1.33) | - | 0.34 (0.25 to 0.47) | - | 0.97 (0.55 to 1.70) | 0.15 (0.03 to 0.70) | 0.09 (0.08 to 0.10) | 0.32 (0.13 to 0.80) |
| Female | 1 | 0.60 (0.50 to 0.71) | 0.57 (0.45 to 0.71) | 0.49 (0.41 to 0.59) | 0.73 (0.53 to 1.02) | 0.59 (0.49 to 0.71) | 0.25 (0.12 to 0.54) | 0.21 (0.20 to 0.22) | 0.46 (0.32 to 0.66) |
| Female | 2 | 0.58 (0.48 to 0.69) | 0.58 (0.46 to 0.73) | 0.52 (0.44 to 0.62) | 0.69 (0.50 to 0.96) | 0.62 (0.52 to 0.74) | 0.22 (0.10 to 0.46) | 0.18 (0.17 to 0.19) | 0.45 (0.30 to 0.66) |
| Female | 5 | 0.56 (0.47 to 0.67) | 0.57 (0.45 to 0.71) | 0.50 (0.42 to 0.59) | 0.65 (0.47 to 0.91) | 0.57 (0.48 to 0.68) | 0.24 (0.12 to 0.50) | 0.17 (0.16 to 0.18) | 0.43 (0.29 to 0.64) |
| Male | 1 | 0.73 (0.60 to 0.89) | 0.56 (0.44 to 0.72) | 0.56 (0.44 to 0.71) | 0.91 (0.56 to 1.47) | 0.74 (0.62 to 0.89) | 0.35 (0.16 to 0.75) | 0.17 (0.16 to 0.18) | 0.51 (0.33 to 0.80) |
| Male | 2 | 0.74 (0.61 to 0.90) | 0.58 (0.45 to 0.73) | 0.53 (0.44 to 0.64) | 0.77 (0.49 to 1.24) | 0.65 (0.54 to 0.78) | 0.34 (0.16 to 0.69) | 0.15 (0.14 to 0.16) | 0.48 (0.30 to 0.75) |
| Male | 5 | 0.72 (0.59 to 0.87) | 0.62 (0.49 to 0.79) | 0.55 (0.46 to 0.65) | 0.66 (0.42 to 1.05) | 0.64 (0.54 to 0.76) | 0.32 (0.17 to 0.64) | 0.14 (0.14 to 0.15) | 0.47 (0.30 to 0.73) |

Odds ratio (95% CI). Models adjusted for sex, age and calendar year at initiation. Sensitivity analysis using country-specific gaps different from 135 days to define outcomes, including a more conservative maximum prescription length of 120 days with a 60-day grace period in Australia, and a maximum prescription length of 90 days with a 56-day grace period in the UK. No additional gap definition was used in other countries.

# Supplementary table 21. Age-stratified baseline characteristics.

| Characteristics | Australia | | | Denmark | | | Netherlands | | | Norway | | Sweden | | | United Kingdom | | | United States | | |
| --- | --- | --- | --- | --- | --- | --- | --- | --- | --- | --- | --- | --- | --- | --- | --- | --- | --- | --- | --- | --- |
|  | Young | Middle-aged | Older | Young | Middle-aged | Older | Young | Middle-aged | Older | Young | Middle-aged | Young | Middle-aged | Older | Young | Middle-aged | Older | Young | Middle-aged | Older |
| Among individuals initiating non-insulin antidiabetic medication who also had ADHD | | | | | | | | | | | | | | | | | | | | |
| N | 2,920 | 2,477 | 154 | 1070 | 1810 | 280 | 1,195 | 5,735 | 1,560 | 664 | 506 | 1,667 | 3,174 | 225 | 232 | 235 | 86 | 18,946 | 38,247 | 7,156 |
| Age at baseline, years | 25 [21, 30] | 45 [40, 52] | 68 [66, 72] | 27 [23,30] | 47 [41,53] | 71 [67,74] | 28 [23, 32] | 50 [44, 56] | 72 [68, 79] | 26 [23, 30] | 42 [38, 45] | 27 [24, 31] | 48 [41, 54] | 68 [66, 71] | 26 [23, 30] | 49 [41, 56] | 73 [68, 81] | 27 [23, 31] | 49 [42, 56] | 69 [67, 73] |
| Sex, female, n (%) | 2109 (72.2) | 1280 (51.7) | 60 (39.0) | 760 (71.0) | 850 (47.0) | 120 (42.9) | 620 (51.9) | 2332 (40.7) | 743 (47.6) | 518 (78.0) | 254 (50.2) | 1086 (65.1) | 1396 (44.0) | 97 (43.1) | 149 (64.2) | 90 (38.3) | 44 (51.2) | 14917 (78.7) | 24514 (64.1) | 3552 (49.6) |
| Time to end of follow-up, years | 3.57 [1.64, 5.87] | 3.56 [1.59, 5.78] | 3.42 [1.54, 5.19] | 4.64 [1.92,7.88] | 3.69 [1.50,6.92] | 2.54 [1.13,5.19] | 3.95 [1.86, 7.34] | 4.72 [2.13, 7.81] | 3.52 [1.46, 6.38] | 3.67 [1.71, 6.88] | 3.04 [1.21, 5.86] | 3.31 [1.46, 6.41] | 3.64 [1.62, 6.55] | 2.87 [1.58, 5.32] | 4.73 [2.27, 7.53] | 4.99 [2.76, 8.06] | 6.13 [3.64, 8.07] | 2.48 [1.02, 4.49] | 2.32 [0.96, 4.12] | 1.93 [0.78, 3.69] |
| At least one psychiatric comorbidity, n (%) | 930 (31.8) | 861 (34.8) | 40 (26.0) | 540 (50.5) | 720 (39.8) | 30 (10.7) | 616 (51.5) | 2318 (40.4) | 211 (13.5) | 387 (58.3) | 309 (61.1) | 1278 (76.7) | 2491 (78.5) | 124 (55.1) | 151 (65.1) | 169 (71.9) | 31 (36.0) | 8547 (45.1) | 14748 (38.6) | 1820 (25.4) |
| Among individuals initiating non-insulin antidiabetic medication | | | | | | | | | | | | | | | | | | | | |
| N | 81,160 | 230,433 | 115,385 | 18280 | 113530 | 83450 | 15,422 | 289,057 | 234,184 | 9,691 | 14,528 | 19,324 | 175,562 | 158,144 | 22,545 | 123,265 | 88,786 | 170,654 | 1,044,935 | 634,862 |
| Age at baseline, years | 28 [24, 31] | 52 [44, 58] | 72 [68, 77] | 28 [25,31] | 54 [47,59] | 72 [68,78] | 30 [26, 32] | 55 [48, 60] | 73 [68, 78] | 28 [24, 31] | 43 [39, 46] | 29 [26, 32] | 55 [48, 60] | 72 [68, 78] | 29 [25, 32] | 53 [46, 59] | 73 [69, 80] | 28 [24, 32] | 54 [47, 59] | 71 [68, 75] |
| Sex, female, n(%) | 68105 (83.9) | 117267 (50.9) | 52601 (45.6) | 15,060 (82.4) | 48,460 (42.7) | 37,870 (45.4) | 9043 (58.6) | 123043 (42.6) | 114195 (48.8) | 8188 (84.5) | 6883 (47.4) | 14549 (75.3) | 70968 (40.4) | 72203 (45.7) | 18833 (83.5) | 52101 (42.3) | 41771 (47.0) | 133841 (78.4) | 558814 (53.5) | 308055 (48.5) |
| Time to end of follow-up, years | 4.43 [2.15, 6.58] | 4.19 [1.91, 6.47] | 3.61 [1.55, 5.91] | 5.39 [2.40,8.58] | 5.03 [2.21,8.38] | 4.39 [1.86,7.65] | 4.84 [2.13, 8.12] | 5.68 [2.76, 8.56] | 4.52 [2.01, 7.51] | 4.13 [1.87, 7.21] | 3.38 [1.55, 6.13] | 3.09 [1.40, 6.04] | 4.59 [2.13, 7.70] | 3.88 [1.82, 6.68] | 6.32 [3.65, 8.63] | 6.44 [3.61, 8.78] | 5.68 [2.71, 8.30] | 2.11 [0.74, 4.15] | 1.95 [0.71, 3.84] | 1.57 [0.51, 3.32] |
| At least one psychiatric comorbidity, n(%) | 8565 (10.6) | 25567 (11.1) | 9125 (7.9) | 2,920 (16.0) | 10,900 (9.6) | 4,380 (5.2) | 2844 (18.4) | 26894 (9.3) | 7304 (3.1) | 2369 (24.4) | 3021 (20.8) | 4844 (25.1) | 31704 (18.1) | 17240 (10.9) | 7042 (31.2) | 40955 (33.2) | 20325 (22.9) | 38466 (22.5) | 199186 (19.1) | 75024 (11.8) |

Values are median [IQR] unless otherwise indicated. Age groups were defined at initiation of non-insulin antidiabetic medication: young adults, 18–34 years; middle-aged adults, 35–64 years; and older adults, ≥65 years. In Denmark, counts were rounded to 10 because of data privacy requirements. Norway had no older adult group because the oldest individuals in the birth cohort register were born in 1967.

# Supplementary table 22. Sensitivity analyses among individuals with confirmed type 2 diabetes diagnosis.

A ADHD and poor adherence (Odds ratio [95% CI])

| **Population** | **Analysis** | **Subgroup** | **1 year follow up** | **2 years follow up** | **5 years follow up** |
| --- | --- | --- | --- | --- | --- |
| Type 2 diabetes diagnosis within 1 year before initiation | ADHD and poor adherence | Total population | 0.96 (0.71 to 1.31) | 1.09 (0.82 to 1.44) | 1.00 (0.76 to 1.30) |
| Type 2 diabetes diagnosis within 1 year before initiation | ADHD and poor adherence | Male | 0.87 (0.59 to 1.28) | 1.05 (0.74 to 1.49) | 0.99 (0.71 to 1.37) |
| Type 2 diabetes diagnosis within 1 year before initiation | ADHD and poor adherence | Female | 1.10 (0.65 to 1.86) | 1.11 (0.69 to 1.78) | 0.98 (0.63 to 1.53) |
| Type 2 diabetes diagnosis within 1 year before initiation | ADHD and poor adherence | Young adults (18-34 years) | 0.69 (0.41 to 1.14) | 0.70 (0.44 to 1.11) | 0.77 (0.50 to 1.20) |
| Type 2 diabetes diagnosis within 1 year before initiation | ADHD and poor adherence | Middle-aged adults (35-64 years) | 0.79 (0.50 to 1.23) | 0.98 (0.66 to 1.46) | 0.88 (0.61 to 1.27) |
| Type 2 diabetes diagnosis within 1 year before initiation | ADHD and poor adherence | Older adults (≥65 years) | 2.24 (0.80 to 6.28) | 2.06 (0.83 to 5.14) | 1.19 (0.53 to 2.71) |
| Type 2 diabetes diagnosis before initiation | ADHD and poor adherence | Total population | 0.98 (0.76 to 1.27) | 1.01 (0.80 to 1.27) | 0.94 (0.76 to 1.17) |
| Type 2 diabetes diagnosis before initiation | ADHD and poor adherence | Male | 0.94 (0.68 to 1.29) | 1.04 (0.78 to 1.39) | 0.97 (0.74 to 1.27) |
| Type 2 diabetes diagnosis before initiation | ADHD and poor adherence | Female | 1.03 (0.68 to 1.56) | 0.93 (0.63 to 1.37) | 0.89 (0.62 to 1.27) |
| Type 2 diabetes diagnosis before initiation | ADHD and poor adherence | Young adults (18-34 years) | 0.59 (0.37 to 0.92) | 0.68 (0.45 to 1.03) | 0.68 (0.46 to 1.02) |
| Type 2 diabetes diagnosis before initiation | ADHD and poor adherence | Middle-aged adults (35-64 years) | 0.96 (0.67 to 1.38) | 1.03 (0.74 to 1.44) | 0.98 (0.72 to 1.34) |
| Type 2 diabetes diagnosis before initiation | ADHD and poor adherence | Older adults (≥65 years) | 1.15 (0.62 to 2.15) | 0.79 (0.45 to 1.37) | 0.66 (0.40 to 1.09) |

B ADHD medication and poor adherence (Odds ratio [95% CI])

| **Population** | **Analysis** | **Subgroup** | **1 year follow up** | **2 years follow up** | **5 years follow up** |
| --- | --- | --- | --- | --- | --- |
| Type 2 diabetes diagnosis within 1 year before initiation | ADHD medication and poor adherence | Total population | 0.47 (0.19 to 1.14) | 0.45 (0.20 to 1.01) | 0.33 (0.15 to 0.74) |
| Type 2 diabetes diagnosis within 1 year before initiation | ADHD medication and poor adherence | Male | 0.48 (0.15 to 1.51) | 0.38 (0.13 to 1.09) | 0.31 (0.11 to 0.82) |
| Type 2 diabetes diagnosis within 1 year before initiation | ADHD medication and poor adherence | Female | 0.66 (0.14 to 3.12) | 0.92 (0.21 to 3.96) | 0.54 (0.13 to 2.32) |
| Type 2 diabetes diagnosis within 1 year before initiation | ADHD medication and poor adherence | Young adults (18-34 years) | 0.93 (0.21 to 4.20) | 1.13 (0.27 to 4.84) | 0.56 (0.13 to 2.34) |
| Type 2 diabetes diagnosis within 1 year before initiation | ADHD medication and poor adherence | Middle-aged adults (35-64 years) | 0.46 (0.14 to 1.52) | 0.38 (0.13 to 1.12) | 0.31 (0.11 to 0.86) |
| Type 2 diabetes diagnosis within 1 year before initiation | ADHD medication and poor adherence | Older adults (≥65 years) | 0.00 (0.00 to 0.00) | 0.00 (0.00 to 0.00) | 0.00 (0.00 to 0.00) |
| Type 2 diabetes diagnosis before initiation | ADHD medication and poor adherence | Total population | 0.34 (0.16 to 0.71) | 0.39 (0.20 to 0.75) | 0.37 (0.20 to 0.69) |
| Type 2 diabetes diagnosis before initiation | ADHD medication and poor adherence | Male | 0.48 (0.20 to 1.16) | 0.48 (0.21 to 1.08) | 0.44 (0.20 to 0.93) |
| Type 2 diabetes diagnosis before initiation | ADHD medication and poor adherence | Female | 0.19 (0.05 to 0.73) | 0.28 (0.09 to 0.93) | 0.29 (0.09 to 0.88) |
| Type 2 diabetes diagnosis before initiation | ADHD medication and poor adherence | Young adults (18-34 years) | 0.73 (0.20 to 2.60) | 0.80 (0.24 to 2.67) | 0.67 (0.21 to 2.15) |
| Type 2 diabetes diagnosis before initiation | ADHD medication and poor adherence | Middle-aged adults (35-64 years) | 0.39 (0.15 to 1.01) | 0.42 (0.18 to 1.00) | 0.35 (0.15 to 0.80) |
| Type 2 diabetes diagnosis before initiation | ADHD medication and poor adherence | Older adults (≥65 years) | 0.00 (0.00 to 0.00) | 0.00 (0.00 to 0.00) | 0.11 (0.01 to 1.13) |

C ADHD and discontinuation (Hazard ratio [95% CI])

| **Population** | **Analysis** | **Total population** | **Male** | **Female** | **Young adults (18-34 years)** | **Middle-aged adults (35-64 years)** | **Older adults (≥65 years)** |
| --- | --- | --- | --- | --- | --- | --- | --- |
| Type 2 diabetes diagnosis within 1 year before initiation | ADHD and discontinuation | 1.30 (1.08 to 1.56) | 1.19 (0.95 to 1.50) | 1.49 (1.09 to 2.05) | 0.90 (0.66 to 1.24) | 1.24 (0.97 to 1.60) | 1.25 (0.73 to 2.14) |
| Type 2 diabetes diagnosis before initiation | ADHD and discontinuation | 1.22 (1.06 to 1.41) | 1.14 (0.96 to 1.37) | 1.34 (1.05 to 1.70) | 0.87 (0.68 to 1.12) | 1.13 (0.92 to 1.39) | 1.17 (0.83 to 1.64) |

Models adjusted for sex, age and calendar year at initiation. These sensitivity analyses were conducted in the UK, where primary care records and type 2 diabetes diagnosis were available.

The first restriction for the study population is to require a clinical type 2 diabetes diagnosis within one year before baseline.

The second restriction for the study population is to require a clinical type 2 diabetes diagnosis before baseline.

# Supplementary table 23. E-values for the associations of ADHD and ADHD medication use with antidiabetic medication discontinuation and poor adherence.

A. E-values for analyses of ADHD and poor adherence to antidiabetic medication

| **Subgroup** | **Follow-up** | **Pooled odds ratio (95% CI)** | **E-value for point estimate** | **E-value: CI limit** |
| --- | --- | --- | --- | --- |
| Total population | 1 year | 1.01 (0.90–1.14) | 1.08 | 1.00 |
| Total population | 2 years | 1.03 (0.91–1.17) | 1.14 | 1.00 |
| Total population | 5 years | 1.12 (0.95–1.31) | 1.31 | 1.00 |
| Young adults | 1 year | 0.79 (0.68–0.91) | 1.50 | 1.27 |
| Young adults | 2 years | 0.81 (0.71–0.93) | 1.46 | 1.23 |
| Young adults | 5 years | 0.87 (0.73–1.03) | 1.35 | 1.00 |
| Middle-aged adults | 1 year | 1.01 (0.91–1.11) | 1.08 | 1.00 |
| Middle-aged adults | 2 years | 1.02 (0.94–1.11) | 1.11 | 1.00 |
| Middle-aged adults | 5 years | 1.12 (1.03–1.23) | 1.31 | 1.14 |
| Older adults | 1 year | 1.09 (0.95–1.25) | 1.26 | 1.00 |
| Older adults | 2 years | 1.07 (0.92–1.24) | 1.22 | 1.00 |
| Older adults | 5 years | 1.17 (0.91–1.52) | 1.38 | 1.00 |
| Female | 1 year | 0.97 (0.83–1.13) | 1.14 | 1.00 |
| Female | 2 years | 0.98 (0.84–1.15) | 1.11 | 1.00 |
| Female | 5 years | 1.05 (0.86–1.29) | 1.18 | 1.00 |
| Male | 1 year | 1.09 (1.00–1.20) | 1.26 | 1.00 |
| Male | 2 years | 1.12 (1.00–1.24) | 1.31 | 1.00 |
| Male | 5 years | 1.23 (1.11–1.37) | 1.46 | 1.29 |

B. E-values for analyses of ADHD medication use and poor adherence among individuals with ADHD

| **Subgroup** | **Follow-up** | **Pooled odds ratio (95% CI)** | **E-value for point estimate** | **E-value: CI limit** |
| --- | --- | --- | --- | --- |
| Total population | 1 year | 0.48 (0.33–0.70) | 2.24 | 1.68 |
| Total population | 2 years | 0.46 (0.31–0.69) | 2.31 | 1.70 |
| Total population | 5 years | 0.45 (0.30–0.67) | 2.35 | 1.74 |
| Young adults, 18–34 years | 1 year | 0.49 (0.35–0.70) | 2.21 | 1.68 |
| Young adults, 18–34 years | 2 years | 0.44 (0.31–0.63) | 2.38 | 1.83 |
| Young adults, 18–34 years | 5 years | 0.41 (0.29–0.58) | 2.50 | 1.95 |
| Middle-aged adults, 35–64 years | 1 year | 0.52 (0.36–0.76) | 2.12 | 1.56 |
| Middle-aged adults, 35–64 years | 2 years | 0.52 (0.35–0.78) | 2.12 | 1.52 |
| Middle-aged adults, 35–64 years | 5 years | 0.51 (0.33–0.77) | 2.15 | 1.54 |
| Older adults, ≥65 years | 1 year | 0.31 (0.10–0.96) | 2.99 | 1.17 |
| Older adults, ≥65 years | 2 years | 0.30 (0.11–0.86) | 3.05 | 1.37 |
| Older adults, ≥65 years | 5 years | 0.33 (0.13–0.84) | 2.88 | 1.41 |
| Female | 1 year | 0.46 (0.32–0.66) | 2.31 | 1.76 |
| Female | 2 years | 0.45 (0.30–0.66) | 2.35 | 1.76 |
| Female | 5 years | 0.43 (0.29–0.64) | 2.42 | 1.81 |
| Male | 1 year | 0.51 (0.33–0.79) | 2.15 | 1.50 |
| Male | 2 years | 0.48 (0.30–0.75) | 2.24 | 1.58 |
| Male | 5 years | 0.47 (0.30–0.73) | 2.28 | 1.62 |

C. E-values for analyses of ADHD and first discontinuation of antidiabetic medication

| **Subgroup** | **Hazard ratio (95% CI)** | **E-value for point estimate** | **E-value: CI limit** |
| --- | --- | --- | --- |
| Total population | 1.02 (0.94–1.10) | 1.13 | 1.00 |
| Young adults, 18–34 years | 0.86 (0.79–0.93) | 1.46 | 1.28 |
| Middle-aged adults, 35–64 years | 1.03 (0.96–1.09) | 1.17 | 1.00 |
| Older adults, ≥65 years | 1.18 (1.03–1.35) | 1.49 | 1.17 |
| Female | 0.99 (0.90–1.10) | 1.09 | 1.00 |
| Male | 1.07 (0.98–1.17) | 1.27 | 1.00 |

# Supplementary table 24. Post-hoc analyses of age-by-sex stratified analyses within the Australian cohort.

A ADHD and poor adherence

| **Subgroup** | **1 year follow up** | **2 years follow up** | **5 years follow up** |
| --- | --- | --- | --- |
|  | **Odds ratio (95% CI)** | | |
| Total population | 0.80 (0.74 to 0.85) | 0.79 (0.74 to 0.85) | 0.75 (0.70 to 0.80) |
| Young male adults (18-34 years) | 0.83 (0.70 to 0.99) | 0.84 (0.71 to 1.01) | 0.85 (0.71 to 1.01) |
| Young female adults (18-34 years) | 0.59 (0.52 to 0.66) | 0.56 (0.50 to 0.64) | 0.45 (0.40 to 0.51) |
| Middle-age male adults (35-64 years) | 0.93 (0.81 to 1.06) | 0.97 (0.85 to 1.11) | 0.96 (0.85 to 1.09) |
| Middle-age female adults (35-64 years) | 0.85 (0.74 to 0.99) | 0.88 (0.76 to 1.02) | 0.83 (0.73 to 0.96) |
| Old-age male adults (≥65 years) | 1.58 (0.98 to 2.53) | 1.40 (0.88 to 2.21) | 1.28 (0.83 to 1.96) |
| Old-age female adults (≥65 years) | 0.91 (0.53 to 1.54) | 0.73 (0.43 to 1.24) | 0.99 (0.59 to 1.65) |

B ADHD and first discontinuation

| **Subgroup** | **Hazard Ratio (95% CI)** |
| --- | --- |
| Total population | 0.92 (0.89 to 0.96) |
| Young male adults (18-34 years) | 0.84 (0.76 to 0.92) |
| Young female adults (18-34 years) | 0.75 (0.70 to 0.80) |
| Middle-age male adults (35-64 years) | 1.02 (0.94 to 1.11) |
| Middle-age female adults (35-64 years) | 0.98 (0.90 to 1.07) |
| Old-age male adults (≥65 years) | 1.47 (1.12 to 1.92) |
| Old-age female adults (≥65 years) | 1.14 (0.80 to 1.60) |

Models adjusted for sex, age and calendar year at initiation.

# Supplementary table 25. Country-specific *p* values for interaction terms in analyses examining the association of ADHD with antidiabetic medication discontinuation and poor adherence.

A. ADHD × age group interaction tests

| **Outcome** | **Interaction term** | **Australia** | **Denmark** | **Netherlands** | **Norway** | **Sweden** | **UK** | **United States** |
| --- | --- | --- | --- | --- | --- | --- | --- | --- |
| Discontinuation | ADHD x middle-aged adults | <0.001 | <0.001 | <0.001 | 0.41 | <0.001 | <0.001 | <0.001 |
|  | ADHD x older adults | <0.001 | 0.03 | <0.001 | – | <0.001 | 0.005 | <0.001 |
| Poor adherence (year 1) | ADHD x middle-aged adults | <0.001 | <0.001 | <0.001 | 0.57 | <0.001 | <0.001 | 0.34 |
|  | ADHD x older adults | <0.001 | 0.21 | 0.07 | – | <0.001 | 0.004 | 0.32 |
| Poor adherence (year 2) | ADHD x middle-aged adults | <0.001 | <0.001 | <0.001 | 0.61 | <0.001 | <0.001 | 0.24 |
|  | ADHD x older adults | 0.006 | 0.002 | 0.07 | – | <0.001 | 0.22 | 0.40 |
| Poor adherence (year 5) | ADHD x middle-aged adults | <0.001 | <0.001 | <0.001 | 0.15 | <0.001 | <0.001 | 0.02 |
|  | ADHD x older adults | 0.001 | <0.001 | 0.13 | – | <0.001 | 0.34 | 0.12 |

P-values were obtained from country-specific models including ADHD × age-group interaction terms. Young adults aged 18–34 years were used as the reference age group. Interaction terms were estimated for ADHD × middle-aged adults aged 35–64 years and ADHD × older adults aged ≥65 years. Models for discontinuation used Cox regression, and models for poor adherence used logistic regression. All models included ADHD, sex, age group, and calendar year at initiation of non-insulin antidiabetic medication, and ADHD × age-group interaction terms. Poor adherence was defined as proportion of days covered <80% over the corresponding 1-, 2-, or 5-year follow-up period. Norway had no older adult group because the oldest individuals in the birth cohort register were born in 1967. “–” indicates that the interaction term could not be estimated because the corresponding age group was unavailable.

B. ADHD × sex interaction tests

| **Outcome** | **Interaction term** | **Australia** | **Denmark** | **Netherlands** | **Norway** | **Sweden** | **UK** | **United States** |
| --- | --- | --- | --- | --- | --- | --- | --- | --- |
| Discontinuation | ADHD x sex | 0.07 | 0.02 | <0.001 | 0.99 | <0.001 | 0.27 | <0.001 |
| Poor adherence (year 1) | ADHD x sex | 0.01 | 0.53 | <0.001 | 0.34 | 0.06 | 0.19 | 0.007 |
| Poor adherence (year 2) | ADHD x sex | 0.004 | 0.28 | <0.001 | 0.66 | 0.03 | 0.48 | 0.01 |
| Poor adherence (year 5) | ADHD x sex | <0.001 | 0.45 | <0.001 | 0.82 | 0.30 | 0.66 | 0.56 |

P-values were obtained from country-specific models including ADHD × sex interaction term. Models for discontinuation used Cox regression, and models for poor adherence used logistic regression. All models included ADHD, sex, age, and calendar year at initiation of non-insulin antidiabetic medication, and ADHD × sex interaction term. Poor adherence was defined as proportion of days covered <80% over the corresponding 1-, 2-, or 5-year follow-up period.

# Supplementary Figure 1. Schematic illustration of time-varying ADHD medication exposure classification based on dispensation or prescription data.

This figure illustrates the approach used to classify ADHD medication use during follow-up. Because recorded dispensation/prescription duration was unavailable in most participating databases, treatment duration was estimated from dispensation or prescription records. For each record, the medication-use period started on the dispensing or prescription date and lasted for the country-, age-, and sex-specific 75th percentile of time to the subsequent ADHD medication record, calculated using only intervals ≤180 days. Early refills and overlapping records were carried forward to avoid double-counting covered days. Days within estimated medication-use periods were classified as on medication, and days outside these periods were classified as off medication. Switching between ADHD medications was not classified as off-medication time if medication coverage was continuous.

# Supplementary Figure 2. Schematic illustration of first discontinuation of any antidiabetic medication within five years of baseline.

This figure illustrates the approach used to define first discontinuation of any antidiabetic medication within five years of baseline.

The initial dispensation/prescription was non-insulin antidiabetic medication, followed by any other antidiabetic medication, including insulin. Continuous use was indicated by consecutive dispensations with interval of ≤135 days, a threshold derived from an assumed typical 90-day prescription length plus a 45-day grace period (one-half of the prescription length) to account for refill variability. Discontinuation of antidiabetic medication, defined using a refill gap method, occurred if the gap between consecutive dispensation dates exceeded 135 days. Medication switches were not classified as discontinuations. Time to discontinuation was defined as the time from the initiation date to the date of the estimated end of the last dispensation of continuous use.

# Supplementary Figure 3. Pooled and country-specific association between ADHD and poor adherence to antidiabetic medication in the exploratory model.

Main model, adjusted for sex, age and calendar year at initiation; and the exploratory model, additionally adjusted for psychiatric comorbidities. Poor adherence to antidiabetic medication was defined as proportion of days covered (PDC) <80%. Pooled estimates were obtained using random-effects meta-analysis across all seven countries.

# Supplementary Figure 4. Pooled and country-specific association between ADHD medication and poor adherence to antidiabetic medication in the exploratory model.

Our main model, adjusted for sex, age and calendar year at initiation; and the exploratory model, additionally adjusted for psychiatric comorbidities. Poor adherence to antidiabetic medication was defined as proportion of days covered (PDC) <80%. Pooled estimates were obtained using random-effects meta-analysis across all seven countries.

# Supplementary Figure 5. Unadjusted cumulative incidence of the first antidiabetic medication discontinuation across seven countries.

ADHD was modelled as a time-varying variable. The light blue area represents the 95% confidence interval for the non-ADHD group, and the light red area represents the 95% confidence interval for the ADHD group.

# Supplementary Figure 6. Pooled and country-specific association between ADHD and the first discontinuation of antidiabetic medication in the exploratory model.

Main model, adjusted for sex, age and calendar year at initiation; and the exploratory model, additionally adjusted for psychiatric comorbidities. *ADHD No., number of individuals diagnosed with ADHD or receiving ADHD medication by end of study period. Pooled estimates were obtained using random-effects meta-analysis across all seven countries.

# Supplementary Figure 7. Pooled association between ADHD and poor adherence to antidiabetic medication with and without Australia.

# Supplementary Figure 8. Pooled association between ADHD medication and poor adherence to antidiabetic medication with and without US.
